# Aberrant STAT signaling and T cell dysregulation define a targetable pediatric sepsis endotype

**DOI:** 10.1172/JCI202867

**Published:** 2026-06-09

**Authors:** Robert B. Lindell, Samir U. Sayed, Jose S. Campos Duran, Sydney A. Sheetz, Apoorva Babu, Montana S. Knight, Andrea A. Mauracher, Ceire A. Hay, Peyton E. Conrey, Julie C. Fitzgerald, Nadir Yehya, Stephen T. Famularo, Teresa Arroyo, Richard Tustin, Hossein Fazelinia, Edward M. Behrens, David T. Teachey, Lisa R. Forbes Satter, Alexandra F. Freeman, Jenna R.E. Bergerson, Steven M. Holland, Jennifer W. Leiding, Scott L. Weiss, Mark W. Hall, Deanne M. Taylor, Rui Feng, E. John Wherry, Nuala J. Meyer, Sarah E. Henrickson

**Affiliations:** 1 Division of Critical Care Medicine, Department of Anesthesia and Critical Care, Children’s Hospital of Philadelphia and the Perelman School of Medicine, University of Pennsylvania, Philadelphia, Pennsylvania, USA.; 2 Institute for Immunology and Immune Health, Perelman School of Medicine, University of Pennsylvania, Philadelphia, Pennsylvania, USA.; 3 Division of Allergy and Immunology, Department of Pediatrics,; 4 Division of Critical Care Medicine, Department of Anesthesia and Critical Care, and; 5 Department of Biomedical and Health Informatics, Children’s Hospital of Philadelphia, Philadelphia, Pennsylvania, USA.; 6 Division of Rheumatology, Department of Pediatrics and; 7 Division of Oncology, Department of Pediatrics, Children’s Hospital of Philadelphia and the Perelman School of Medicine, University of Pennsylvania, Philadelphia, Pennsylvania, USA.; 8 Division of Immunology, Allergy, and Retrovirology, Department of Pediatrics, Texas Children’s Hospital and Baylor College of Medicine, Houston, Texas, USA.; 9 Laboratory of Clinical Immunology and Microbiology, National Institute of Allergy and Infectious Diseases, NIH, Bethesda, Maryland, USA.; 10 Division of Allergy and Immunology, Department of Pediatrics, Johns Hopkins University School of Medicine and Johns Hopkins All Children’s Hospital, St. Petersburg, Florida, USA.; 11 Division of Critical Care, Department of Pediatrics, Nemours Children’s Health and Sidney Kimmel Medical College at Thomas Jefferson University, Wilmington, Delaware, USA.; 12 Division of Critical Care, Department of Pediatrics, Nationwide Children’s Hospital and The Ohio State University College of Medicine, Columbus, Ohio, USA.; 13 Department of Biomedical and Health Informatics, Children’s Hospital of Philadelphia, Philadelphia, Pennsylvania, USA.; 14 Department of Pediatrics,; 15 Department of Biostatistics, Epidemiology, and Informatics,; 16 Department of Systems Pharmacology and Translational Therapeutics, and; 17 Division of Pulmonary, Allergy, and Critical Care Medicine, Department of Medicine, Perelman School of Medicine, University of Pennsylvania, Philadelphia, Pennsylvania, USA.; 18 Division of Allergy and Immunology, Department of Pediatrics, Children’s Hospital of Philadelphia and the Perelman School of Medicine, University of Pennsylvania, Philadelphia, Pennsylvania, USA.; 19 Department of Microbiology, Perelman School of Medicine, University of Pennsylvania, Philadelphia, Pennsylvania, USA.

**Keywords:** Clinical Research, Immunology, Inflammation, Bioinformatics, Cellular immune response, Proteomics

## Abstract

**BACKGROUND:**

Sepsis is a leading cause of morbidity and mortality in critically ill children, yet heterogeneous immune responses complicate the development of targeted therapies and the host immune factors driving sepsis pathobiology remain unclear.

**METHODS:**

We integrated deep immune phenotyping, plasma proteomics, single-cell transcriptomics, and phosphoflow cytometry in a prospective cohort of 88 critically ill children to elucidate the mechanisms underlying immune heterogeneity.

**RESULTS:**

Unsupervised clustering of plasma cytokines identified 3 immunologic subgroups, including a high-severity group (“Group C”) characterized by hypercytokinemia driven by IL-6 and IFN-γ. Group C exhibited distinct alterations in immune cell frequency and activation, with a strong association between hyperinflammatory cytokine signaling and lymphocyte dysfunction. Single-cell RNA-seq revealed transcriptional signatures of T cell activation and metabolic stress, with suppression of a lymphoid protective gene program across CD8^+^ T cell subsets. Despite increased expression of activation markers, T cell receptor repertoire analysis revealed no dominant clonotypes, consistent with bystander activation. Phosphoflow cytometry demonstrated baseline STAT1/STAT3 hyperactivation in Group C CD8^+^ T cells, which failed to respond to αCD3/αCD28/αCD49d stimulation.

**CONCLUSIONS:**

These findings define an IL-6/IFN-γ–driven endotype of T cell dysfunction in pediatric sepsis and highlight the JAK/STAT axis as a rational target for immunomodulatory therapy.

**FUNDING:**

K12HD047349, K23GM159013, K08AI135091, R01HD095976, Thrasher Research Fund, Burroughs Wellcome Fund, Immune Deficiency Foundation, Primary Immune Deficiency Treatment Consortium, Barbara Brodsky Foundation, CHOP Research Institute.

## Abstract 2

We identified a unique immune profile in children with sepsis linked to outcomes, providing a biological fingerprint to help guide future personalized treatments.

## Introduction

Sepsis, a life-threatening organ dysfunction that develops in the setting of a dysregulated immune response to infection (1), remains a leading cause of adult and pediatric mortality worldwide (2). Unlike adults, pediatric sepsis deaths occur predominantly in children with immunocompromised diagnoses (3–6) and in the setting of persistent multiple organ dysfunction syndrome (MODS) (7, 8). Sepsis is the most common cause of MODS in children (9, 10) representing a “final common pathway” where systemic inflammation drives progressive organ failure via endothelial, epithelial, mitochondrial, and acquired immunologic dysfunction (11–13).

Identifying modifiable molecular mechanisms is essential for translating biologic insights into precision therapeutics (14). While innate, adaptive, and mitochondrial dysfunction are strongly associated with adverse outcomes in pediatric sepsis (15–20), successful immune modulation in sepsis has been elusive (21). Trials of targeted immunomodulation have failed to improve survival in adults (22–24), and the molecular mechanisms driving adverse sepsis outcomes remain unclear (25).

A successful precision medicine framework for pediatric sepsis will require an understanding of the molecular events that precipitate organ failure, knowledge of which events are reversible, and the ability to identify high-risk patients in real time (26). While clinical sepsis phenotypes can stratify risk (27–29), they lack mechanistic resolution to guide treatment decisions. Recent molecular profiling studies in adult sepsis have illuminated pathway-specific heterogeneity (30–34), and retrospective reanalysis of negative sepsis trials have demonstrated heterogeneity of treatment effect across biomarker-defined sepsis subgroups, with benefit in hyperinflammatory patients and harm in hypoinflammatory patients (35). Pediatric sepsis, with a lower burden of chronic comorbidities, offers an optimal cohort for resolving these biological signals and defining treatable endotypes.

We designed a longitudinal multi-omics cohort study of pediatric patients with MODS with and without sepsis to identify immunologic endotypes enriched in high-severity patient subgroups. We hypothesized that severity-associated subgroups would reflect distinct, mechanistically defined immune programs. Using integrated longitudinal proteomic, cytometric, and transcriptomic profiling, we defined 3 prognostic subgroups and identified dysregulated STAT1/STAT3 signaling as a targetable driver of CD8^+^ T cell hyperactivation in the highest-risk group.

## Results

### Proteomic heterogeneity of pediatric critical illness.

**Figure 1:**
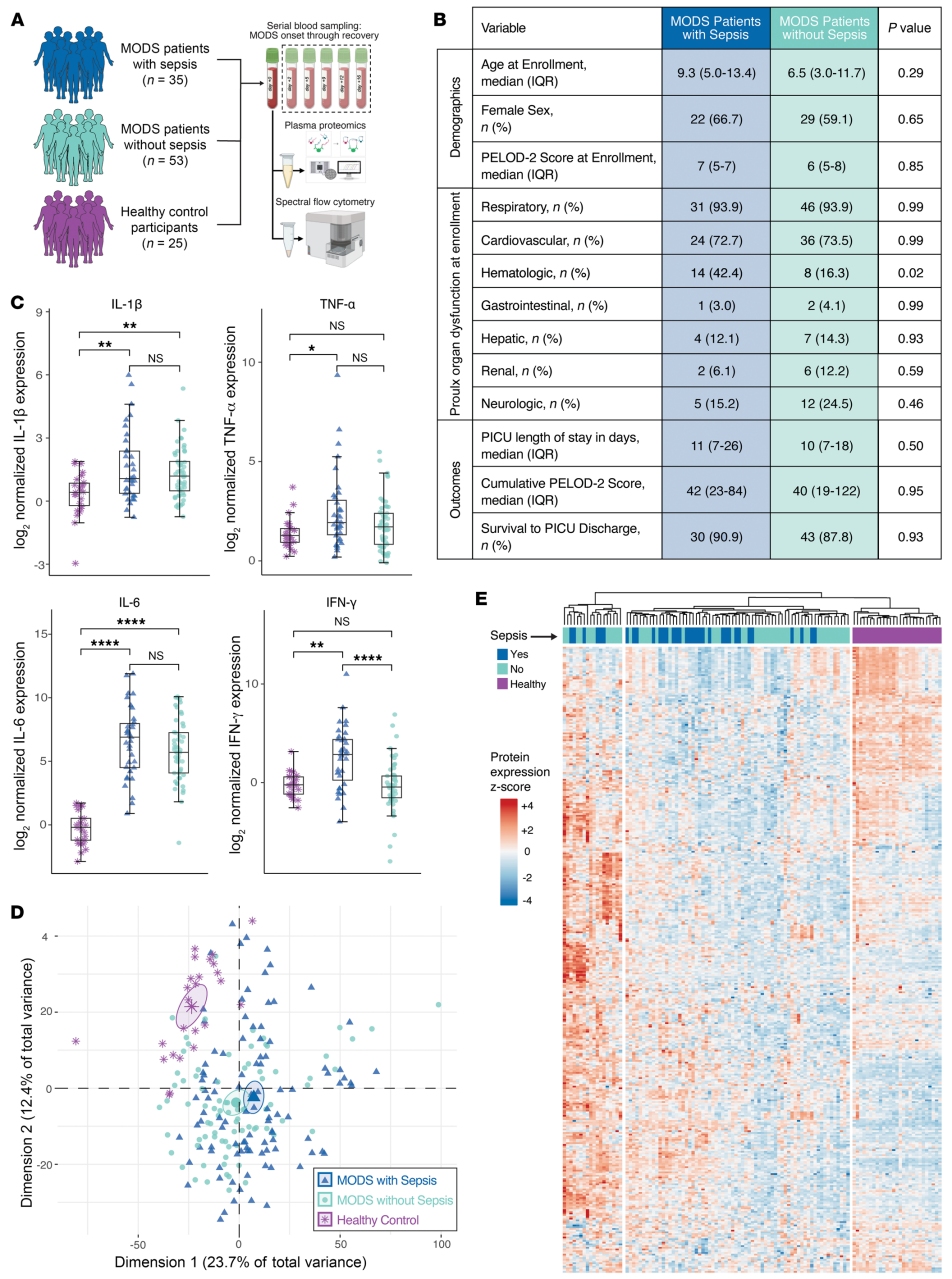
Shared proteomic features in pediatric patients with MODS with and without sepsis. (**A**) Study schematic showing patients with MODS with sepsis (*n* = 35) and without sepsis (*n* = 53), as well as participants in the healthy control (HC) group (*n* = 25). (**B**) Patient demographics and clinical outcomes, stratified by etiology (sepsis versus nonsepsis). Comparisons by χ² or Wilcoxon rank-sum test. (**C**) Proinflammatory cytokines are elevated in MODS relative to participants in the HC group. Box-and-whisker plots show median and IQR (whiskers 1.5 × IQR). Comparisons by Kruskal-Wallis test followed by Dunn’s post hoc test with Benjamini-Hochberg adjustment: *P < 0.05, **P < 0.01, ***P < 0.001, ****P < 0.0001. (**D**) PCA of 1,448 plasma proteins from 88 patients with MODS and 25 participants in the HC group demonstrates overlap between patients with MODS with and without sepsis. (**E**) Hierarchically clustered heatmap of 1,448 row-normalized proteins measured at MODS onset. HC samples cluster separately from MODS samples, and patients with MODS with and without sepsis exhibit overlapping proteomic profiles. Annotation bars indicate cohort membership.

To identify endotypes in patients with and without sepsis, we collected peripheral whole blood samples in a prospective, observational cohort of 88 pediatric patients with MODS. Blood was collected within 48 hours of MODS onset and then twice weekly through death or resolution of MODS. At each timepoint, peripheral blood mononuclear cells (PBMC) and heparinized plasma biospecimens were cryopreserved for later analysis (Figure 1A). We compared these patients with a separate cohort of 25 participants who were pediatric healthy controls (HCs). Our initial analyses included a 1,536-marker proteomics panel from Olink Proteomics (36) (Supplemental Table 1; supplemental material available online with this article; https://doi.org/10.1172/JCI202867DS1) and a 35-marker spectral flow cytometry–based immune phenotyping panel (Supplemental Table 2).

Demographics and clinical outcomes were similar between our 35 patients with sepsis and 53 patients without sepsis (Figure 1B). The only organ dysfunction category that differed between groups was hematologic dysfunction, driven by thrombocytopenia at MODS onset in patients with sepsis (platelet count median [IQR]: 119 [IQR: 99–187] versus 199 [IQR: 131–278], *P* < 0.001; normal range 150–450 × 10^3^/μL). Primary and secondary patient outcomes did not differ between MODS subgroups, including cumulative Pediatric Logistic Organ Dysfunction-2 (PELOD-2) organ dysfunction score, survival to pediatric intensive care unit (PICU) discharge, and duration of PICU stay.

We hypothesized that expression of proinflammatory cytokines would differ between patients with and without sepsis. While we noted the expected increased proinflammatory cytokines (IL-1β, IL-6, tumor necrosis factor (TNF), IL-18, monocyte chemoattractant protein–1 (MCP-1), IFN-γ, all *P* < 0.05) in patients with sepsis compared with participants in the HC group, most cytokines were also markedly elevated in patients with MODS without sepsis. We found that median IL-1β, TNF-α, and IL-6 levels did not differ between patients with and without sepsis, while IFN-γ was higher in patients with sepsis (*P* < 0.0001) (Figure 1C). Principal component analysis (PCA) distinguished patients with MODS from HCs, but revealed substantial overlap between sepsis and nonsepsis populations (Figure 1D). Patients with MODS also exhibited substantial heterogeneity along PC1 and PC2. These dimensions were shaped by proteins involved in biologic processes such as growth regulation (e.g., MAD1L1, HDGF), inflammatory signaling (e.g., CASP3, MAP3K5), and endothelial dysfunction (e.g., PLAUR, SRC), as illustrated by the top PCA loadings (Supplemental Table 3).

Hierarchical clustering of row-normalized protein expression similarly distinguished patients with MODS from healthy controls but failed to discriminate between sepsis and nonsepsis populations (Figure 1E). This overlap, complementary with the PCA findings, suggests that MODS may represent a final common pathway of critical illness, independent of the inciting diagnosis or infection status.

### Identification of 3 severity-associated subgroups.

**Figure 2:**
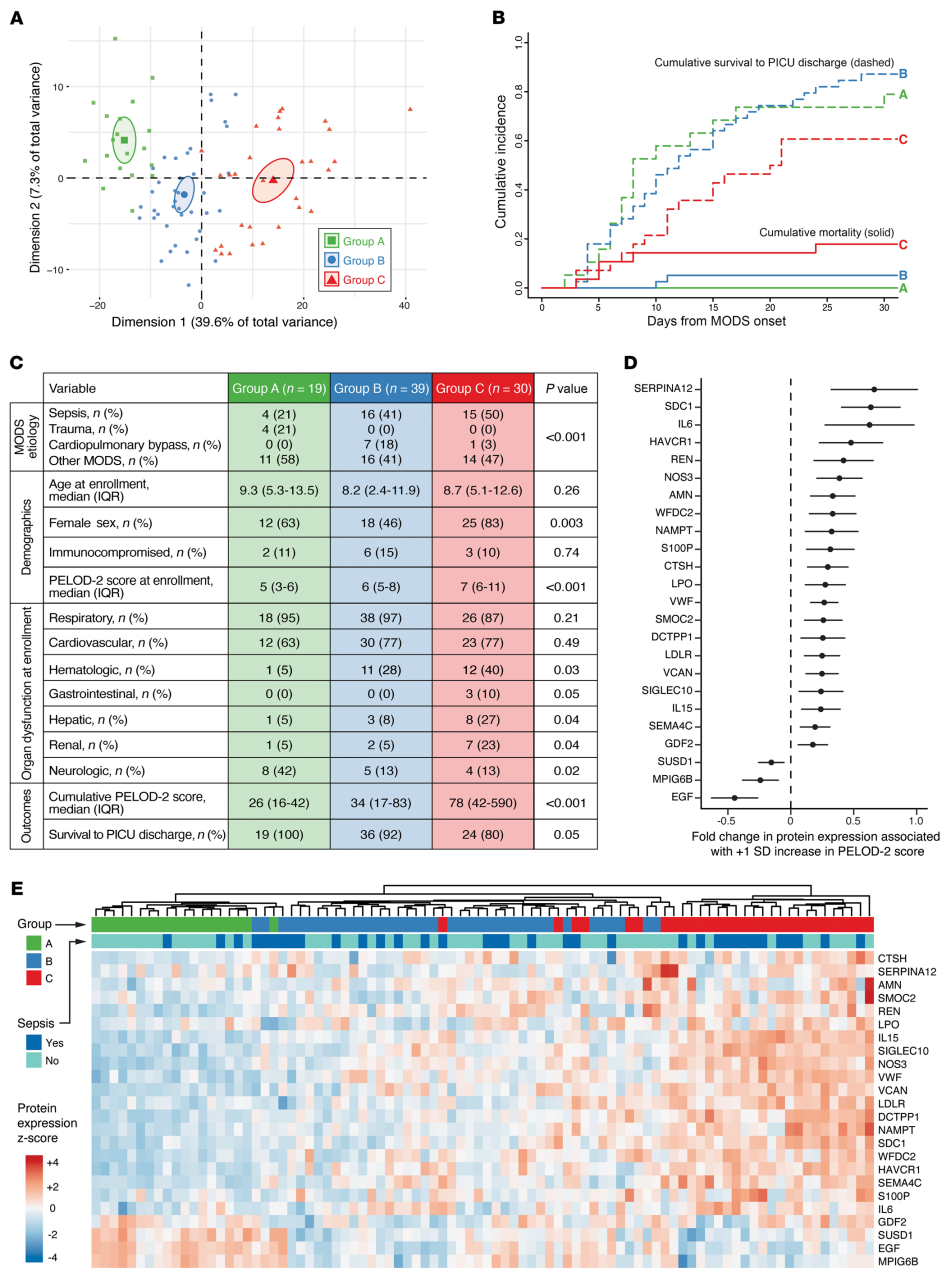
Three subgroups based on protein expression at MODS onset. (**A**) PCA of 214 severity-associated plasma proteins in 88 patients at MODS onset demonstrating separation of 3 subgroups defined by consensus clustering. (**B**) Cumulative incidence of survival to PICU discharge (dashed) and PICU mortality (solid) by subgroup, estimated using the Fine-Gray model. (**C**) Cohort demographics, etiology, and clinical outcomes, stratified by proteomic subgroup. Comparisons by χ² or Wilcoxon rank-sum test. (**D**) Fold-change in expression per +1 SD increase in PELOD-2 score for 24 proteins selected by elastic net regression. Points represent fold-change estimates; whiskers are 95% confidence intervals. (**E**) Hierarchically clustered row-normalized heatmap of the 24-protein elastic net signature in 88 patients at MODS onset. Annotation bars indicate subgroup assignment and sepsis status.

To uncover biologic programs driving organ dysfunction in children with MODS, we first sought to define severity-associated subgroups based on plasma protein expression to enrich for mechanistic signal. Because sepsis immune responses vary by age, sex, and time (16), we used linear mixed-effects models to identify plasma proteins associated with severity of illness, defined by the PELOD-2 organ dysfunction score, after adjustment for age and sex, as fixed effects and day from MODS onset as a random effect. This yielded a set of 214 plasma proteins that were significantly associated with illness severity (Supplemental Table 4). We then employed consensus clustering (37) to identify distinct subgroups of patients based on expression of these severity-associated proteins (Figure 2A). Optimal cluster number (*k*=3) was identified via Monte Carlo bootstrapping and confirmed by elbow method and gap statistic (Supplemental Figure 1). Sensitivity analysis using spectral clustering confirmed the robustness of these severity-associated subgroups (adjusted Rand index = 0.73, concordance = 91%; Supplemental Figure 2). To understand the association between subgroups and clinical outcomes, we tested the effect of subgroup membership on the cumulative incidence of both mortality and survival to PICU discharge with the Fine-Gray subdistribution hazard model (38). In this survival analysis, patients in Group C have a higher cumulative incidence of death (*P* = 0.03) and a lower cumulative incidence of survival to PICU discharge (*P* = 0.04) compared with patients in Groups A and B, with separation occurring in the first week and persisting to day +28 (Figure 2B). Group B demonstrated an intermediate clinical trajectory, with survival rate and cumulative PELOD-2 organ dysfunction score falling between those of Groups A and C.

Though defined by protein expression alone, these identified subgroups also differ by clinical features and outcomes (Figure 2C). MODS etiology varies by Group, with similar proportions of patients with sepsis in Group B and C and all patients with trauma classified in Group A. Patients in Group C have higher severity of illness, more noncardiopulmonary organ failures, and increased cumulative organ dysfunction scores and mortality compared with Groups A/B. The etiology of MODS and computed subgroup for each patient are detailed in Supplemental Table 5. Preexisting immunocompromised status, defined a priori as active malignancy, hematopoietic cell transplantation, or primary immunodeficiency (39), was identified in 13% of patients with MODS (11/88), and these patients were not imbalanced across subgroups (*P* = 0.74), as detailed in Supplemental Table 6. Patients were exposed to corticosteroids at a similar rate across subgroups (A: 10/19 [53%], B: 13/39 [33%], C: 14/30 [47%], *P* = 0.31), suggesting that group assignment was not driven by treatment allocation.

Noting an ordinal increase in the number of organ failures and mortality across plasma protein–derived subgroups, we hypothesized that a reduced set of proteins could identify these subgroups and could be more suitable for translation to the clinical setting. Elastic net regularization is a common approach to generate a high-performing sparse model with good predictive accuracy (40). To define a parsimonious protein signature, we trained an ordinal elastic net model (41) with 10-fold cross validation and generated a 24-protein signature that discriminates the 3 subgroups (Supplemental Figure 3).

To evaluate the performance of this parsimonious model, we first tested the association between the elastic net proteins and severity of illness. Using a linear mixed-effects model, we estimated the fold change in protein expression associated with a 1-SD change in PELOD-2 organ dysfunction score (Figure 2D). This model demonstrates that modest changes in expression of these 24 proteins are associated with meaningful differences in illness severity. Second, we tested the discrimination of this 24-protein signature. Hierarchical clustering of elastic net proteins at MODS onset effectively separates subgroups but does not discriminate between patients with and without sepsis (Figure 2E).

To quantify performance of the elastic net protein set, we calculated the polytomous discrimination index (PDI), a measure of rank-based discrimination, for each subgroup. Category-specific PDI demonstrated excellent discrimination for each subgroup (Group A: 0.98, Group B: 0.96, Group C: 0.99), and the overall PDI for the model was 0.98. Taken together, these results suggest that this 24-protein signature reflects severity of illness and successfully discriminates subgroups at MODS onset.

### Immune cell frequency and activation vary by subgroup.

Having identified 3 severity-associated subgroups (Groups A/B/C) from protein expression data and built a parsimonious classification model, we next sought to define underlying mechanisms of immune dysregulation. We hypothesized that protein-derived subgroups would be associated with differences in cellular immunophenotype. For this analysis, we performed high-dimensional spectral flow cytometry on cryopreserved PBMCs obtained at MODS onset using a custom-designed 35 marker panel (Supplemental Table 2), which includes both phenotypic and functional markers. After arcsinh scaling (42) and quality control with flowAI (43), we performed FlowSOM metaclustering (44) and identified 14 immune cell populations by surface and intracellular marker expression. Supplemental Figure 4 presents a representative example of our manual gating strategy for this immune phenotyping panel, which we used to confirm the identity of the FlowSOM metaclusters, as well as representative immunophenotyping plots.

**Figure 3:**
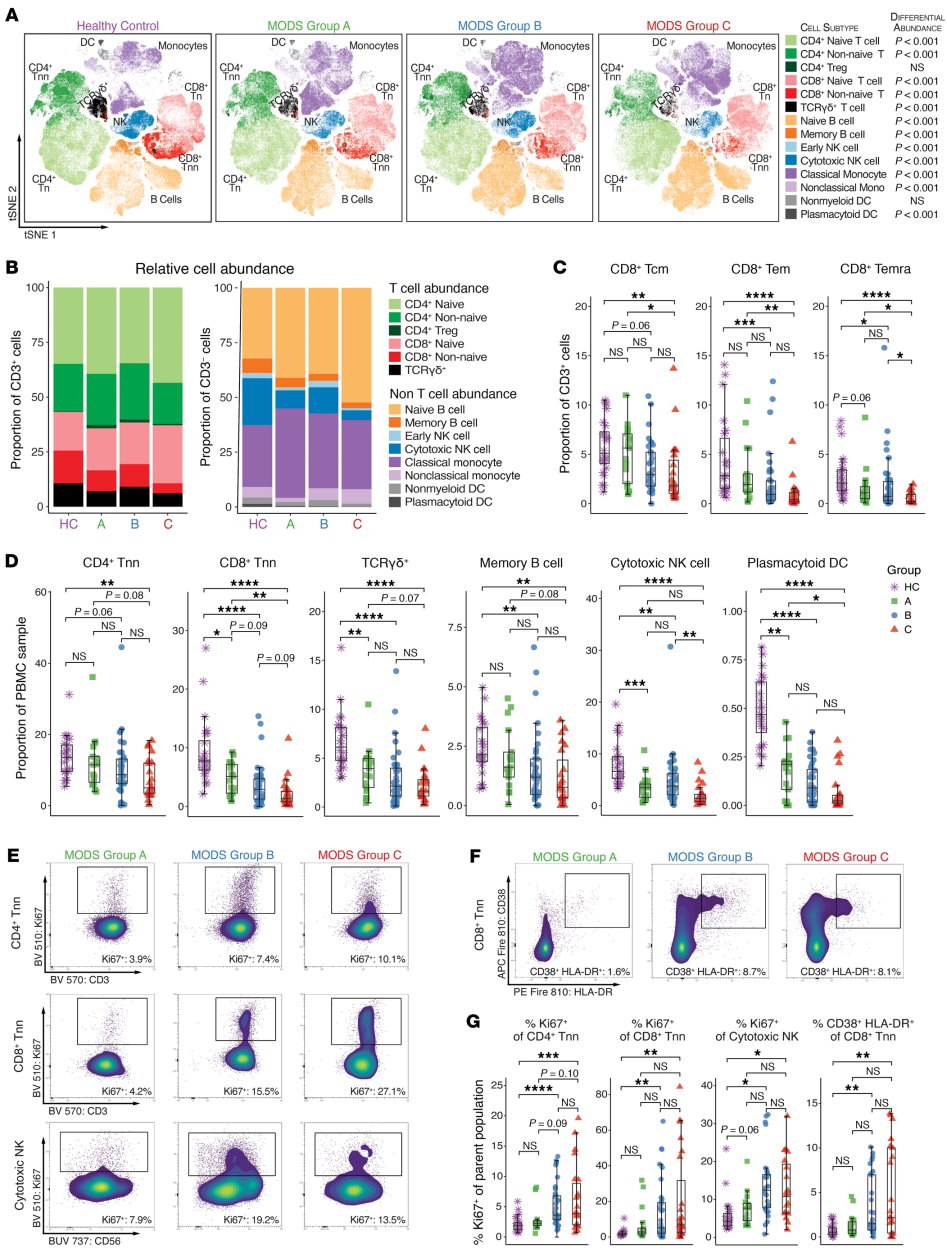
Immune cell abundance and activation state differ across proteomic subgroups. (**A**) tSNE-CUDA projection of PBMC phenotypes (14 FlowSOM-defined metaclusters) from participants in the HC group and patients with MODS. (**B**) Stacked bar plots showing proportional abundance of CD3^+^ and CD3^–^ cell populations by subgroup. (**C**) Reduced frequency of CD8^+^ central memory, effector memory, and Temra cells in MODS, with an ordinal decrease across subgroups. (**D**) Proportional abundance of additional lymphoid and myeloid populations in MODS. (**E**) Ki67 expression in nonnaive CD4^+^ T cells, nonnaive CD8^+^ T cells, and cytotoxic NK cells is increased in Groups B and C relative to Group A (all *P* < 0.05), indicating increased proliferation. (**F**) Representative concatenated flow plots of Ki67 expression in nonnaive T cells and cytotoxic NK cells. (**G**) Quantification of proliferation and activation markers by patient and subgroup. Box-and-whisker plots show median and IQR (whiskers 1.5 × IQR). Comparisons by Kruskal-Wallis test followed by Dunn’s post hoc test with Benjamini-Hochberg adjustment: *P < 0.05, **P < 0.01, ***P < 0.001, ****P < 0.0001.

To measure the immunophenotypic differences between participants in the HC group and patients in the 3 subgroups (Figure 3A), we subsampled the data to 100,000 cells per subgroup and applied t-distributed Stochastic Neighbor Embedding with Compute Unified Device Architecture (tSNE-CUDA) dimensionality reduction (45). Differences in cell populations by subgroup are quantified in stacked bar plots (Figure 3B). The proportional abundance of central memory (CD45RA^–^CD27^+^), effector memory (CD45RA^–^CD27^–^), and Temra (CD45RA^+^CD27^–^) CD8^+^ T cells were markedly reduced in patients in Group C (Figure 3C), and this proportional loss of nonnaive CD8^+^ T cells was strongly associated with severity of illness by linear regression (*P* < 0.001). A similar ordinal trajectory was identified across multiple cell types (Figure 3D), including T cells, B cells, NK cells, and dendritic cells (Cuzick test of trend *P* < 0.001 for each cell type shown), and reduction in frequency of each of these cell types was strongly associated with severity of illness by linear regression (all *P* < 0.001).

In addition to shifts in peripheral immune cell subset frequency, we hypothesized that subgroups would be associated with differences in immune cell activation, as measured by expression of markers of proliferation (i.e., Ki67) and activation (i.e., CD38 and HLA-DR), across subgroups. Representative bivariate plots of Ki67 expression in nonnaive CD4^+^ and CD8^+^ T cells and cytotoxic NK cells (CD56^dim^ CD16^+^) demonstrate increased proliferation in cells from patients in Group B and Group C compared with cells from patients in Group A (Figure 3E). Similarly, CD38 and HLA-DR coexpression in nonnaive CD8^+^ T cells was markedly increased in cells from patients in Group B and Group C compared with cells from patients in Group A, but not different between Group B and Group C, indicative of CD8^+^ T cell activation in Groups B and C (Figure 3F). Corresponding boxplots in Figure 3G quantify differences in proliferation and activation by patient and subgroup. Despite limited pairwise separation between Groups B and C, we observed a monotonic increase in the proportion of activated cells across subgroups (Cuzick test of trend *P* < 0.001 for each cell type shown), indicating a graded activation trajectory across groups. Taken together, these data indicate that nonnaive CD8^+^ T cells in Group C exhibit the greatest degree of lymphodepletion and immune activation, and that these cellular phenotypes are associated with severity of illness.

### Multiple concurrent mechanisms of immune dysregulation converge in patients in Group C.

**Figure 4:**
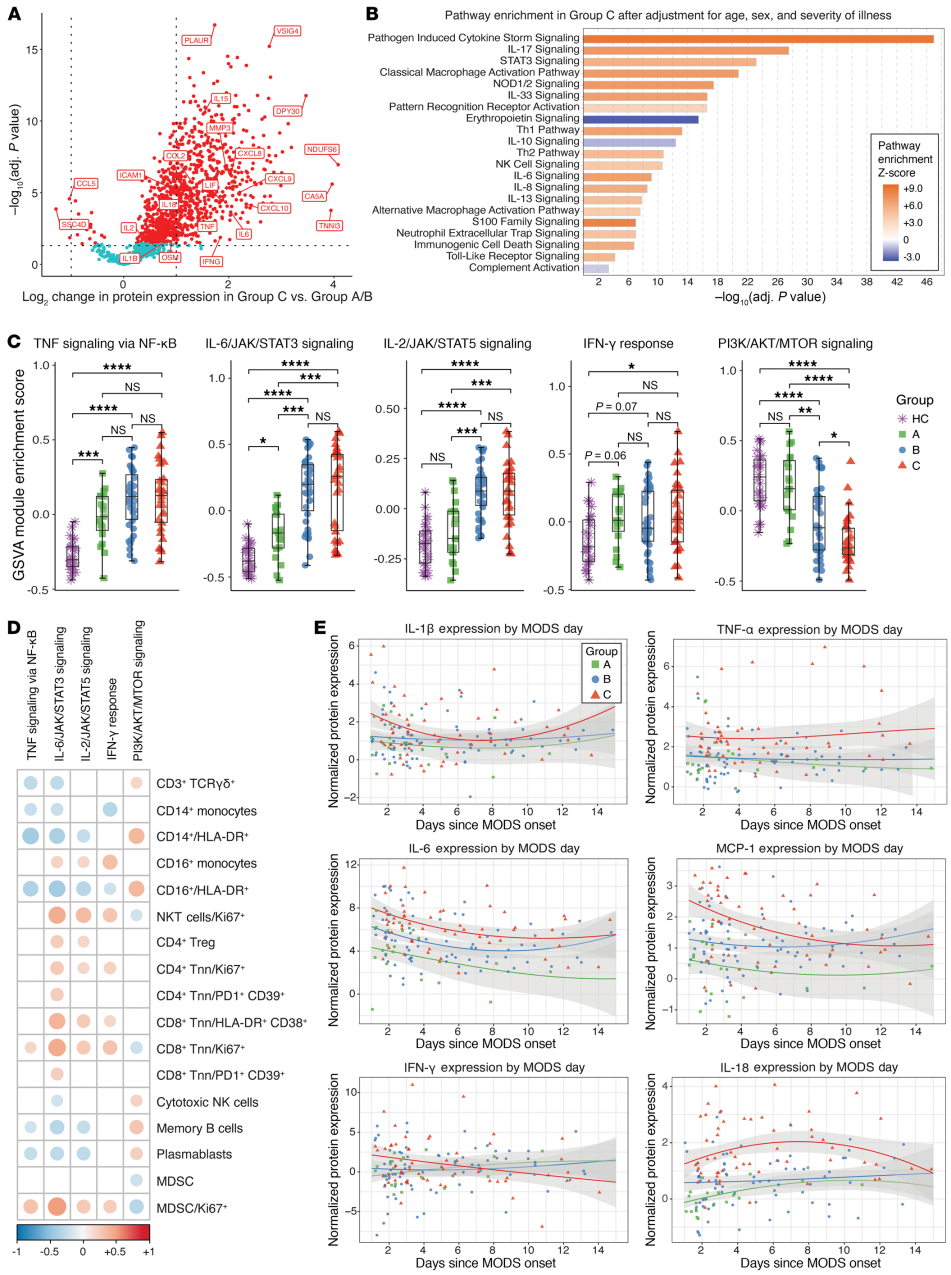
Patients in Group C exhibit a hyperinflammatory plasma with concurrent activation of multiple immune dysregulation pathways. (**A**) Volcano plot of plasma protein expression in Group C versus Groups A/B at MODS onset (FDR <0.05). (**B**) Ingenuity Pathway Analysis of 1,003 differentially expressed proteins after adjustment for age, sex, severity of illness, and days since MODS onset using linear mixed-effects modeling. (**C**) GSVA pathway enrichment scores using Hallmark gene sets. Group B and Group C showed markedly increased IL-6/JAK/STAT3 and IL-2/JAK/STAT5 signaling and an ordinal decrease in PI3K/AKT/mTOR signaling across subgroups. Box-and-whisker plots show median and IQR (whiskers 1.5 × IQR). Comparisons by Kruskal-Wallis test followed by Dunn’s post hoc test with Benjamini-Hochberg adjustment: *P < 0.05, **P < 0.01, ***P < 0.001, ****P < 0.0001. (**D**) Spearman correlation matrix between GSVA scores and immune cell phenotypes (FDR *P* < 0.05). Circle color reflects correlation coefficient (ρ; −1 to +1) and size indicates significance. (**E**) Longitudinal expression of canonical cytokine storm–associated proteins over 14 days (LOESS regression with 95% CI).

Because patients in Group C represent the most severe manifestation along a continuum of severity-associated immune dysregulation, we hypothesized that we could identify candidate mechanisms contributing to this phenotype by measuring differential expression of key canonical inflammatory pathways in patients in Group C. Using the plasma proteomics dataset, we first examined differential protein expression after adjustment for patient age, sex, severity of illness, and days since MODS onset using a linear mixed-effects model. In unadjusted analysis, 1,061 of 1,448 measured proteins were differentially expressed in Group C (Figure 4A), and in our adjusted model 1,003 of 1,061 proteins remained differentially expressed compared with Group A/B. For 98% (980/1,003) of these differentially expressed proteins, expression was upregulated in Group C.

To compare expression of key canonical inflammatory pathways across subgroups, we performed pathway enrichment analysis of our adjusted plasma protein expression dataset using complementary group- and patient-based strategies. First, we used Ingenuity Pathway Analysis (IPA, Qiagen) (46) to identify enrichment of 22 canonical pathways in the Group C proteome in comparison with Group A/B (Figure 4B). Noting that 8 of the top 10 differentially expressed pathways identified by IPA were related to hyperinflammatory signaling, we then applied Gene Set Variation Analysis (GSVA) (47) to study enrichment of 5 canonical proinflammatory pathways on the individual patient level using Human Molecular Signatures Database (MSigDB) Hallmark gene sets (48). Using GSVA, we assigned patient-level protein module enrichment scores for each pathway of interest based on expression of proteins in the corresponding Hallmark gene set, as detailed in Methods. Module enrichment scores for each pathway are visualized in Figure 4C and demonstrate markedly increased IL-6/JAK/STAT3 and IL-2/JAK/STAT5 module enrichment in patients in Group B and Group C (each *P* < 0.001 vs Group A) and an ordinal decrease in PI3K/AKT/mTOR signaling across subgroups (Cuzick test of trend *P* < 0.001). Plasma IFN-γ response and TNF/NF-κB signaling module enrichment scores did not vary by subgroup. These data indicate that aberrant immune cell signaling is not restricted to a single subgroup but increases along a continuum of immune activation, with Group C representing the most severe manifestation of immune dysregulation within our cohort.

We then assessed the correlation between Hallmark module enrichment and immune cell subset proportional abundance and activation in patients with MODS (Figure 4D). We noted that IL-6/JAK/STAT3 module enrichment had the strongest correlation with proliferation and activation of immune cell subsets previously identified (Figure 3, E and F), including nonnaive CD4^+^ and CD8^+^ T cell proliferation (Ki67 expression) and activation (HLA-DR/CD38 coexpression and PD-1/CD39 coexpression). Classical and nonclassical monocyte HLA-DR expression was inversely correlated with IL-6/JAK/STAT3 module enrichment score, consistent with the well-established paradigm of sepsis-associated immunoparalysis, while immune regulatory cell populations (regulatory T cells, myeloid-derived suppressor cells) were positively correlated with IL-6/JAK/STAT3 module enrichment score.

Finally, we studied the longitudinal expression of cytokine storm markers (49) in patients with MODS to understand the duration of cytokinemia in patients in Group C. Normalized protein expression by day since MODS onset by subgroup is shown in Figure 4E. We noted that IL-6, MCP-1, and IL-18 expression were significantly higher in patients in Group C for the first 7 days after MODS onset (each *P* < 0.001 at day +7), while IL-1β and TNF-α expression remained different for only the first 4 days (*P* = 0.05 and *P* = 0.009 respectively at day +4) and IFN-γ expression did not differentiate subgroups at any timepoint, though these differences may reflect differential plasma stability or tissue consumption rather than upstream production mechanisms.

### Immune dysregulation in MODS compared with patients with inborn errors of immunity that impact STAT1 and STAT3 signaling.

The enrichment of STAT pathway signaling in patients with the most severe MODS and its association with increased T cell activation by flow cytometry prompted us to ask whether this endotype reflects a physiologic adaptation to critical illness or a maladaptive, pathologic form of immune dysregulation. STAT3 is a central mediator of inflammatory signaling, implicated in endothelial dysfunction, capillary leak, emergency granulopoiesis, and disrupted lymphocyte homeostasis (50). Recent translational studies have highlighted its role in human sepsis, including the identification of immature circulating CD66b^+^ neutrophils as markers of STAT3-driven emergency myelopoiesis in adults who are critically ill (51), and 2 recent preclinical studies have demonstrated a protective effect of selective STAT3 inhibition in mice with CLP-induced sepsis (52, 53). Informed by these studies, we hypothesized that the magnitude of STAT3 pathway activation in patients in Group C is not merely an epiphenomenon but instead reflects a pathogenic immune program that may constitute a viable target for precision immunomodulation.

To test this hypothesis, we compared plasma proteomic profiles between patients with MODS and participants with rare, monogenic inborn errors of immunity (IEI) involving amplified or impaired activation of the STAT3 and STAT1 signaling pathways, which predispose to immune dysregulation and susceptibility to infection. In this analysis, IEIs offer a unique lens through which to interpret complex immune phenotypes, serving as arbiters that reveal the immunologic consequences of discrete signaling perturbations in vivo. Patients with 4 different IEI were included in this analysis: STAT1 gain-of-function (GOF) (*n* = 9), STAT3 GOF (*n* = 5), STAT1 autosomal dominant and dominant-negative (DN) (*n* = 1), or STAT3 DN (*n* = 3). We measured protein expression using a 384-marker inflammatory proteomics panel from Olink Proteomics (Supplemental Table 7), and we analyzed pathway expression in bulk using Gene Set Enrichment Analysis (GSEA) (54) and at the individual patient level using GSVA (47).

**Figure 5:**
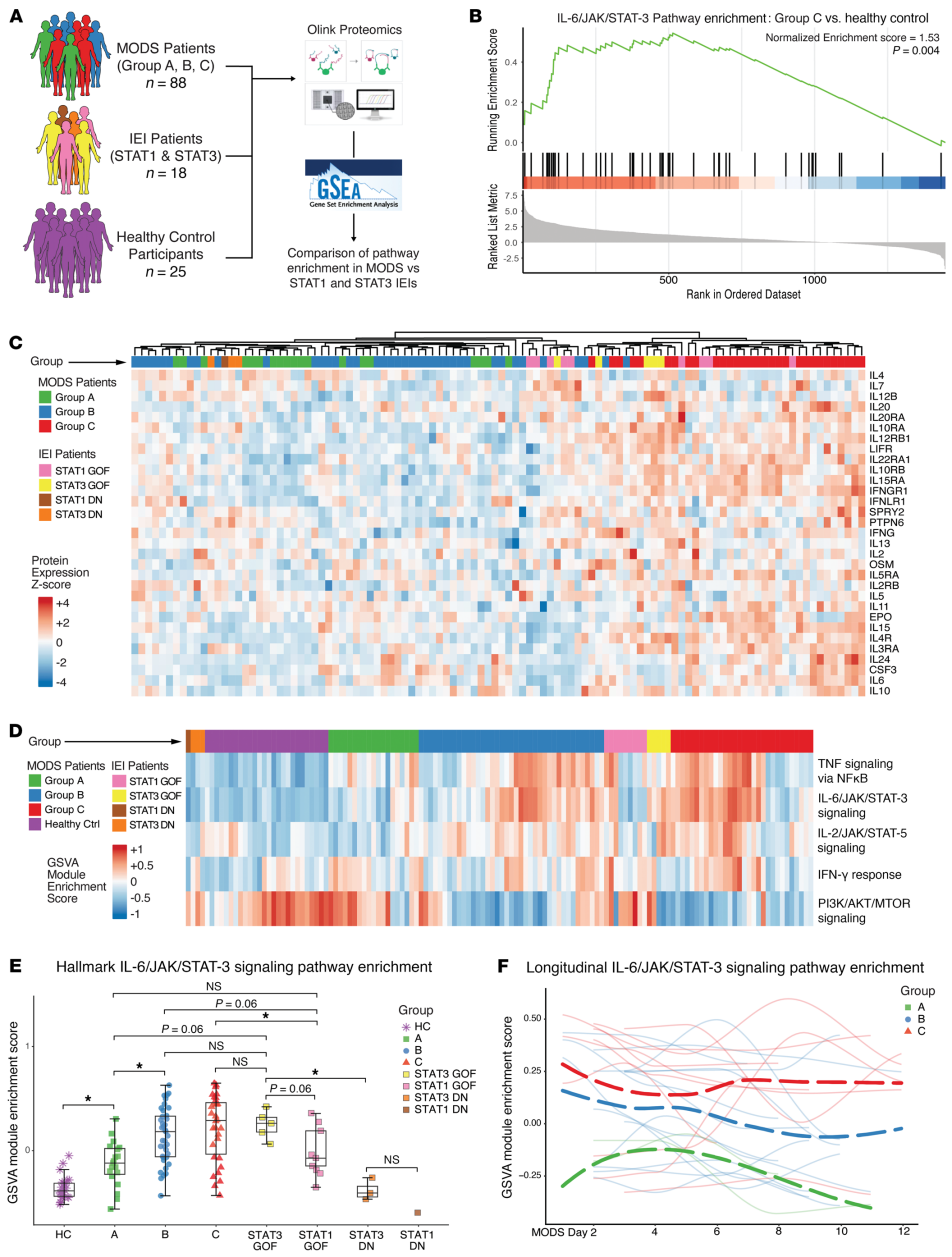
STAT3 pathway activation in patients in Group C meets or exceeds that observed in monogenic STAT1/STAT3 immune disorders. (**A**) Comparative analysis of MODS (*n* = 88), healthy control (HC, *n* = 25), and inborn error of immunity (IEI, *n* = 18) cohorts, including STAT1/STAT3 gain-of-function (GOF) and dominant-negative (DN) mutations. (**B**) GSEA of Hallmark IL-6/JAK/STAT3 pathway (Group C versus HC). (**C**) Row-normalized heatmap of 31 KEGG JAK/STAT target proteins in hierarchically clustered patients with MODS and IEI. Annotation bars denote IEI diagnosis and MODS subgroup. (**D**) Row-normalized heatmap of patient-level GSVA of 5 inflammatory Hallmark pathways. (**E**) Comparison of Hallmark IL-6/JAK/STAT3 GSVA scores across MODS and IEI groups. Box-and-whisker plots show median and IQR (whiskers 1.5 × IQR). Comparisons by Kruskal-Wallis test followed by Dunn’s post hoc test with Benjamini-Hochberg adjustment: *P < 0.05, **P < 0.01, ***P < 0.001, ****P < 0.0001. (**F**) Longitudinal IL-6/JAK/STAT3 enrichment (*n* = 26) in MODS patients with greater than or equal to 3 serial samples with individual patient trajectories (solid) and LOESS regression (dashed) shown within each subgroup.

A summary of our experimental design is shown in Figure 5A, which includes 88 patients with MODS, 18 patients with IEI, and 25 participants in the HC group. Orthogonal to our Ingenuity Pathway Analysis findings, we first confirmed population-level proteomic enrichment (normalized enrichment score 1.53, *P* value 0.004) of the Hallmark IL-6/JAK/STAT3 signaling pathway in Group C patients at MODS onset compared with participants in the HC group using GSEA (Figure 5B). Leading edge proteins and other enriched Hallmark pathways from this analysis are shown in Supplemental Table 8.

To assess expression of STAT target proteins across MODS and patients with IEI, we performed unsupervised hierarchical clustering based on normalized protein expression of 31 proteins from the KEGG JAK/STAT gene set measured in our cohort. We assessed group concordance at the first bifurcation of the dendrogram, which separated the cohort into 2 clusters. As shown in Figure 5C, STAT1 GOF and STAT3 GOF patients colocalize with patients in Group C (*P* < 0.0001) while STAT1 DN and STAT3 DN patients colocalize with patients in Group A (*P* < 0.0001). This protein-level analysis also highlights the biologic heterogeneity within our severity-defined subgroups and the tested IEIs.

Having demonstrated enrichment of STAT target proteins in plasma from patients with MODS in Group C, patients with STAT1 GOF, and patients with STAT3 GOF, we next used GSVA to assess patient-level enrichment of 5 inflammatory pathways in patients with MODS and IEI. Figure 5D shows a clustered heatmap of module enrichment scores for each patient in the dataset. We noted increased IL-6/JAK/STAT3 module enrichment scores in all patients with STAT3 GOF and some with STAT1 GOF, while patients with STAT1 DN and STAT3 DN had IL-6/JAK/STAT3 module enrichment scores equivalent to participants in the HC group. Among patients with MODS, Groups B and C both showed elevated IL-6/JAK/STAT3 signaling compared with Group A (*P* = 0.018 and *P* = 0.007 respectively, Figure 5E). There was no significant difference between STAT3 GOF and patients in Group C, and many patients had enrichment scores that exceeded the median score of patients with STAT3 GOF. Collectively, these findings support the conclusion that patients in Group C exhibit sustained, high-magnitude IL-6/JAK/STAT3 pathway activation that mirrors, and, in some cases, exceeds, that observed in individuals with monogenic STAT3 gain-of-function mutations.

Finally, we examined the temporal dynamics of STAT3 pathway activation by comparing longitudinal IL-6/JAK/STAT3 module enrichment scores among the subset of 26 patients with greater than or equal to 3 serial plasma samples. Patients in Group C exhibited the highest enrichment scores at MODS onset, and these remained persistently elevated throughout the first 2 weeks of illness (Figure 5F). In contrast, enrichment scores in patients in Group B declined gradually over time, while scores in patients in Group A showed a rapid and sustained decrease. Notably, increases in IL-6/JAK/STAT3 signaling over time were rare: only 2 patients in Group C demonstrated a late rise in enrichment scores, with low initial activation followed by marked elevation approximately 1 week after MODS onset. These findings indicate that STAT3 hyperactivation in Group C is both an early and durable feature of disease, supporting its role as a driver of immune dysregulation rather than a transient host response.

### STAT1 and STAT3 signaling are associated with T cell immunometabolic dysregulation in MODS.

Because Group C represents the most severe phenotype in our multi-omic profiling assays, we focused our subsequent analyses on this subgroup to dissect the cellular mechanisms underlying STAT pathway hyperactivation in patients with MODS. Having demonstrated STAT3 pathway signaling in Group C patients at levels comparable to or exceeding those seen in patients with known STAT3 GOF, we next hypothesized that plasma STAT3 hyperactivation would result in immunometabolic dysregulation within lymphocytes from patients in Group C. To test this hypothesis, we performed single-cell RNA sequencing (scRNA-seq) using cryopreserved PBMCs obtained at MODS onset for patients in Group C (*n* = 9) and pediatric participants in the HC group (*n* = 3). Plasma IL-6/JAK/STAT3 module enrichment scores were significantly different between the 9 patients with MODS and 3 HC participants (0.331 versus –0.494, *P* < 0.0001) selected for scRNA-seq.

**Figure 6:**
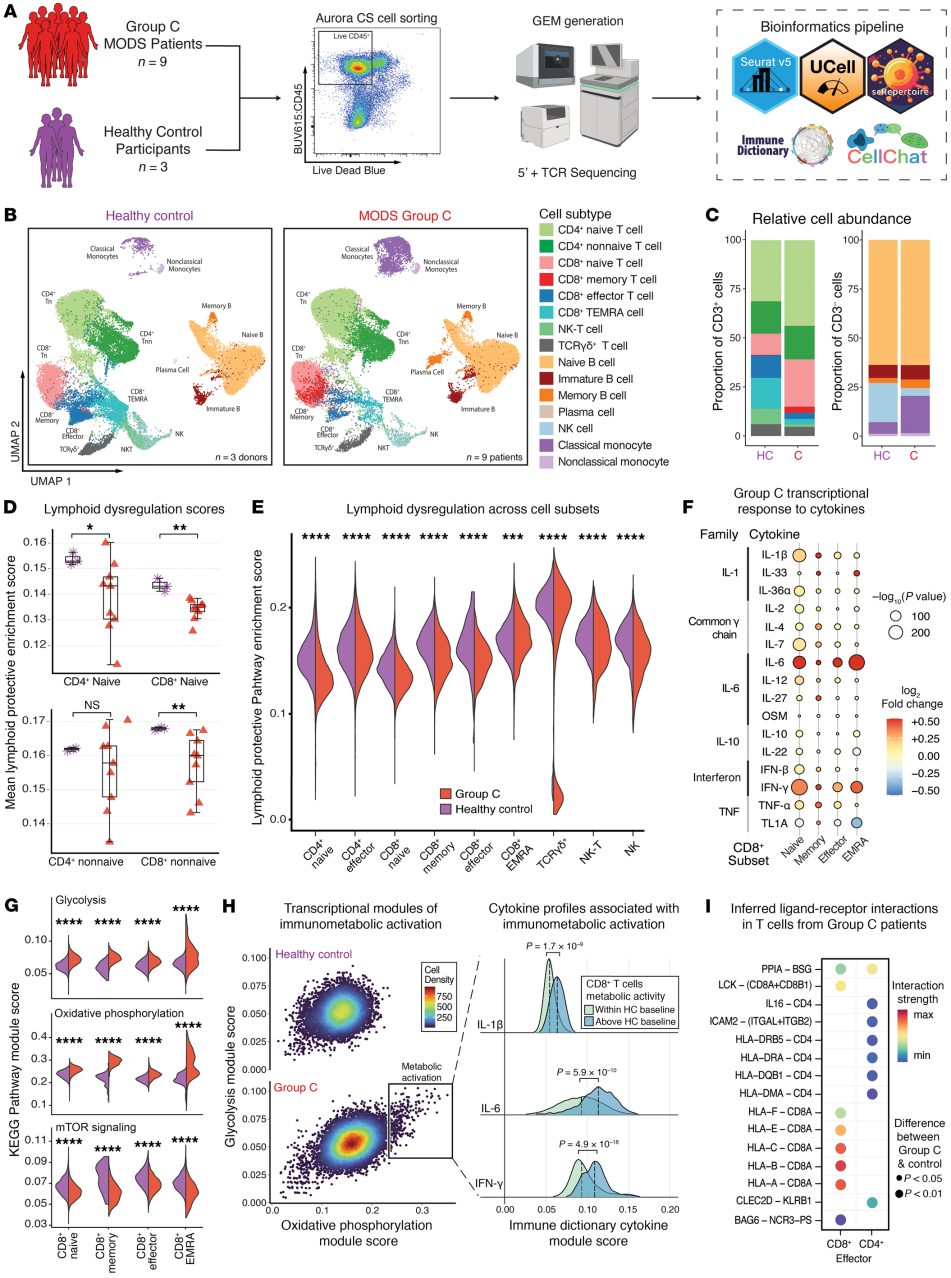
STAT1 and STAT3 signaling promote T cell immunometabolic dysregulation. (**A**) scRNA-seq experimental design (Group C, *n* = 9; HC, *n* = 3). (**B**) UMAP projection of lymphoid and myeloid lineages. (**C**) Proportional abundance of CD3^+^ and CD3^–^ subsets by group. (**D**) Pseudobulk enrichment scores for the lymphoid protective gene module. Box-and-whisker plots show median and IQR (whiskers 1.5 × IQR). (**E**) Single-cell lymphoid protective module enrichment scores across lymphoid subsets. (**F**) Cytokine-response signatures inferred using the Immune Dictionary framework. Bubble color represents enrichment magnitude in Group C relative to HC; size indicates significance. (**G**) Glycolysis, oxidative phosphorylation, and mTOR signaling enrichment in CD8^+^ T cells. (**H**) Bivariate metabolic enrichment scores identify metabolically activated CD8^+^ T cells in Group C, and cytokine-response scores in activated versus nonactivated cells (right; mixed-effects model). (**I**) Ligand-receptor interaction analysis in CD4^+^ and CD8^+^ T cells. Color indicates interaction strength; size indicates significance. Comparisons by Wilcoxon rank-sum test unless otherwise noted, with Benjamini-Hochberg FDR adjustment: *P < 0.05, **P < 0.01, ***P < 10^-12^, ****P < 10^-16^.

A schematic of our experimental approach is shown in Figure 6A. We sorted live CD45^+^ PBMCs and then profiled 10,000 cells per patient using the 10X Genomics 5′ single cell gene expression platform. Libraries were sequenced to a depth of approximately 30,000 reads per cell on a Novaseq S2 (Illumina). Transcripts were aligned to the GRCh38 reference genome using Cell Ranger v8.0. After quality control using SoupX (55) and DoubletFinder (56) and integration using RPCA via Seurat v5 (57), cell identities were inferred using ScType (58) and refined using Azimuth (59) and T cell phenotypes from Giles et al. (60). Using this approach, we identified 15 immune cell populations by transcriptional profile for downstream analysis. We applied UMAP (61) dimensionality reduction and subsampled 30,000 cells from each group (HC and Group C) for visualization of immunophenotypic differences (Figure 6B). Differences in cell populations between participants in the HC group and Group C are quantified in stacked bar plots (Figure 6C) and largely mirror the differences in lineages seen in our flow cytometry analysis, redemonstrating the altered immune cell composition in patients in Group C.

Building on our flow-cytometry evidence of T cell depletion and activation and our plasma evidence of STAT3 hyperactivation, we first tested whether a validated transcriptomic marker of dysfunctional lymphoid immunity was similarly perturbed in patients in Group C. We applied a transcriptional framework developed by the SUBSPACE consortium (62), which identified a “lymphoid protective” gene set derived from adult patients with sepsis. Lower scores on this module denote a “lymphoid detrimental” transcriptional program associated with T cell dysfunction and poor outcomes in adult sepsis. We calculated “lymphoid protective” module enrichment scores at the single-cell level using UCell (63) and generated pseudobulk profiles for statistical analysis to control Type 1 error (64). Consistent with our protein-level findings, patients in Group C exhibited significantly lower lymphoid protective scores compared with participants in the HC group in CD4^+^ naive, CD8^+^ naive, and CD8^+^ nonnaive populations (Figure 6D).

To broaden our assessment of transcriptional dysregulation, we extended our analysis to include per-cell lymphoid protective module enrichment scores across 9 major lymphoid subsets (Figure 6E). Patients in Group C exhibited significantly lower module scores across all subsets, with the most pronounced deficits observed in the CD8^+^ T cell compartment. Together, these results indicate that lymphoid dysregulation in Group C is not restricted to numerical depletion but reflects widespread changes to immune regulatory gene programs necessary for effective immune surveillance.

To infer which cytokines may drive immune dysregulation in Group C lymphocytes, we leveraged the Immune Dictionary framework (65), which enables cell-type–specific identification of cytokine responses based on downstream gene expression patterns. As shown in Figure 6F, patients in Group C exhibited significantly increased transcriptional responses to multiple proinflammatory cytokines compared with participants in the HC group, including IL-6 (which activates STAT1 and STAT3) and IFN-γ (which predominantly activates STAT1). These findings indicate that sustained exposure to a proinflammatory cytokine milieu, particularly IL-6 and IFN-γ, may drive the transcriptional reprogramming observed in lymphocytes from patients in Group C.

Because corticosteroids modulate cytokine signaling, we performed a sensitivity analysis to determine if these transcriptional signatures were affected by treatment allocation. We compared lineage-specific IL-6/JAK/STAT3 module enrichment scores between patients in Group C who received corticosteroids (*n* = 4) and those who did not (*n* = 5). Using Cliff’s Delta (*δ*) to quantify effect size, corticosteroid exposure was associated with lower IL-6/JAK/STAT3 module enrichment scores in classical monocytes (*δ* = 0.42, medium effect), whereas effect sizes in T cell subsets were small or negligible (e.g., CD8^+^ central memory T cells, *δ* = –0.23, small effect) (Supplemental Table 9). These exploratory data, while limited by sample size, do not support corticosteroid-mediated suppression as an explanation for the lymphoid transcriptional phenotype observed in Group C.

To understand the effects of sepsis on T cell immunometabolism, we calculated KEGG pathway module enrichment scores for glycolysis, oxidative phosphorylation, and mTOR signaling pathways using UCell (63) as detailed in Methods. As shown in Figure 6G, CD8^+^ T cells from patients in Group C exhibited increased transcriptional activity in glycolysis and oxidative phosphorylation pathways compared to HC participants across all differentiation states. Conversely, mTOR signaling was suppressed across CD8^+^ T cell subsets from Group C, a pattern suggesting cytokine-driven repression of the mTOR pathway, which may affect T cell function (66).

Given that IL-6 and IFN-γ exert complex effects on immunometabolic state and mTOR signaling, we next assessed whether exposure to these signals explained the metabolic phenotype observed in Figure 6G. To test whether specific cytokines underpin this bioenergetic shift, we modeled the joint scores of single-cell Hallmark glycolysis and oxidative phosphorylation modules (Figure 6H). This revealed a distinct subset of Group C CD8^+^ T cells with increased glycolysis and oxidative phosphorylation transcriptional signatures (labeled as “metabolic activation”). In multivariable mixed-effects logistic regression analysis, enrichment scores for IL-6, IFN-γ, and IL-1β transcriptional responses are significantly increased in metabolically activated CD8^+^ T cells compared with cells within the HC baseline range (all *P* < 0.001), as shown in the ridge plots in Figure 6H.

Finally, to further characterize the amplified activation state of CD8^+^ T cells in patients in Group C, we applied CellChat (67) to the scRNA-seq dataset to identify predicted cell-to-cell communication events via curated ligand-receptor pairs and coreceptor interactions that modulate signaling. As shown in Figure 6I, patients in Group C exhibited significantly stronger inferred signaling interactions between monocyte-expressed HLA class I molecules and the *CD8A* coreceptor on CD8^+^ T cells, compared with participants in the healthy control group (*P* < 0.01). Increased inferred HLA-CD8A interactions are consistent with observed CD8^+^ T cell activation in Group C and align with our transcriptional and metabolic findings, although this modeling does not establish altered antigen presentation or a causal role of myeloid-lymphoid crosstalk in sepsis-associated immune dysregulation.

### T cell activation in MODS occurs without antigen-specific clonal expansion.

Having demonstrated lymphoid dysregulation and cytokine-linked metabolic reprogramming in patients in Group C, we next investigated whether these immunometabolic changes reflected antigen-specific immune responses or nonspecific bystander activation. We analyzed T cell receptor (TCR) repertoires captured alongside our scRNA-seq data using 5′ tag-based single-cell V(D)J sequencing (10X Genomics). VDJ libraries were sequenced to a depth of approximately 10,000 reads per cell on a Novaseq S2 (Illumina) and processed with Cell Ranger v8.0, aligning to the GRCh38 reference genome. TCR data were integrated with scRNA-seq analyses in Seurat using the scRepertoire package (68). We used this dataset to test the hypothesis that T cell activation in patients in Group C represented antigen-independent “bystander” activation as opposed to antigen-specific activation in response to a pathogen.

**Figure 7:**
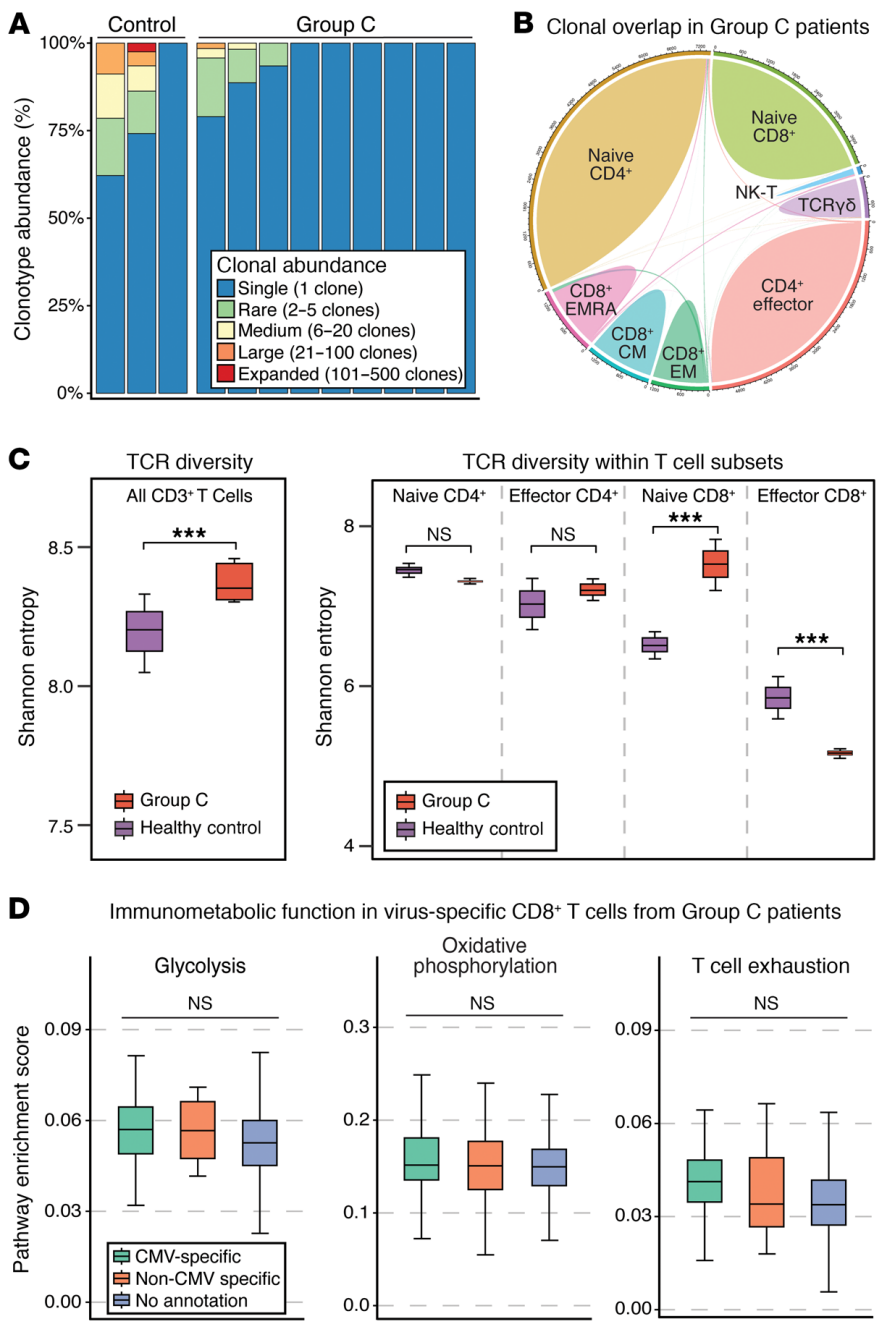
Polyclonal, antigen-independent T cell activation in patients in Group C. (**A**) Clonal abundance of CD3^+^ T cells from scTCR-seq data, showing predominantly single- or rare-frequency clonotypes. (**B**) Chord diagram of TCR clonal sharing across T cell subsets in patients in Group C. (**C**) Shannon diversity of TCR repertoires. Comparisons by Wilcoxon rank-sum test with Benjamini-Hochberg adjustment: *P < 0.05, **P < 0.01, ***P < 0.001, ****P < 0.0001. (**D**) Immunometabolic pathway enrichment of CMV-specific versus nonspecific CD8^+^ T cells. Comparisons by Kruskal-Wallis test with Benjamini-Hochberg adjustment. Box-and-whisker plots show median and IQR (whiskers 1.5 × IQR).

To computationally assess antigen specificity, we first quantified clonal expansion across the TCR repertoire at the individual patient level. In contrast with participants in the HC cohort who exhibited expected homeostatic clonal expansion, including the presence of hyperexpanded clones, patients in Group C were characterized by a marked absence of expanded clones (Figure 7A). No single clone in Group C patients occupied more than 1% of the repertoire, and most clones were present as single, unique sequences. This lack of clonal expansion argues against antigen-driven clonal expansion as the dominant mechanism of T cell activation in patients in Group C.

We next assessed paired TCR diversity using the Shannon index, which accounts for both the richness (number of unique clonotypes) and evenness (relative abundance) of clonotypes. As shown in Figure 7B, patients in Group C exhibited significantly higher Shannon diversity compared with participants who were healthy controls (*P* < 0.001), consistent with broad, nonspecific T cell activation. Subset-level analysis revealed that TCR diversity remained comparable with controls in naive and effector CD4^+^ T cells, but was increased in naive CD8^+^ T cells and decreased in effector CD8^+^ T cells (both *P* < 0.001), suggesting that alterations to the TCR repertoire are specific to CD8^+^ lineages.

To determine whether CD8^+^ T cell activation reflected antigen-specific clonal expansion, we visualized clonotype overlap across T cell subsets. As shown in Figure 7C, patients in Group C exhibited minimal overlap between naive, central memory, effector memory, and EMRA CD8^+^ T cell populations, again providing evidence against substantial expansion of antigen-specific clones and supporting a model of bystander activation.

Finally, we investigated whether immunometabolic pathway enrichment differed by antigen specificity predicted in silico using TReX (69) based on reference TCR sequences from VDJdb (70), McPAS-TCR (71), IEDB (72), and PIRD (73). DNA viremia is associated with immune dysregulation in pediatric sepsis (74), and we hypothesized that virus-specific CD8^+^ T cells could account for the immunometabolic changes noted in patients in Group C. Using cytomegalovirus (CMV) as a model of chronic viral antigen exposure, we stratified CD8^+^ T cells by CMV-specific, non-CMV–specific, and unannotated TCRs and compared glycolysis, oxidative phosphorylation, and T cell exhaustion gene set enrichment scores across these groups using UCell. No significant differences were observed across these groups (Figure 7D), suggesting that virus-specific CD8^+^ T cells do not drive the immunometabolic changes observed in Group C.

Taken together, these findings suggest that T cell activation and immunometabolic dysregulation in Group C patients occur independently of antigen-specific clonal expansion, which is typically associated with pathogen-specific responses. These data support a model of cytokine-driven, bystander T cell activation in the setting of STAT3 and STAT1 hyperactivation, rather than a conventional antigen-specific immune response.

### IL-6 and IFN-γ signaling drive aberrant STAT pathway activation and T cell dysfunction in MODS.

Building on our single-cell transcriptional analyses, which demonstrated antigen-independent T cell activation and marked enrichment of IL-6 and IFN-γ response modules in CD8^+^ T cells from patients in Group C, we next sought to test whether these cytokines drive functional changes in signaling at the protein level. To do so, we employed an optimized 13-marker T cell phosphoflow cytometry panel (Supplemental Table 10) which allows for identification of T cells and includes phospho STAT (pSTAT) antibodies that recognize pSTAT1, pSTAT3, pSTAT5, and total STAT proteins.

**Figure 8:**
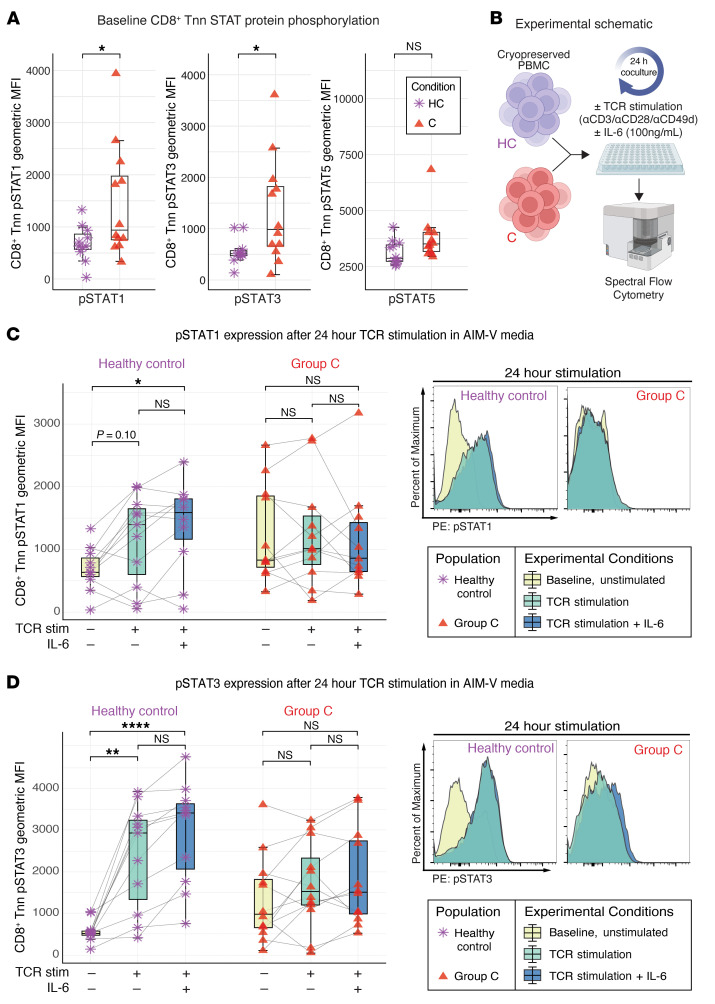
IL-6 and IFN-γ signaling drive aberrant STAT pathway activation and T cell dysfunction. (**A**) Baseline pSTAT1 and pSTAT3 expression in nonnaive CD8^+^ T cells. Comparisons by Wilcoxon rank-sum test with Benjamini-Hochberg adjustment. (**B**) Schematic of the ex vivo TCR stimulation. (**C**) pSTAT1 and (**D**) pSTAT3 upregulation in response to TCR stimulation ± IL-6, with representative histograms. Group C nonnaive CD8^+^ T cells exhibit blunted phosphorylation responses compared with HC. Box-and-whisker plots show median and IQR (whiskers 1.5 × IQR). Unless otherwise noted, comparisons by Kruskal-Wallis test followed by Dunn’s post hoc test with Benjamini-Hochberg adjustment: *P < 0.05, **P < 0.01, ***P < 0.001, ****P < 0.0001.

We first examined basal phosphorylation of STAT1 and STAT3, key transcription factors downstream of IFN-γ and IL-6 signaling, respectively, though their use as heterodimers and homodimers downstream of cytokine receptors is complex and includes many other cytokines. Compared with HC, patients in Group C at baseline exhibited higher expression of pSTAT1 (*P* = 0.041) and pSTAT3 (*P* = 0.026) in nonnaive CD8^+^ T cells (Figure 8A). This finding is consistent with the demonstrated elevated plasma cytokine levels, as well as our transcriptional cytokine response analysis, suggesting that IL-6 and IFN-γ exert a concurrent effect on CD8^+^ T cells in the patients in Group C.

Given the elevated baseline STAT signaling in Group C, we next assessed how this cytokine exposure affects CD8^+^ T cell responsiveness to receptor-mediated stimulation. As illustrated in Figure 8B, PBMCs from patients in Group C were thawed and cultured for 24 hours with plate-bound αCD3 and soluble αCD28/αCD49d antibodies. Using this platform, we compared the ability of Group C CD8^+^ T cells to respond to TCR stimulation alone or in combination with IL-6.

As shown in Figure 8C, HC cells demonstrate robust induction of pSTAT1 following stimulation with αCD3/αCD28/αCD49d and IL-6 (*P* = 0.033). In contrast, Group C cells failed to increase pSTAT1 expression in response to TCR stimulation with or without IL-6 (*P* = 0.92). Representative pSTAT1 histograms highlight the lack of response to stimulation in CD8^+^ T cells from Group C patients. Analysis of pSTAT3 signaling in Figure 8D demonstrated similar findings. HC cells upregulated pSTAT3 following αCD3/αCD28/αCD49d stimulation (*P* = 0.005), and IL-6 further increased pSTAT3 levels (*P* < 0.001). In contrast, Group C cells failed to increase pSTAT3 expression in response to TCR stimulation with or without IL-6 (*P* = 0.46). Representative pSTAT3 histograms again highlight the lack of response to stimulation in CD8^+^ T cells from patients in Group C.

Together, these findings support the hypothesis that sustained cellular signaling downstream of IL-6 and IFN-γ in patients in Group C may drive chronic activation of the STAT1 and STAT3 signaling pathways, leading to a state of functional desensitization to further activation of those pathways in CD8^+^ T cells. While plasma IFN-γ levels were variable (Figure 4E), the presence of robust IL-6 and IFN-γ transcriptional signatures (Figure 6F) and elevated basal pSTAT1 and pSTAT3 levels (Figure 8A) suggests that these cells have integrated inflammatory signals that result in a failure to respond appropriately to subsequent TCR-mediated stimulation, indicating a state of refractory responses to cytokine signals. These data implicate STAT1 and STAT3 signaling as central mediators of CD8^+^ T cell dysregulation in Group C, potentially defining an endotype that drives dysregulation in this subgroup. Our multi-omic findings therefore provide a mechanistic link between systemic inflammation, transcriptional rewiring, and impaired adaptive immunity, which may be therapeutically tractable and requires further study.

## Discussion

In a prospective cohort of children admitted to a quaternary PICU with MODS from diverse etiologies, we have defined a distinct immunologic endotype characterized by persistent STAT1/STAT3 hyperactivation and T cell immunometabolic dysfunction in a subset of patients with the highest severity of illness and worst prognosis. These patients can be identified by a 24-protein signature at MODS onset that is agnostic to underlying etiology, suggesting a convergent immunopathologic trajectory across diverse infectious and noninfectious insults. Because this signature relies solely on soluble biomarkers, it may be amenable to future development as a rapid clinical assay, enabling targeted studies of endotype-guided precision immunomodulation.

Multimodal immune profiling revealed that Group C is characterized by elevated IL-6 and a robust cellular response to IFN-γ, resulting in persistent activation of the STAT1 and STAT3 signaling pathways. Single-cell RNA-seq revealed broad suppression of lymphoid protective gene programs (62) and enhanced transcriptional signatures of T cell exhaustion, metabolic stress, and cytokine-driven activation, particularly within CD8^+^ T cell subsets. Despite high expression of activation markers by flow cytometry and scRNA-seq, our TCR repertoire analysis revealed no evidence of clonal expansion, consistent with antigen-independent, bystander T cell activation. Functionally, CD8^+^ T cells from Group C patients exhibited increased baseline STAT1 and STAT3 phosphorylation, yet failed to respond to TCR-mediated stimulation. These findings indicate a state of cytokine-induced desensitization or functional refractoriness in CD8^+^ T cells from patients in Group C.

Our highly granular approach to immune profiling yields new insights into the pathobiology of pediatric sepsis and MODS, identifying a potentially targetable mechanism of immunopathology — STAT1/STAT3 hyperactivation associated with transcriptional evidence of T cell immunometabolic dysregulation in critically ill children with and without sepsis. To our surprise, we identified features of immune dysregulation in many patients with MODS who did not have suspected infection, and all 3 proteomic subgroups include patients with and without sepsis. Our findings redemonstrate the association between T cell dysfunction, mitochondrial dysfunction, and clinical outcomes in pediatric sepsis (19), and suggest a potential molecular mechanism for sepsis-associated T cell immunometabolic dysregulation. As other investigators have shown, the highest severity patients in our cohort have innate (15, 17) and adaptive (16) immune dysfunction and mitochondrial dysfunction (19, 20). Recent human and murine data have identified a role for STAT3 signaling in the pathogenesis of sepsis (51–53), and our results concordantly demonstrate associations between STAT3 signaling, clinical outcomes, and features of immune dysregulation implicated in the pathobiology of pediatric sepsis.

Aberrant STAT1 and STAT3 signaling in this endotype are potentially targetable through approved anti-cytokine antibodies, JAK inhibitors, and experimental STAT inhibitors (75), and, thus, represent viable future targets for precision medicine investigations in pediatric patients with sepsis. Supporting this hypothesis, during the COVID-19 pandemic, some patients requiring supplemental oxygen were noted to benefit from a combination of dexamethasone and a JAK inhibitor (76), and the use of JAK inhibitors baricitinib and tofacitinib were associated with reduced mortality (77), faster recovery (78), and lower rates of disease progression (79) in multiple adult trials across levels of illness severity. Several available JAK inhibitor agents have good bioavailability, rapid onset of action, and short half lives, features that are favorable for use in the critical care setting. While the safety and efficacy of baricitinib in critical COVID-19 provide a compelling precedent, further mechanistic studies are required to confirm STAT1/STAT3 hyperactivation as a causal contributor to immune cell dysregulation and organ failure in pediatric sepsis before JAK inhibition should be pursued in clinical trials for this population.

The longitudinal analysis of protein and pathway expression in this study provides evidence that immune dysregulation present at MODS onset persists for more than a week in many patients. Persistent immune dysregulation is a suitable candidate for consideration of a trial of precision therapeutics in which patients could be identified at MODS onset and then assigned to a treatment arm based on biomarker-based prognostic and predictive enrichment strategies. Equally relevant, prognostic subgroups defined at MODS onset demonstrated relatively stable proinflammatory pathway enrichment scores over time, suggesting that subgroup membership is patient- or episode-specific and minimally impacted by the time from MODS onset. Future studies should test longitudinal endotype stability and validate these subgroups in independent pediatric sepsis cohorts.

By incorporating comparisons with rare, monogenic IEI, we can contextualize the complex biology of sepsis against natural human models of chronic pathway-level dysregulation within key signaling cascades. Historically identified in children with severe presentations of specific infectious diseases (80), and more recently in patients with immune dysregulation (81), IEI are known to be enriched in pediatric patients with COVID-19 (82, 83), influenza (84), and sepsis (85). Genotype-phenotype associations between monogenic IEI and disease susceptibility may offer insights into the pathobiology of illness, which subsequently inform precision therapeutics (86). More recently, it has become clear that many IEI are characterized by immune dysregulation (81). By comparing patients with MODS with STAT1 and STAT3 gain-of-function and loss-of-function disorders (87), we leverage human models of chronic pathway perturbation to interpret complex immune states, learning human biology from these rare disorders. This analysis reveals that the most severe MODS subgroup exhibits acute STAT1/STAT3 hyperactivation, surpassing that produced by genetic activation of the STAT1 and STAT3 pathways.

The cellular correlates of immune dysfunction observed in this study — defined by persistent STAT1 and STAT3 hyperactivation, altered metabolic programming, and impaired responsiveness to TCR stimulation — were reproducible across platforms, including spectral flow cytometry and scRNA-seq. We observed a potential divergence between systemic protein profiles and cell-specific metabolic signatures. While plasma proteomics indicated broad suppression of the mTOR signaling pathway in patients in Group C, CD8^+^ T cells simultaneously exhibited transcriptional signatures of enhanced glycolysis and oxidative phosphorylation. This finding suggests that, while the systemic environment reflects a state of metabolic suppression, individual immune cells maintain a distinct, cytokine-driven metabolic activation phenotype. These phenotypic and functional features may serve as candidate functional biomarkers to act as surrogate endpoints in early-phase interventional studies. For example, ex vivo pSTAT signaling assays or CXCL9 levels could potentially be used to evaluate pharmacodynamic responses to JAK inhibition or cytokine blockade, while immune cell metabolic profiling could potentially track immunometabolic recovery following treatment. Ultimately, these findings define a path toward biomarker-guided, mechanism-informed interventions for critically ill children and establish a new translational roadmap for targeting immune dysregulation in pediatric sepsis.

Building on this mechanistic framework, we employed penalized regression to identify a prognostic protein signature detectable at MODS onset and demonstrated excellent discrimination using linear discriminant analysis. Elastic net regularization is a common approach applied to feature selection in high-dimensional data, which generates a high-performing sparse model with good prediction accuracy (40) and has been employed with similar results in adult sepsis studies (34). Prior to potential use for prognostication or selection of targeted therapeutics, the proposed parsimonious 24-protein MODS severity model requires refinement and prospective validation. It will also benefit from validation across multiple proteomics platforms, as aptamer-based and antibody-based approaches are susceptible to both technical and genetic variation among samples (88).

Limitations of our study include an a priori sample size that was only powered to resolve 3 proteomic subgroups within a heterogeneous syndrome. This reflects a pragmatic approach to study design and does not preclude the existence of other subgroups and/or endotypes. Additionally, our flow cytometry and transcriptomics experiments relied on cryopreserved PBMC samples. While this approach was necessary to minimize batch effect associated with longitudinal sample collection, it is possible that fragile cell populations may have been impacted by a single freeze/thaw cycle. The use of PBMCs precludes cellular analysis of granulocyte populations, particularly neutrophils, which are major targets of IL-6 signaling in sepsis. Differences in corticosteroid sensitivity between myeloid and lymphoid lineages in our scRNA-seq analysis suggest that granulocytes may exhibit distinct STAT3 signaling dynamics that are not captured in our PBMC-derived dataset, which lacks neutrophils. Furthermore, characterization of T cell immunometabolic dysregulation relies on transcriptional proxies of metabolic pathway activity, which suggest altered metabolic states but do not directly measure metabolic flux. Future validation studies using functional assays, such as Seahorse extracellular flux analysis or stable-isotope tracing, will be needed to confirm altered glycolytic and mitochondrial metabolism. Finally, we analyzed circulating blood cells and plasma proteins, but the functional similarities between circulating immune cells and tissue-resident immune cells are not known in this hyperinflammatory setting.

Collectively, our longitudinal multi-omics analysis defines a high-risk endotype in which aberrant signaling via the IL-6 and IFN-γ axes leads to persistent STAT1 and STAT3 activation, culminating in immune dysregulation and impaired T cell responsiveness. Concordant results from proteomic, cytometric, and transcriptomic assays underscore the biological coherence of this endotype, which is associated with poor prognosis and can be defined at the onset of MODS using a parsimonious plasma proteomic signature. Taken together, these findings provide a model for the discovery of potential biomarker-guided, mechanism-informed precision therapies in critically ill children, identify the JAK/STAT axis as a candidate target, and support the broader exploration of functional immune profiling as a tool for tailoring immunomodulation in pediatric critical care.

## Methods

### Sex as a biological variable.

Sex was recorded for all participants and included as a covariate in all multivariable models to account for potential sex-related differences in immune responses. Both male and female patients were represented across all subgroups; analysis was not stratified by sex, as the study was not powered to detect sex-specific effects.

### MODS patient samples.

We enrolled patients with MODS into a prospective observational cohort study in the Pediatric and Cardiac Intensive Care Units at Children’s Hospital of Philadelphia. Eligible patients were >40 weeks post-conceptual age and <18 years with new dysfunction of ≥2 organs defined by modified Proulx criteria (Supplemental Table 11) (11). Full inclusion/exclusion criteria are provided in Supplemental Clinical Methods. Patients were enrolled between June 2020 and December 2022 until the prespecified enrollment target of 88 patients was reached, which provided 90% power to detect moderate differences among three subgroups (Cohen’s d=0.5), based on pilot flow cytometry and proteomics data. This study co-enrolled with the multicenter PediAtric ReseArch of Drugs, Immunoparalysis and Genetics during MODS (PARADIGM) study, with shared eligibility criteria and clinical metadata but independent biospecimen processing and analysis. Longitudinal whole blood biospecimens were collected starting within 48 hours of MODS onset and twice weekly thereafter until death, resolution of organ dysfunction, or a maximum of six samples. Peripheral blood mononuclear cells (PBMCs) and platelet-poor plasma were isolated by density gradient centrifugation and stored at –80°C or in liquid nitrogen as detailed in Supplemental Laboratory Methods.

### Clinical metadata and outcomes.

Clinical metadata were abstracted by a nurse research coordinator into REDCap (89) for the parent PARADIGM study and verified through manual and automated queries. MODS etiology was classified as sepsis, trauma, cardiopulmonary bypass, or non-infectious based on clinical documentation and microbiologic testing. Immunocompromised status was defined by active malignancy, prior hematopoietic cell transplantation, or primary immunodeficiency. Daily PELOD-2 scores were calculated from MODS onset through day 28, and cumulative PELOD-2 score served as the primary outcome. Exposure and outcomes measures are detailed in Supplemental Clinical Methods.

### Control and comparator cohorts.

Cryopreserved samples from children without immunologic disease served as a healthy control group (HC, *n* = 25). Age range and sex of healthy control participants are shown in Supplemental Table 12. Plasma samples from inborn error of immunity (IEI) patients with STAT1 gain-of-function (*n* = 9), STAT1 dominant-negative (*n* = 1), STAT3 gain-of-function (*n* = 5), and STAT3 dominant-negative (*n* = 3) mutations were obtained from collaborators and served as comparators for pathway-specific dysregulation phenotypes.

### Plasma proteomics.

Heparin plasma samples were analyzed using the Explore 1536 proximity extension assay (36) platform (Olink Proteomics, Uppsala, Sweden). Protein concentrations were reported in log_2_-transformed Normalized Protein eXpression (NPX) units. We analyzed 229 samples from 131 patients across three experiments using 8 shared samples for normalization. 1448 proteins met quality control thresholds in all three experiments and were included in downstream analyses (Supplemental Table 13). IEI samples were analyzed separately using the Olink Explore 384 Inflammation panel (Supplemental Table 7).

### Spectral flow cytometry staining and acquisition.

Cryopreserved PBMCs were stained with 2 high-dimensional spectral flow cytometry panels designed to characterize broad immune phenotypes and intracellular phosphoprotein signaling. For phosphoflow cytometry, cells were stimulated *ex vivo* with αCD3/αCD28/αCD49d antibodies with or without IL-6 prior to fixation and permeabilization. Data were acquired via Cytek Aurora spectral flow cytometer (Cytek Biosciences, Fremont, CA). We analyzed 303 samples from 113 patients for immune phenotyping, and 69 samples from 23 patients for phosphoflow cytometry. Full antibody panels and staining protocols are available in Supplemental Table 2, Supplemental Table 10, and Supplemental Laboratory Methods.

### scRNA-seq and TCR-seq.

Paired single-cell transcriptomic and T cell receptor (TCR) sequencing was performed on live CD45+ cells from a subset of MODS Group C (*n* = 9) and HC participants (*n* = 3) using the 10X Genomics Chromium Next GEM Single Cell 5′ Kit v2 platform (10X Genomics, Pleasanton, CA). Samples were selected at random from Group C and HC cohorts to avoid bias. Libraries were sequenced on an Illumina S2 flow cell (Illumina, San Diego, CA) and aligned to the GRCh38 reference genome using the Cell Ranger v8.0 pipeline (10X Genomics). Details of flow cytometric cell sorting and library preparation are provided in Supplemental Laboratory Methods.

### Statistics.

Statistical analyses integrated proteomic, cytometric, and transcriptomic datasets using established mixed-effects modeling, consensus clustering, and multivariate nonparametric frameworks. Comparisons across groups used the Kruskal-Wallis test followed by Dunn’s post hoc test with Benjamini-Hochberg adjustment for multiple comparisons. Severity-associated proteins were identified using linear mixed-effects models for each analyte, adjusting for age and sex as fixed effects and day from MODS onset as a random effect. Patient subgroups were defined by consensus clustering, with optimal *k* determined using the proportion of ambiguous clustering (PAC) metric, and confirmed by reference-based Monte Carlo bootstrapping, elbow method, and gap statistic, yielding *k* = 3. Survival analyses employed proportional subdistribution hazards regression (Fine-Gray) to account for competing risks. An ordinal elastic net model to derive a parsimonious classifier for patient subgroups was trained using a semiparallel elementwise link multinomial-ordinal (ELMO) regression framework with elastic-net shrinkage to minimize the Gini index, and evaluated using PCA, mixed-effects postestimation, and the polytomous discrimination index.

Spectral flow cytometry data were arcsinh transformed (42), quality controlled with flowAI (43), and clustered using FlowSOM (44) to yield 14 immune populations, with cluster similarity assessed by marker expression and tSNE-CUDA (45) and validated by manual gating. Pathway enrichment was evaluated by Ingenuity Pathway Analysis, bulk GSEA, and patient-level GSVA using MSigDB Hallmark and KEGG gene sets (48). Single-cell RNA-seq data were analyzed using Seurat (57) with SCTransform and RPCA integration. Cell identities were assigned using scType (58) and manual curation based on gene lists from Azimuth (59) and published T cell phenotypes (60). Cell-cell communication was inferred using CellChat (67). Cytokine-specific transcriptional responses were quantified using the Immune Dictionary (65). TCR diversity and clonality were analyzed using scRepertoire (68). TCR clonotypes were annotated using TReX (69). Computational analysis was completed using R 4.5.0 and Bioconductor 3.20. Flow cytometry data were processed using FlowJo 10.9 and Omiq.ai. Details are available in Supplemental Computational Methods.

### Study approval.

Written informed consent (and assent, when appropriate) was obtained from all participants or their legal guardians in accordance with IRB-approved protocols. Patients with MODS were enrolled under CHOP IRB #19-017032. HC participants were enrolled under CHOP IRB #18-15920. Participants with IEI were enrolled under local IRB protocols and shared through collaborative research agreements under IRB #18-015920.

### Data availability.

Code and deidentified study data are available at https://github.com/Lindell-Lab/STAT-Hyperactivation-in-Sepsis, branch main, commit ID bfaa21f. MINSEQE-compliant transcriptional data are deposited under GEO accession number GSE303333. Data values used to generate each figure are provided in the Supporting Data Values file.

## Author contributions

RBL conceived and led the study, with mentorship from NJM and SEH. JCF, NY, EMB, DTT, SLW, MWH, DMT, RF, and EJW served as scientific advisors. Funding was secured by RBL, JCF, EMB, DTT, NJM, and SEH. Experiments were performed by RBL, SUS, SAS, JSCD, PEC, AAM, and CAH. Bioinformatics analyses were conducted by RBL, AB, MSK, and HF. Patient enrollment and biospecimen coordination were supported by STF, TA, and RT. IEI biospecimens were provided by LRFS, AFF, JREB, SMH, and JWL. RBL drafted the initial manuscript. All authors reviewed and approved the final manuscript.

## Conflict of interest

RBL, NJM, and SEH are named as coinventors on a published PCT patent application filed by Children’s Hospital of Philadelphia and the University of Pennsylvania regarding the pediatric MODS molecular subgroups described in this study (WO/2025/245413).

## Funding support

This work is the result of NIH funding, in whole or in part, and is subject to the NIH Public Access Policy. Through acceptance of this federal funding, the NIH has been given a right to make the work publicly available in PubMed Central.

- NIH grants K12HD047349 (RBL), K23GM159013 (RBL), K08AI135091 (SEH), and R01HD095976 (MWH).
- Thrasher Research Fund (RBL).
- Burroughs Wellcome Fund (SEH).
- Immune Deficiency Foundation (SEH).
- Primary Immune Deficiency Treatment Consortium (SEH).
- Barbara Brodsky Foundation (DTT).
- CHOP Research Institute (RBL).

## Supplementary Material

Supplemental data

ICMJE disclosure forms

Supplemental tables 1, 4, 7 and 13

Supporting data values

## Version 1 06/09/2026

In-Press Preview

## Version 2 07/15/2026

Electronic publication

## References

[1] Singer M (2016). The third international consensus definitions for sepsis and septic shock (Sepsis-3). JAMA.

[2] Rudd KE (2020). Global, regional, and national sepsis incidence and mortality, 1990-2017: analysis for the Global Burden of Disease Study. Lancet.

[3] Weiss SL (2015). Global epidemiology of pediatric severe sepsis: the sepsis prevalence, outcomes, and therapies study. Am J Respir Crit Care Med.

[4] Zinter MS (2015). New insights into multicenter PICU mortality among pediatric hematopoietic stem cell transplant patients. Crit Care Med.

[5] Lindell RB (2017). High levels of morbidity and mortality among pediatric hematopoietic cell transplant recipients with severe sepsis: insights from the sepsis prevalence, outcomes, and therapies international point prevalence Study. Pediatr Crit Care Med.

[6] Lindell RB (2020). Risk of mortality in immunocompromised children with severe sepsis and septic shock. Crit Care Med.

[7] Weiss SL (2017). The epidemiology of hospital death following pediatric severe sepsis: when, why, and how children with sepsis die. Pediatr Crit Care Med.

[8] Lin JC (2017). New or progressive multiple organ dysfunction syndrome in pediatric severe sepsis: a sepsis phenotype with higher morbidity and mortality. Pediatr Crit Care Med.

[9] Leteurtre S (2013). PELOD-2: an update of the PEdiatric logistic organ dysfunction score. Crit Care Med.

[10] Schlapbach LJ (2022). Scoring systems for organ dysfunction and multiple organ dysfunction: The PODIUM Consensus Conference. Pediatrics.

[11] Proulx F (2009). The pediatric multiple organ dysfunction syndrome. Pediatr Crit Care Med.

[12] Carcillo JA (2017). Pathophysiology of pediatric multiple organ dysfunction syndrome. Pediatr Crit Care Med.

[13] Weiss SL (2022). Refining the pediatric multiple organ dysfunction syndrome. Pediatrics.

[14] Maslove DM (2022). Redefining critical illness. Nat Med.

[15] Hall MW (2011). Immunoparalysis and nosocomial infection in children with multiple organ dysfunction syndrome. Intensive Care Med.

[16] Muszynski JA (2018). Early immune function and duration of organ dysfunction in critically ill children with sepsis. Am J Respir Crit Care Med.

[17] Hall MW (2013). Innate immune function and mortality in critically ill children with influenza: a multicenter study. Crit Care Med.

[18] Hotchkiss RS (2005). Accelerated lymphocyte death in sepsis occurs by both the death receptor and mitochondrial pathways. J Immunol.

[19] Lindell RB (2022). Impaired lymphocyte responses in pediatric sepsis vary by pathogen type and are associated with features of immunometabolic dysregulation. Shock.

[20] Weiss SL (2019). Persistent mitochondrial dysfunction linked to prolonged organ dysfunction in pediatric sepsis. Crit Care Med.

[21] Marshall JC (2014). Why have clinical trials in sepsis failed?. Trends Mol Med.

[22] Opal SM (1997). Confirmatory interleukin-1 receptor antagonist trial in severe sepsis: a phase III, randomized, double-blind, placebo-controlled, multicenter trial. The Interleukin-1 Receptor Antagonist Sepsis Investigator Group. Crit Care Med.

[23] Abraham E (1998). Double-blind randomised controlled trial of monoclonal antibody to human tumour necrosis factor in treatment of septic shock. NORASEPT II Study Group. Lancet.

[24] Ranieri VM (2012). Drotrecogin alfa (activated) in adults with septic shock. N Engl J Med.

[25] Lindell RB, Meyer NJ (2023). Interrogating the sepsis host immune response using cytomics. Crit Care.

[26] Lindell RB, Meyer NJ (2024). Charting a course for precision therapy trials in sepsis. Lancet Respir Med.

[27] Wong HR (2015). Developing a clinically feasible personalized medicine approach to pediatric septic shock. Am J Respir Crit Care Med.

[28] Carcillo JA (2019). A multicenter network assessment of three inflammation phenotypes in pediatric sepsis-induced multiple organ failure. Pediatr Crit Care Med.

[29] Wong HR (2019). Prospective clinical testing and experimental validation of the pediatric sepsis biomarker risk model. Sci Transl Med.

[30] Scicluna BP (2017). Classification of patients with sepsis according to blood genomic endotype: a prospective cohort study. Lancet Respir Med.

[31] Davenport EE (2016). Genomic landscape of the individual host response and outcomes in sepsis: a prospective cohort study. Lancet Respir Med.

[32] Sweeney TE (2018). Unsupervised analysis of transcriptomics in bacterial sepsis across multiple datasets reveals three robust clusters. Crit Care Med.

[33] Cano-Gamez E (2022). An immune dysfunction score for stratification of patients with acute infection based on whole-blood gene expression. Sci Transl Med.

[34] Mi Y (2024). High-throughput mass spectrometry maps the sepsis plasma proteome and differences in patient response. Sci Transl Med.

[35] Sinha P (2023). Identifying molecular phenotypes in sepsis: an analysis of two prospective observational cohorts and secondary analysis of two randomised controlled trials. Lancet Respir Med.

[36] Wik L (2021). Proximity extension assay in combination with next-generation sequencing for high-throughput proteome-wide analysis. Mol Cell Proteomics.

[37] John CR (2020). M3C: Monte Carlo reference-based consensus clustering. Sci Rep.

[38] Fine JP, Gray RJ (1999). A proportional hazards model for the subdistribution of a competing risk. J Am Stat Assoc.

[39] Feinstein JA (2024). Pediatric complex chronic condition system version 3. JAMA Netw Open.

[40] Zou H, Hastie T (2005). Regularization and variable selection via the elastic Net. J R Stat Soc Series B Stat Methodol.

[41] Wurm MJ (2021). Regularized ordinal regression and the ordinalNet R package. J Stat Softw.

[42] Chen H (2016). Cytofkit: a bioconductor package for an integrated mass cytometry data analysis pipeline. PLoS Comput Biol.

[43] Monaco G (2016). flowAI: automatic and interactive anomaly discerning tools for flow cytometry data. Bioinformatics.

[44] Van Gassen S (2015). FlowSOM: Using self-organizing maps for visualization and interpretation of cytometry data. Cytometry A.

[45] Chan DM (2019). GPU accelerated t-distributed stochastic neighbor embedding. J Parallel Distrib Comput.

[46] Kramer A (2014). Causal analysis approaches in Ingenuity Pathway Analysis. Bioinformatics.

[47] Hanzelmann S (2013). GSVA: gene set variation analysis for microarray and RNA-seq data. BMC Bioinformatics.

[48] Liberzon A (2015). The Molecular Signatures Database (MSigDB) hallmark gene set collection. Cell Syst.

[49] Fajgenbaum DC, June CH (2020). Cytokine Storm. N Engl J Med.

[50] Clere-Jehl R (2020). JAK-STAT targeting offers novel therapeutic opportunities in sepsis. Trends Mol Med.

[51] Kwok AJ (2023). Neutrophils and emergency granulopoiesis drive immune suppression and an extreme response endotype during sepsis. Nat Immunol.

[52] Xu S (2020). Phospho-Tyr705 of STAT3 is a therapeutic target for sepsis through regulating inflammation and coagulation. Cell Commun Signal.

[53] Imbaby S (2020). Beneficial effect of STAT3 decoy oligodeoxynucleotide transfection on organ injury and mortality in mice with cecal ligation and puncture-induced sepsis. Sci Rep.

[54] Subramanian A (2005). Gene set enrichment analysis: a knowledge-based approach for interpreting genome-wide expression profiles. Proc Natl Acad Sci U S A.

[55] Young MD, Behjati S (2020). SoupX removes ambient RNA contamination from droplet-based single-cell RNA sequencing data. Gigascience.

[56] McGinnis CS (2019). DoubletFinder: doublet detection in single-cell RNA sequencing data using artificial nearest neighbors. Cell Syst.

[57] Hao Y (2024). Dictionary learning for integrative, multimodal and scalable single-cell analysis. Nat Biotechnol.

[58] Ianevski A (2022). Fully-automated and ultra-fast cell-type identification using specific marker combinations from single-cell transcriptomic data. Nat Commun.

[59] Hao Y (2021). Integrated analysis of multimodal single-cell data. Cell.

[60] Giles JR (2022). Human epigenetic and transcriptional T cell differentiation atlas for identifying functional T cell-specific enhancers. Immunity.

[61] McInnes L (2018). UMAP: uniform manifold approximation and projection. J Open Source Softw.

[62] Moore AR (2025). A consensus immune dysregulation framework for sepsis and critical illnesses. Nat Med.

[63] Andreatta M, Carmona SJ (2021). UCell: Robust and scalable single-cell gene signature scoring. Comput Struct Biotechnol J.

[64] Squair JW (2021). Confronting false discoveries in single-cell differential expression. Nat Commun.

[65] Cui A (2024). Dictionary of immune responses to cytokines at single-cell resolution. Nature.

[66] Araki K (2009). mTOR regulates memory CD8 T-cell differentiation. Nature.

[67] Jin S (2021). Inference and analysis of cell-cell communication using CellChat. Nat Commun.

[68] Borcherding N (2020). scRepertoire: An R-based toolkit for single-cell immune receptor analysis. F1000Res.

[69] Borcherding N (2024). CD4^+^ T cells exhibit distinct transcriptional phenotypes in the lymph nodes and blood following mRNA vaccination in humans. Nat Immunol.

[70] Shugay M (2018). VDJdb: a curated database of T-cell receptor sequences with known antigen specificity. Nucleic Acids Res.

[71] Tickotsky N (2017). McPAS-TCR: a manually curated catalogue of pathology-associated T cell receptor sequences. Bioinformatics.

[72] Vita R (2019). The immune epitope database (IEDB): 2018 update. Nucleic Acids Res.

[73] Zhang W (2020). PIRD: pan immune repertoire database. Bioinformatics.

[74] Davila S (2018). Viral DNAemia and immune suppression in pediatric sepsis. Pediatr Crit Care Med.

[75] Hu X (2021). The JAK/STAT signaling pathway: from bench to clinic. Signal Transduct Target Ther.

[76] https://www.ncbi.nlm.nih.gov/books/NBK570371/pdf/Bookshelf_NBK570371.pdf.

[77] Recovery Collaborative Group (2022). Baricitinib in patients admitted to hospital with COVID-19 (RECOVERY): a randomised, controlled, open-label, platform trial and updated meta-analysis. Lancet.

[78] Kalil AC (2021). Baricitinib plus Remdesivir for Hospitalized Adults with Covid-19. N Engl J Med.

[79] Guimaraes PO (2021). Tofacitinib in patients hospitalized with Covid-19 pneumonia. N Engl J Med.

[80] Casanova JL (2015). Severe infectious diseases of childhood as monogenic inborn errors of immunity. Proc Natl Acad Sci U S A.

[81] Chan AY, Torgerson TR (2020). Primary immune regulatory disorders: a growing universe of immune dysregulation. Curr Opin Allergy Clin Immunol.

[82] Lee PY (2020). Immune dysregulation and multisystem inflammatory syndrome in children (MIS-C) in individuals with haploinsufficiency of SOCS1. J Allergy Clin Immunol.

[83] Abolhassani H (2022). Inherited IFNAR1 deficiency in a child with both critical COVID-19 pneumonia and multisystem inflammatory syndrome. J Clin Immunol.

[84] Ciancanelli MJ (2015). Infectious disease. Life-threatening influenza and impaired interferon amplification in human IRF7 deficiency. Science.

[85] Kernan KF (2022). Prevalence of pathogenic and potentially pathogenic inborn error of immunity associated variants in children with severe sepsis. J Clin Immunol.

[86] Kwok AJ (2021). Host genetics and infectious disease: new tools, insights and translational opportunities. Nat Rev Genet.

[87] Chaimowitz NS (2024). JAK/STAT defects and immune dysregulation, and guiding therapeutic choices. Immunol Rev.

[88] Pietzner M (2021). Synergistic insights into human health from aptamer- and antibody-based proteomic profiling. Nat Commun.

[89] Harris PA (2009). Research electronic data capture (REDCap)--a metadata-driven methodology and workflow process for providing translational research informatics support. J Biomed Inform.

