# Supplementary material for: Aberrant STAT signaling and T cell dysregulation define a targetable pediatric sepsis endotype
Source: J Clin Invest. 2026 Jun 9;136(14):e202867. doi: 10.1172/JCI202867 (PMC13367974; doi:10.1172/JCI202867)
Supplement: Supplemental data [file jci-136-202867-s157.pdf]

**Supplementary Materials**

# Aberrant STAT signaling and T cell dysregulation define a targetable pediatric sepsis endotype

**Authors:** Robert B. Lindell[1,2]*, Samir U. Sayed[3], Jose S. Campos Duran[3], Sydney A. Sheetz[3,4], Apoorva Babu[5], Montana S. Knight[5], Andrea A. Mauracher[3], Ceire A. Hay[3], Peyton E. Conrey[3], Julie C. Fitzgerald[1], Nadir Yehya[1], Stephen T. Famularo III[4], Teresa Arroyo[4], Richard Tustin III[3], Hossein Fazelinia[5], Edward M. Behrens[6], David T. Teachey[7], Lisa R. Forbes Satter[8], Alexandra F. Freeman[9], Jenna R. E. Bergerson[9], Steven M. Holland[9], Jennifer W. Leiding[10], Scott L. Weiss[11], Mark W. Hall[12], Deanne M. Taylor[13], Rui Feng[14], E. John Wherry[2,15], Nuala J. Meyer[2,16], Sarah E. Henrickson[2,17,18]

**This PDF file includes:**


- Supplemental Clinical Methods
- Supplemental Laboratory Methods
- Supplemental Computational Methods
- Figure S1. Supporting evidence for *k*=3 clusters in Figure 2A.
- Figure S2. Sensitivity analysis of patient clustering algorithms.
- Figure S3. PCA resolving three subgroups based on expression of 24 elastic net derived severity-associated proteins.
- Figure S4. Representative gating strategy for spectral flow immune phenotyping panel.
- Figure S5. Results from single cell transcriptomics quality control and integration steps.

- Table S1. 1472 proteins measured in proteomics panel used in MODS and HC cohorts.
- Table S2. Antibodies used in 35-marker spectral flow immune phenotyping panel.
- Table S3. PCA loadings in Figure 1D, using the full proteomics dataset.
- Table S4. Severity-associated proteins identified through linear mixed-effects model after adjustment for age, sex, and day from MODS onset.
- Table S5. Etiology of MODS and computed subgroup for each patient with MODS.
- Table S6. Immunocompromised diagnoses by subgroup.
- Table S7. 368 proteins measured in proteomics panel used in IEI cohort.
- Table S8. Leading edge analysis of protein expression in Group C patients compared to HC participants.
- Table S9. Sensitivity analysis of corticosteroid effects on IL-6/JAK/STAT3 signaling by cell type.
- Table S10. Antibodies used in 13-marker T cell phosphoflow cytometry panel.
- Table S11. Modified Proulx criteria used for screening and enrollment in the MODS cohort.
- Table S12. Age and sex for each healthy control participant.
- Table S13. Final set of 1448 proteins which met quality control thresholds in all three experiments and were included in downstream analyses.
- Table S14. Gene sets used in GSEA and GSVA analysis.
- Table S15. Gene sets used in Immune Dictionary cytokine signature analysis.
- Supplemental References

## SUPPLEMENTAL CLINICAL METHODS

**Study Setting and Eligibility:** Patients were enrolled in the Pediatric Intensive Care Unit (75 beds) and Cardiac Intensive Care Unit (38 beds) at Children's Hospital of Philadelphia. Both units are combined medical and surgical ICUs.

**Inclusion Criteria:** Patients >40 weeks post-conceptual age and <18 years old were eligible if they exhibited new dysfunction of ≥2 organ systems defined by modified Proulx criteria (Table S11) within the preceding three calendar days. MODS defined by Proulx criteria is strongly associated with both short- and long-term PICU outcomes (1).

**Exclusion Criteria:** Patients were excluded if they had limitations of care orders (DNR/DNI) at the time of eligibility, clinical suspicion for brain death, or prior enrollment in the study.

### Clinical Definitions

- **MODS Onset:** Defined as the calendar day a patient first met criteria for concurrent dysfunction of ≥2 organ systems.

- **Sepsis Classification:** Sepsis was identified as the MODS inciting diagnosis based on clinical suspicion for infection by the primary treating team combined with positive microbiologic or virologic testing. Patients with "culture-negative sepsis" were classified as sepsis if they developed organ dysfunction in the setting of clinical suspicion for infection and received sepsis-directed therapies (antibiotics/antivirals), in concordance with international pediatric sepsis guidelines (2, 3).

- **Immunocompromised Status:** Defined as a diagnosis of active malignancy, prior hematopoietic cell transplant, or primary immune deficiency syndrome.

- **Outcome Measures:** Daily organ dysfunction was scored using the PELOD-2 score from MODS onset through day +28. The primary outcome was the cumulative PELOD-2 score. This metric incorporates both the severity and duration of organ failure and is associated with meaningful differences in long-term mortality (4) and health-related quality of life (5) in pediatric patients with sepsis.

## SUPPLEMENTAL LABORATORY METHODS

**Biospecimen Processing:** Blood samples were obtained in sodium heparin tubes and processed on-site within one hour of acquisition. Platelet-poor heparin plasma was separated by centrifugation, snap-frozen on dry ice, and stored at -80°C. Peripheral Blood Mononuclear Cells were isolated by density gradient centrifugation using SepMate tubes (STEMCELL Technologies, Vancouver, Canada). Whole blood was mixed 1:1 with PBS, layered onto Lymphoprep gradient. SepMate tubes were centrifuged, and the buffy coat suspension was collected and washed. Red blood cells were lysed using ACK lysis buffer. Cells were resuspended in complete RPMI (RPMI 1640 supplemented with 10% FBS, 1% L-Glutamine, 1% Pen-Strep) for counting, then resuspended in 500µl of freezing media (90% FBS, 10% DMSO) and frozen in Mr. Frosty containers at -80°C before transfer to liquid nitrogen.

**Spectral Flow Cytometry:** Full staining protocols for each panel are provided below. Details of the flow cytometry antibodies and buffers required for these protocols are available in **Table S2** (immune phenotyping panel) and **Table S10** (phosphoflow cytometry panel). All samples were acquired on a Cytek Aurora spectral flow cytometer at Children's Hospital of Philadelphia.

**Fluorescence-Activated Cell Sorting:** For scRNA-seq, cryopreserved PBMCs were thawed and stained with Live/Dead Blue (Thermo Fisher, Catalog #L34961) and anti-CD45 (BD Biosciences, Clone HI30, Catalog #751472). Using a Cytek Aurora CS cell sorter, live singlet $CD45^{+}$ cells were sorted into 1.5mL Eppendorf tubes containing 500µl of PBS + 20% FBS to ensure high viability. Sorted cells were washed twice with PBS + 10% FBS to remove sheath fluid EDTA prior to 10X library preparation.

**Single-Cell Library Preparation:** Single-cell gene expression and TCR libraries were prepared using the Chromium Next GEM Single Cell 5' Reagent Kits v2 and Single Cell Human TCR Amplification Kit per manufacturer instructions. Libraries were sequenced on an Illumina NovaSeq 6000 S2 flow cell. Demultiplexing and alignment to the GRCh38 reference genome were carried out using the Cell Ranger v8.0 pipeline (10X Genomics), generating both transcriptomic and V(D)J clonotype data for downstream analysis.

## PROTOCOL FOR THAWING AND STAINING HUMAN PBMCS FOR IMMUNE PROFILING ANALYSIS

<u>PBMC Thawing Protocol</u>

1. Prepare 15mL conical tubes with 10mL cRPMI (warmed 37°C water bath).
2. Obtain cryovials from liquid nitrogen (LN). Keep on dry ice until immediately before thaw.
3. Swirl 2 vials in 37°C water bath for ~30 seconds. Partially thawed pellet should remain.
4. Add 1mL warm cRPMI to cryovial and mix. Transfer all contents into 15mL tube.
5. Wash cryovial with 2mL warm cRPMI and transfer contents to 15mL tube.
6. Spin @ 800xg for 4 mins at room temperature (RT).
7. Decant supernatant into waste and tap vial ridge to remove excess media.
8. Resuspend cells in 1mL warm cRPMI.
9. Count cells: 10μL Trypan blue + 10μL Cell suspension.
10. Spin @ 800xg for 4 mins at RT.
11. Decant supernatant into waste and tap vial ridge to remove excess media.
12. Resuspend cells in warm cRPMI at a concentration of $5x10^6$ cells/mL.
13. Plate 200μL/well, or $1x10^6$ cells/well, in a 96-well round bottom plate.

<u>PBMC Staining Protocol</u>

1. Spin at 800xg for 4 mins, RT. Flick plate to discard supernatant.
2. Wash cells with 150μL of PBS.
3. Spin at 800xg for 4 mins, RT. Flick plate to discard supernatant.
4. Resuspend cells in 50μL **LD/Fc stain mixture**.
5. Incubate at 4°C for <u>15 minutes</u>.
6. Wash cells with 150μL of PBS.
7. Spin at 800xg for 4 mins, RT. Flick plate to discard supernatant.
8. Resuspend cells in 50μL **SS antibody mixture** in FACS Buffer + Brilliant Stain Buffer.
9. Incubate at 4°C for <u>30 minutes</u> in the dark.
10. Wash with 150μL FACS buffer.
11. Spin @ 800xg for 4 mins, RT. Flick plate to discard supernatant.
12. Resuspend cells in 50μL of **Fix/Perm reagent**.
13. Incubate at RT for <u>20 minutes</u> in the dark.
14. Wash with 150μL Perm buffer.
15. Spin @ 800xg for 4 mins, RT. Flick plate to discard supernatant.
16. Resuspend cells in 50μL **ICS antibody mixture** in Perm Buffer + Brilliant Stain Buffer.
17. Incubate at 4°C for <u>60 minutes</u> in the dark.
18. Wash with 150μL of Perm Buffer.
19. Spin @ 800xg for 4 mins, RT. Flick plate to discard supernatant.
20. Resuspend pellets in 100μL **1.6% PFA fixative** and transfer to bullet tubes.
21. Cells can be held at 4°C overnight prior to acquisition.

## PROTOCOL FOR THAWING AND STAINING HUMAN PBMCS FOR AURORA CS SORTING

PBMC Thawing Protocol

1. Prepare 15mL conical tubes with 10mL cRPMI (warmed 37°C water bath).
2. Obtain PBMC cryovials from LN and keep on dry ice until immediately before thaw.
3. Swirl 2 vials in 37°C water bath for ~30 seconds. Partially thawed pellet should remain.
4. Add 1mL warm cRPMI to cryovial and mix. Transfer all contents into 15mL tube.
5. Wash cryovial with 2mL warm cRPMI and transfer contents to 15mL tube.
6. Spin @ 800xg for 4 mins at RT.
7. Decant supernatant into waste and tap vial ridge to remove excess media.
8. Resuspend cells in 1mL warm cRPMI.
9. Count cells with 10µL trypan blue + 10µL cell suspension.
10. Lift $1x10^6$ live cells into a new labeled tube for staining.

PBMC Staining Protocol

1. Spin at 800xg for 4 mins, RT. Decant/tap to remove excess media.
2. Wash cells with 1mL of FACS buffer.
3. Spin at 800xg for 4 mins, RT. Decant/tap to remove excess media.
4. Resuspend cells in 100µL **Stain Mixture**.
5. Incubate at RT for 30 minutes in the dark.
6. Wash cells with 1mL of FACS buffer.
7. Spin at 800xg for 4 mins, RT. Decant/tap to remove excess media.
8. Resuspend cells in 500µL FACS Buffer and pass through blue cell strainer into FACS tube.
9. Cover and keep on wet ice. Cells are ready for sorting.

Prepare Sort Tubes and Resuspension Media

1. Prepare three 1.5ml eppendorf tubes with 500µL of PBS + 20% FBS for each sample.
2. Prepare 10% FBS Resuspension Media.

Cell Washing and Resuspension

1. After sorting, spin down cells in microcentrifuge 800xg for 5 mins, 4 degrees.
2. Aspirate supernatant and wash with 500µL PBS + 10% FBS.
3. Spin down cells in microcentrifuge 800xg for 5 mins, 4 degrees.
4. Aspirate supernatant and wash with 500µL PBS + 10% FBS.
5. Spin down cells in microcentrifuge 800xg for 5 mins, 4 degrees.
6. Goal concentration is 1200 cells per microliter. Calculate resuspension volume.
7. Aspirate supernatant and resuspend in PBS + 10% FBS.
8. Cover and keep on wet ice. Cells are ready for 10x processing.

**PROTOCOL FOR THAWING AND STAINING HUMAN PBMCS FOR PHOSPHOFLOW CYTOMETRY**

PBMC Stimulation Protocol

1. Prepare a 2µg/mL solution of anti-CD3 in sterile PBS.
    - InVivoMAb Anti-Human CD3, BioXCell, UCHT1 (Leu-4) (T3), BE0231
2. Add 200µL of diluted anti-CD3 solution to stimulation wells of a flat bottom 96 well plate.
3. Add 200µL of PBS to unstimulated wells in the same flat bottom 96 well plate.
4. Cover the plate with a plate sealer and store at 4°C overnight.

PBMC Thawing Protocol

5. Prepare 15mL conical tubes with 10mL cRPMI (warmed 37°C water bath).
6. Obtain PBMC cryovials from LN and keep on dry ice until immediately before thaw.
7. Swirl 2 vials in 37°C water bath for ~30 seconds. Partially thawed pellet should remain.
8. Add 1mL warm cRPMI to cryovial and mix. Transfer all contents into 15mL tube.
9. Wash cryovial with 2mL warm cRPMI and transfer contents to 15mL tube.
10. Spin @ 800xg for 4 mins at RT.
11. Decant supernatant into waste and tap vial ridge to remove excess media.
12. Resuspend cells in 1mL warm cRPMI.
13. Count cells with 10µL trypan blue + 10µL cell suspension.
14. Spin @ 800xg for 4 mins at RT.
15. Decant supernatant into waste and tap vial ridge to remove excess media.
16. Resuspend cells in warm cRPMI at a concentration of $2.5 \times 10^6$ cells/mL.

PBMC Stimulation Protocol Continued

17. Remove anti-CD3 flat bottom plate from 4°C and carefully aspirate coating solution and PBS from wells.
18. Plate 100µL/well, or $2.5 \times 10^5$ cells/well, in the 96-well flat bottom plate coated with anti-human CD3 antibody and PBS.
19. Prepare a 2µg/mL of an anti-human CD28 in AIM-V media. *Add cytokine if desired here.*
    - BD FastImmune Anti-Human CD28/CD49d, BD, L293/L25, 347690
20. Add 100µL of stimulation media to each stimulated well.
21. Add 100µL of AIM-V media to each unstimulated well.
22. Incubate at 37°C, 5% $CO_2$ for 24 hours.

PBMC Staining Protocol

23. Spin at 800xg for 4 mins, RT. Flick plate to discard supernatant.
24. Resuspend cells in 50µL of **pFlow surface stain mixture** and incubate at RT for 30 minutes.
25. Wash cells with 150µL of FACS Buffer.
26. Spin at 800xg for 4 minutes at RT. Flick plate to discard supernatant.
27. Resuspend cells in 100µL of **1.6% PFA** and incubate at RT for 15 minutes.
28. Spin at 800xg for 4 minutes at RT. Flick plate to discard supernatant.
29. Add 150µL/well of **cold MeOH**.
30. Wrap plate in foil and incubate at -20°C overnight.
31. Spin at 800xg for 5 minutes at RT. Flick plate to discard supernatant.
32. Wash cells with 200µL of Perm Buffer.

33. Spin at 800xg for 4 minutes at RT. Flick plate to discard supernatant.
34. Wash cells with 200µL of Perm Buffer.
35. Spin at 800xg for 4 minutes at RT. Flick plate to discard supernatant.
36. Resuspend cells in 50µL of **pFlow ICS stain mixture** and incubate at RT for <u>60 minutes</u>.
37. Wash cells with 150µL of Perm Buffer.
38. Spin at 800xg for 4 minutes at RT. Flick plate to discard supernatant.
39. Resuspend cells in 125µL of **1.6% PFA**.
40. Cover and keep on wet ice. Cells are ready for acquisition.

## SUPPLEMENTAL COMPUTATIONAL METHODS

**Statistical Framework:** Categorical variables were analyzed by $\chi^2$ test. Comparisons of continuous variables between two groups were performed using the Wilcoxon rank-sum test with Benjamini-Hochberg adjustment to control the false discovery rate. Comparisons of continuous variables across three or more groups were performed using the Kruskal-Wallis test. Pairwise comparisons were subsequently performed using Dunn's post-hoc test with Benjamini-Hochberg adjustment to control the false discovery rate.

**Identification of subgroups:** We constructed linear mixed effects models for each protein to determine the association between normalized protein expression and cumulative PELOD-2 score, with age and sex modeled as fixed effects and day from MODS onset modeled as a random effect. Proteins which were significantly associated with cumulative PELOD-2 score after Benjamini-Hochberg correction (FDR $p<0.05$) were retained for further analysis. This supervised feature selection strategy was explicitly selected to discover endotypes relevant to clinical outcomes. We used consensus clustering to define subgroups and identified optimal *k* via the Monte Carlo reference-based consensus clustering algorithm (6) which uses the proportion of ambiguous clustering (PAC) score to test the hypothesis that $k=n$ clusters is more informative than $k=1$. Optimal number of clusters in the MODS data (k=3) was confirmed by Monte Carlo bootstrapping, elbow method, and gap statistic. To ensure that this approach did not yield circular artifacts, the biological validity of the resulting clusters was confirmed using orthogonal data modalities (e.g., spectral flow cytometry, scRNA-seq) not included in the clustering model. The clinical and proteomic datasets contained no missing data elements, thus imputation was not necessary for our analyses.

**Sensitivity analysis of patient clustering algorithms:** To verify that the identified patient subgroups were robust to the choice of clustering algorithm, we performed a sensitivity analysis using spectral clustering, a graph-theoretic approach that partitions data based on topological connectivity. We constructed a k-nearest neighbor graph ($k=19$) using the severity-associated protein feature set and partitioned the resulting Laplacian matrix into three clusters. Concordance between the primary Consensus Clustering and Spectral Clustering was quantified using the Adjusted Rand Index (ARI) and overall percent agreement. We observed a high degree of concordance between methods (ARI: 0.73; Concordance: 91%), confirming that the subgroups reflect stable intrinsic structures within the proteomic data rather than artifacts of a specific algorithm. Concordance was visualized by projecting cluster assignments onto the first two principal components of the proteomic dataset, as shown in **Figure S2**.

**Competing risk survival analysis:** We fit a proportional subdistribution hazards regression model to assess the effect of covariates on the subdistribution of death and survival to PICU discharge in a competing risk setting and then estimated the cumulative incidence function from this model for each subgroup and outcome using the Fine and Gray model (7). We then tested the hypothesis that the subdistribution hazard differed between Group C and Group A/B for both death and survival to PICU discharge.

**Model reduction via ordinal elastic net:** We defined a parsimonious protein signature to classify subgroups by training an ordinal elastic net model (8) for feature selection using the

severity-associated proteins previously identified through linear mixed effects modeling. This approach fit a semi-parallel elementwise link multinomial-ordinal regression model with elastic net penalty which defines coefficients for each protein:subgroup and then shrinks the model to a single coefficient for each protein via elastic net penalty to minimize Gini index. We assessed the performance of the parsimonious elastic net protein set to discriminate the three subgroups using PCA, linear mixed effects post-estimation, and polytomous discrimination index (9).

**Analysis of spectral flow cytometry data:** *Data preprocessing:* Raw .fcs files were compensated on-instrument (Cytek Aurora) using single-stain controls. Post-acquisition, data were arcsinh transformed (10) and quality controlled using flowAI (11) to automatically remove acquisition anomalies related to flow rate, signal instability, and dynamic range. *Metaclustering:* We performed FlowSOM metaclustering (12) to identify 14 immune cell populations by surface and intracellular marker expression. PBMCs were first clustered into k=60 FlowSOM metaclusters and then combined in a stepwise fashion to generate the 14 canonical populations identified in the figure and used for downstream analysis. Metacluster similarity was determined by surface and intracellular marker expression and tSNE-CUDA (13) proximity and then confirmed with manual gating. Proliferation and activation markers were analyzed using bivariate plots. *Phosphoflow analysis:* Phosphoflow cytometry data were analyzed using FlowJo v10.9 (BD Biosciences). T cells were identified via manual gating as Live singlet $CD3^+CD19^-$ $CD56^-$ events. Major lineage subsets ($CD4^+$ and $CD8^+$) were subsequently identified. Within each lineage, non-naïve T cells (Tnn) were defined by excluding the naïve population ($CD45RA^+CD27^+$) to include central memory, effector memory, and TEMRA subsets.

**Pathway enrichment analysis:** We first performed pathway enrichment analysis by subgroup by analyzing estimated protein expression in Group C patients (adjusted for age, sex, and PELOD-2 score) with Ingenuity Pathway Analysis (Qiagen) (14). We then analyzed pathway expression in the protein dataset in bulk by GSEA (15) and at the individual patient level using GSVA (16), with a focus on enrichment of five canonical proinflammatory pathways using Human Molecular Signatures Database (MSigDB) Hallmark gene sets (17):

“TNF Signaling via NFκB” (HALLMARK_TNFA_SIGNALING_VIA_NFKB),
“IL-6/JAK/STAT3 Signaling” (HALLMARK_IL6_JAK_STAT3_SIGNALING),
“IL-2/JAK/STAT5 Signaling” (HALLMARK_IL2_STAT5_SIGNALING),
“IFN-γ Response” (HALLMARK_INTERFERON_GAMMA_RESPONSE),
“PI3K/AKT/mTOR Signaling” (HALLMARK_PI3K_AKT_MTOR_SIGNALING).

We also applied GSVA to single cell transcriptomics data in a similar approach using UCell (18) and extended our analysis to include relevant immunometabolic pathways using MSigDB KEGG and Hallmark gene sets:

“KEGG Glycolysis” (KEGG_GLYCOLYSIS_GLUCONEOGENESIS)
“KEGG Oxidative Phosphorylation” (KEGG_OXIDATIVE_PHOSPHORYLATION)
“KEGG mTOR” (KEGG_MTOR_SIGNALING_PATHWAY)
“Glycolysis” (HALLMARK_GLYCOLYSIS),
“Oxidative Phosphorylation” (HALLMARK_OXIDATIVE_PHOSPHORYLATION).

A list of the gene sets used in GSEA and GSVA analysis in this manuscript are included in **Table S14**. Throughout the manuscript, we refer to Hallmark pathways as “modules” as opposed to

"gene sets" because the analytic approach is applied to both proteomics data (via GSVA) and transcriptomics data (via UCell).

**Single cell transcriptomics analytic pipeline:** Transcriptomic data were analyzed using the Seurat 5.0 preprocessing and integration pipeline (19). After review of standard quality control metrics, cells with less than 200 genes, greater than 3 median absolute deviations above the median, and >10% mitochondrial RNA content were removed from downstream analysis. After normalization and scaling using the scTransform function in Seurat, we performed anchor-based RPCA integration across samples and conditions using the top 3000 genes as anchors. Following linear dimension reduction using PCA, the data was clustered using the Louvain algorithm and projected into 2D coordinates using the RunUMAP function in Seurat. The cell identities were then inferred using the scType package (20) followed by curated manual annotation using canonical marker genes to define 14 immune cell populations by transcriptional profile. Results of each quality control and integration step are shown in **Figure S5**. Pseudobulk analysis was performed by condition, sample, and cell type using Seurat's AggregateExpression function. Differential expression analysis was performed using the FindMarkers function in Seurat. Cell-cell communication analysis was performed using CellChat (21). To assess associations between effector cell pathways and T cell immunometabolic states, we employed generalized linear mixed-effects models, treating module enrichment scores as fixed effects and patient identity as a random effect.

**T cell receptor sequencing analytic pipeline:** T-cell receptor (TCR) sequencing data were analyzed using the scRepertoire package (22). VDJ output from the Cell Ranger pipeline (10x Genomics) were integrated with single-cell RNA sequencing gene expression data using the combineExpression() function. Clonotypes were defined based on CDR3 amino acid sequences and visualized using clonotype homeostasis and proportional abundance plots. Diversity was quantified using Shannon diversity (23). To further characterize TCR sequence features, clonotype-level annotation was performed using the TCR Repertoire Explorer (TReX) package (24).

**Immune Dictionary cytokine signature analysis:** To assess cytokine-specific transcriptional responses in our scRNA-seq dataset, we applied the Immune Dictionary framework, which maps cytokine signaling to downstream gene expression profiles in specific immune cell types. Single-cell transcriptomic data were scored using curated cytokine-response gene signatures derived from the published experimental datasets (25). A list of the gene sets used in this analysis are included in **Table S15**. Module enrichment scores were computed using UCell for each cytokine response transcriptional signature, enabling cell-specific quantification of cytokine signaling activity.

**Sensitivity analysis of corticosteroid effect:** To assess the potential confounding effect of corticosteroid treatment on transcriptional signatures, we performed a sensitivity analysis comparing lineage-specific IL-6/JAK/STAT3 module enrichment scores between Group C patients who received corticosteroids (n=4) and those who did not (n=5). We calculated Cliff's Delta (δ), a non-parametric effect size measure quantifying the difference between two distributions, to estimate the magnitude of separation between treated and untreated groups. Effect magnitudes were interpreted using standard thresholds (26): negligible ($|\delta| < 0.15$), small

($0.15 \leq |\delta| < 0.33$), medium ($0.33 \leq |\delta| < 0.47$), and large ($|\delta| \geq 0.47$). A positive $\delta$ indicates higher scores in the "No Steroids" group (consistent with steroid-mediated suppression), while a negative $\delta$ indicates higher scores in the "Yes Steroids" group (consistent with a lack of response to corticosteroids).

**Figure S1. Supporting evidence for *k*=3 clusters in Figure 2A.**

(A) This cumulative distribution function (CDF) plot demonstrates increased cluster stability up to *k*=3 (as indicated by flatter line), with decreasing cluster stability with finer clustering.

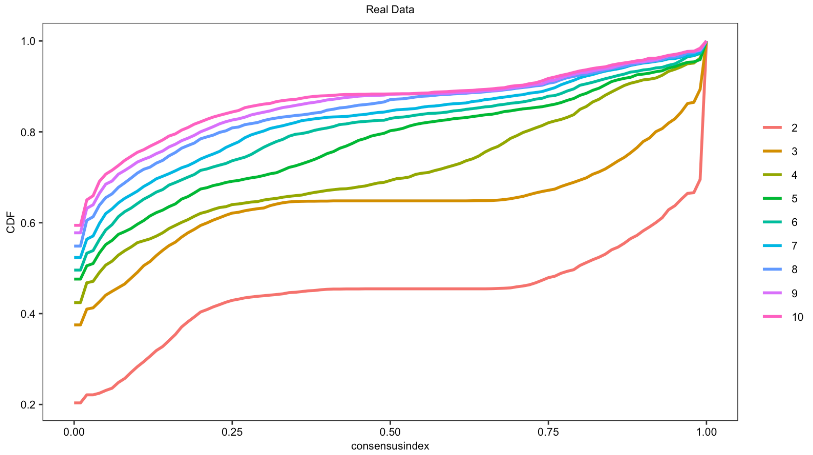


(B) Relative Cluster Stability Index (RCSI) is highest at *k*=3.

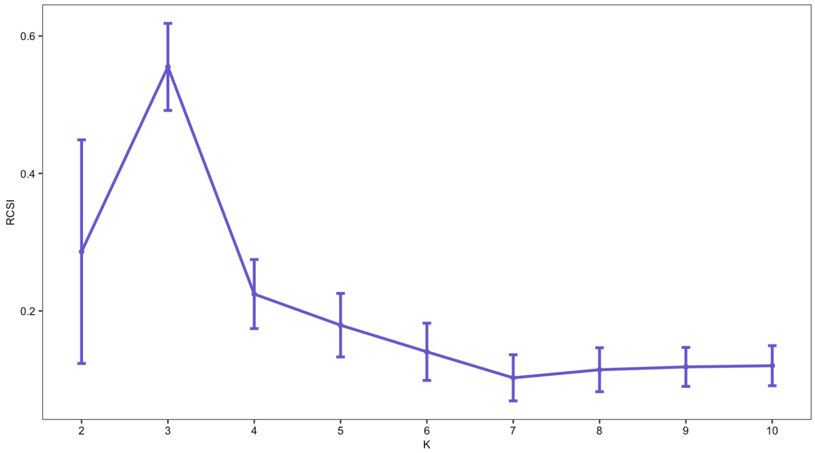


(C) M3C calculates empirical *p*-values from reference distributions to test the null hypothesis that *k*=1. In our analysis, at *k*=3 the null hypothesis is rejected (p=0.038).

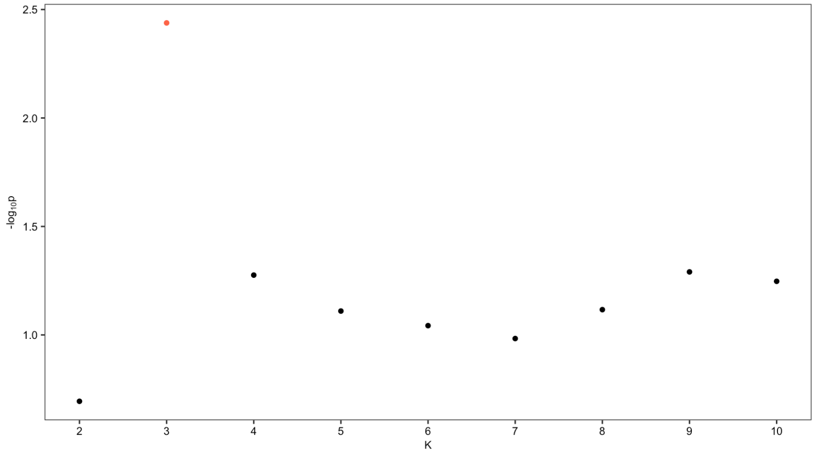

**Figure S2. Sensitivity analysis of patient clustering algorithms.**

(A) To verify that the identified patient subgroups were robust to the choice of clustering algorithm, we performed a sensitivity analysis using Spectral Clustering. On PCA, we see a high degree of concordance between consensus clustering and spectral clustering, suggesting that these groups reflect stable intrinsic structures within the proteomic data rather than artifacts of a specific algorithm.

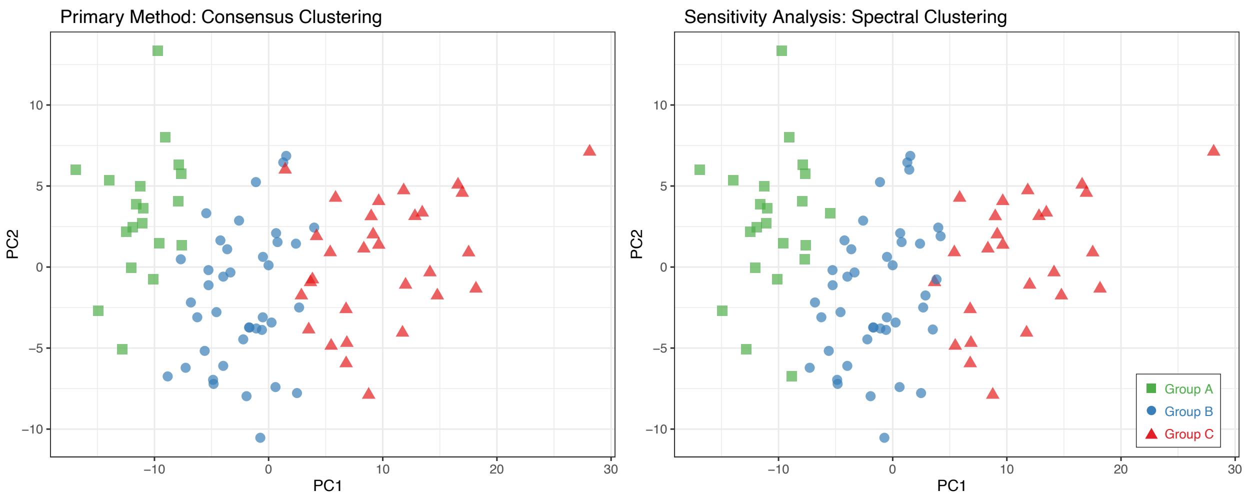


(B) We quantified concordance between our primary analysis (consensus clustering) and sensitivity analysis (spectral clustering) using the Adjusted Rand Index (ARI) and overall percent agreement. The confusion matrix below shows the high degree of concordance between methods (ARI: 0.73, concordance: 90.9%).

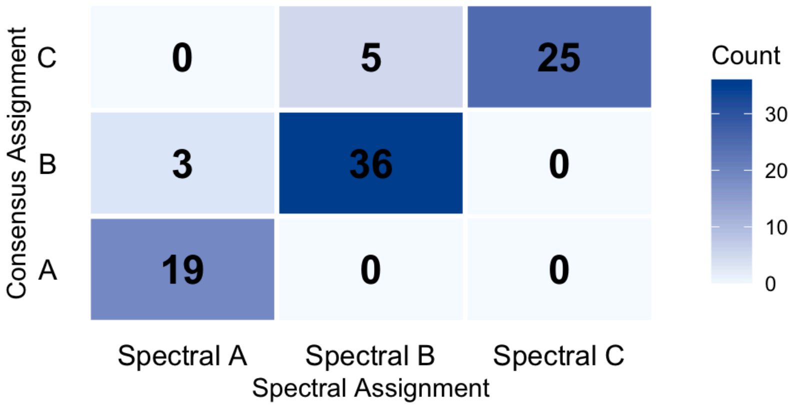

**Figure S3. PCA resolving three subgroups based on expression of 24 elastic net derived severity-associated proteins.**

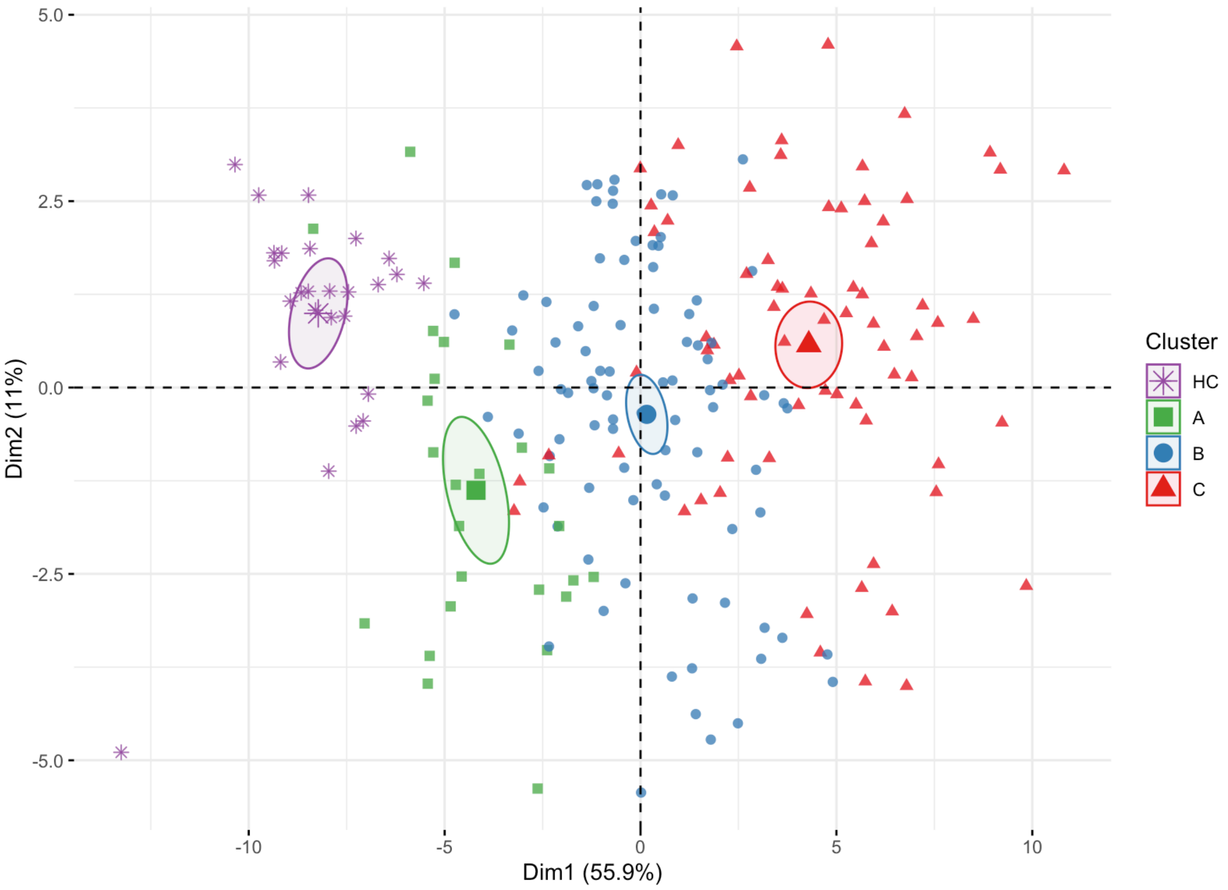


PCA dimensionality reduction demonstrates effective resolution of three MODS subgroups based on expression of 24 elastic net derived severity-associated proteins, with the majority of variance within the dataset defined by Dimension 1 as expected.

**Figure S4. Representative gating strategy and phenotype plots for spectral flow immune phenotyping panel.**

**(A) Canonical gating strategy**

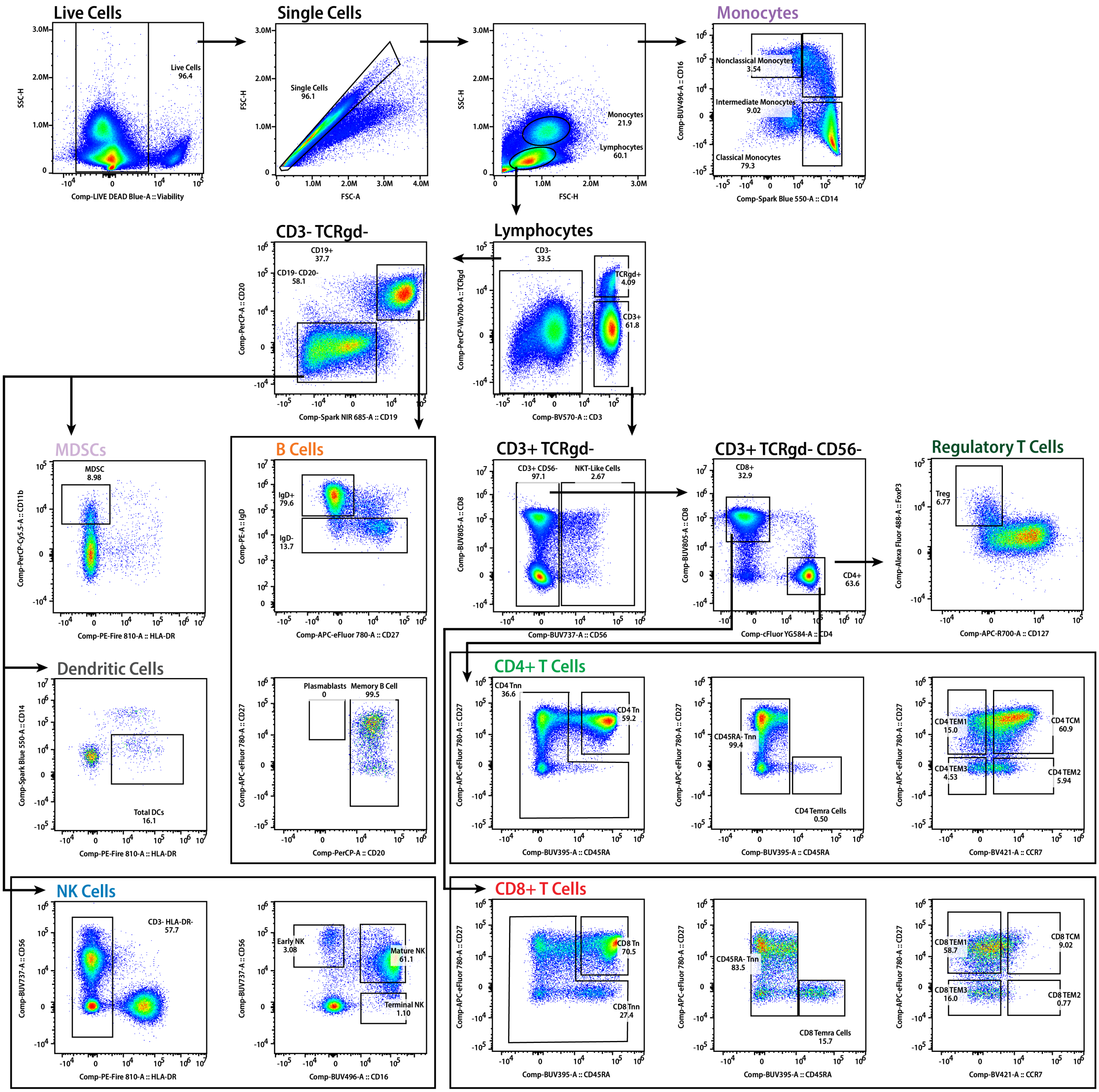

**(B) Representative spectral flow cytometry immune phenotyping plots, demonstrating concordance between manually gated subsets and tSNE projection.**

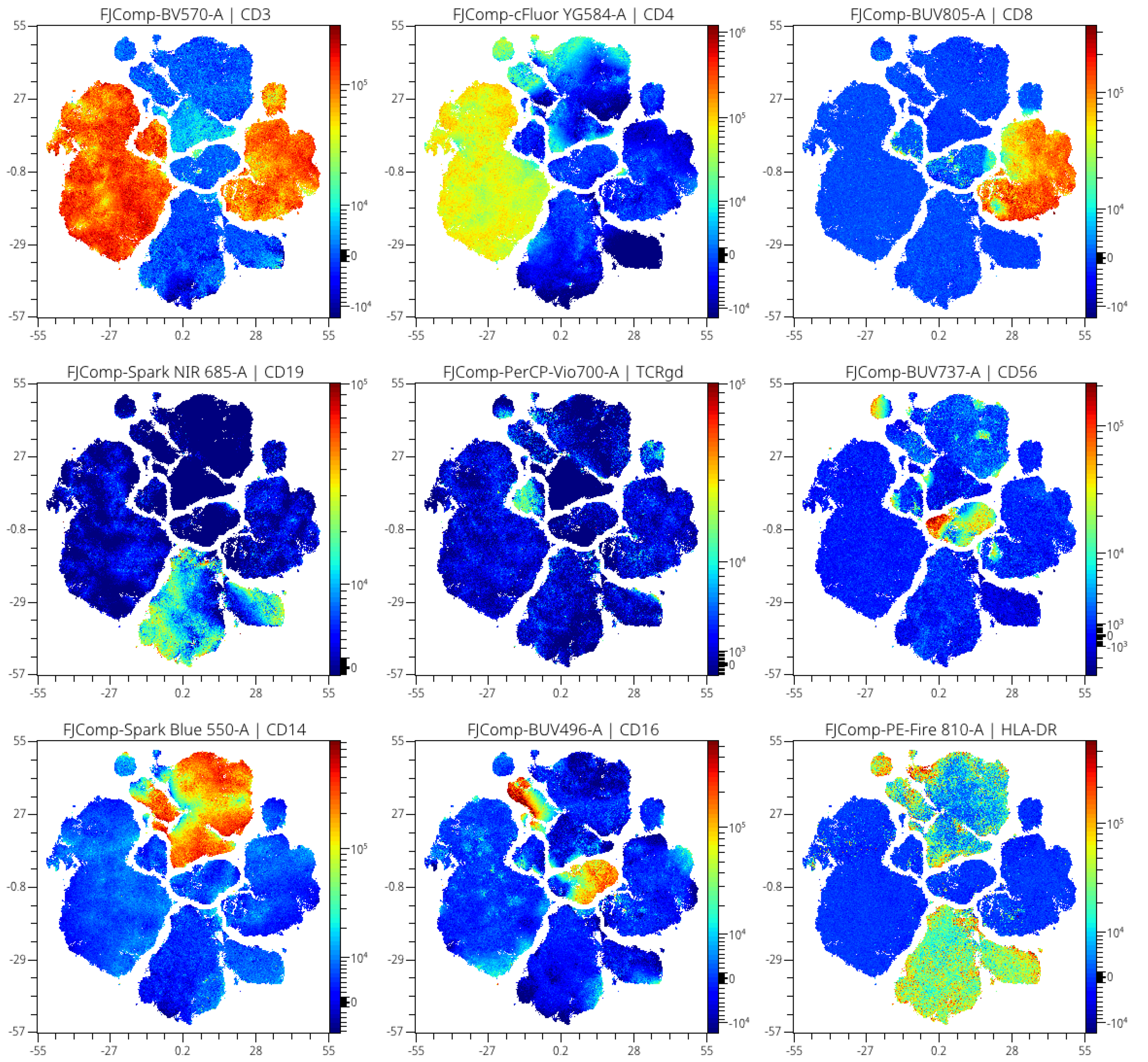

**Figure S5. Results from single cell transcriptomics quality control and integration steps.**

**(A) Quality Control**

After review of standard quality control metrics, cells with less than 200 genes, greater than 3 median absolute deviation above the median, and >10% mitochondrial RNA content were removed from downstream analysis.

After applying these quality control steps, sample quality was consistent across groups.

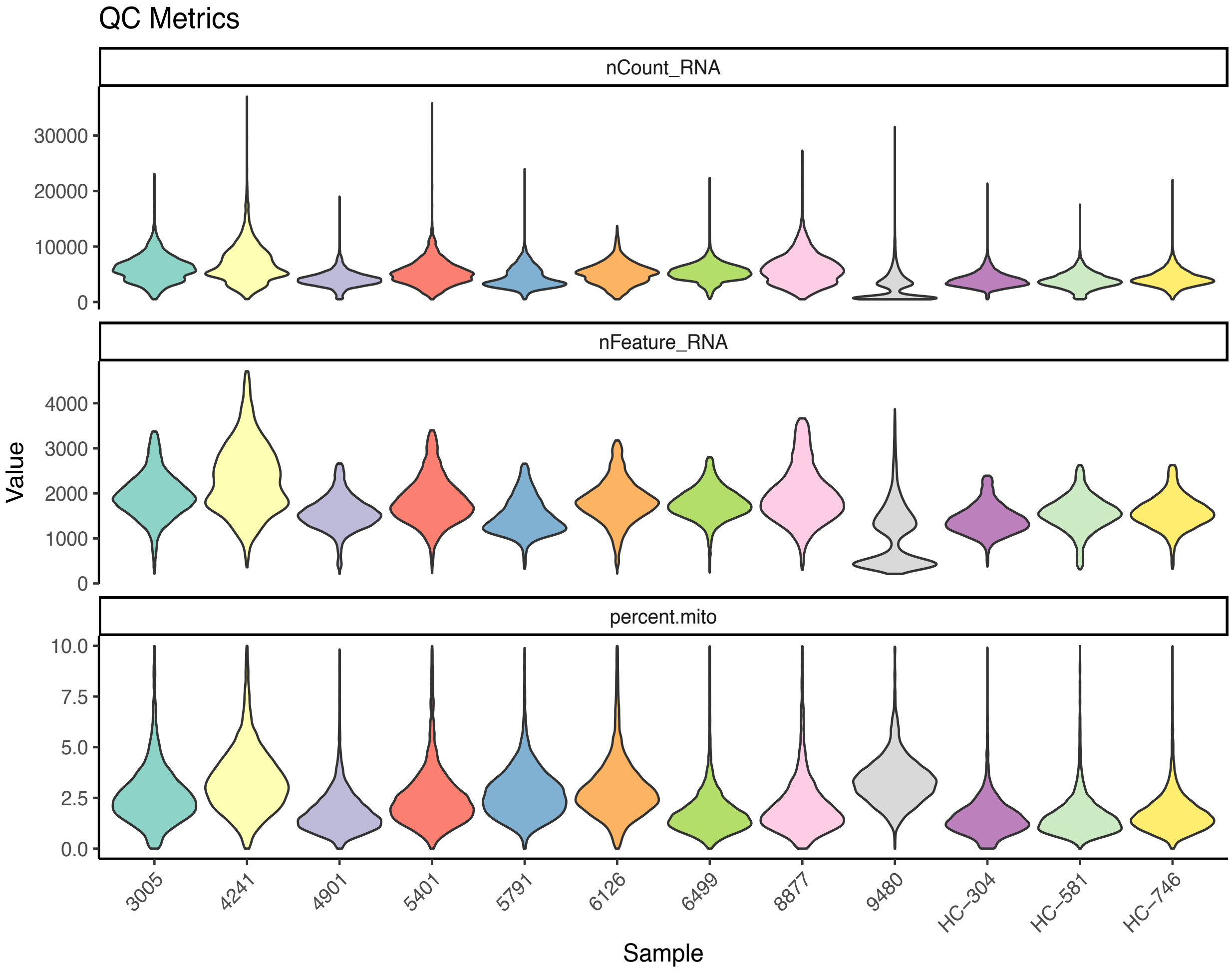

**(B) RPCA Integration**

Unintegrated data demonstrates differences between Group C Patients and HC Participants.

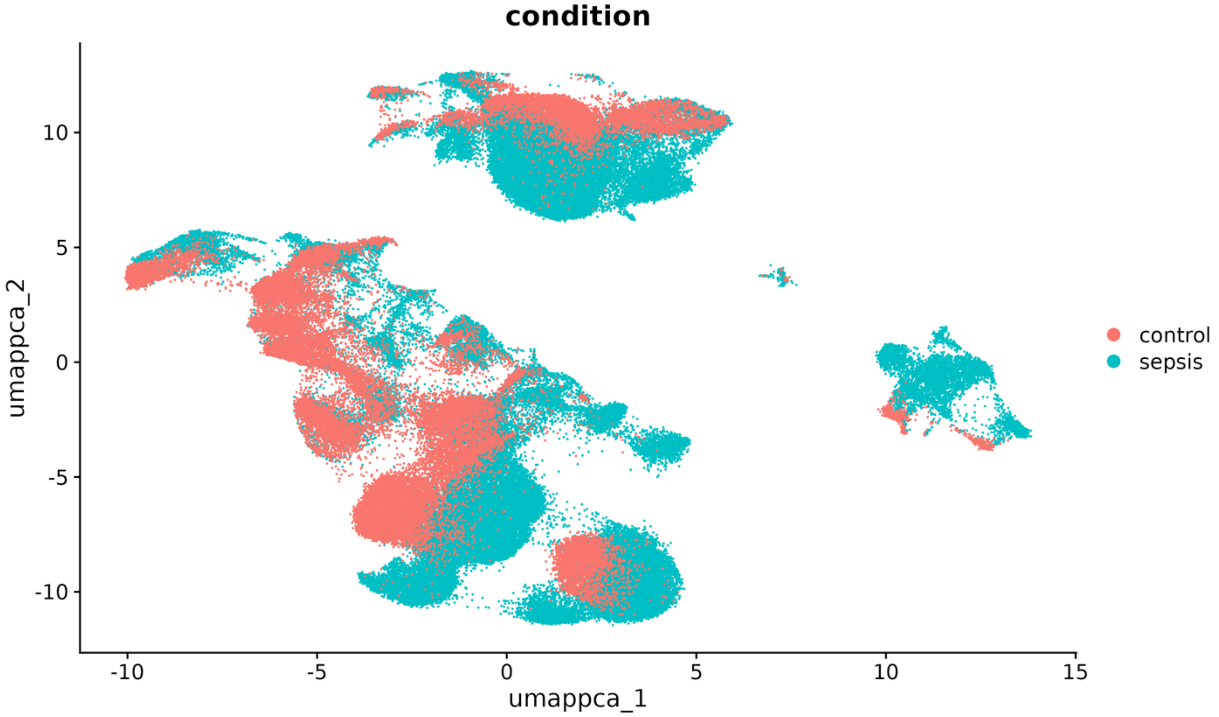


Integration with RPCA resolves discrepancies between Group C Patients and HC Participants.

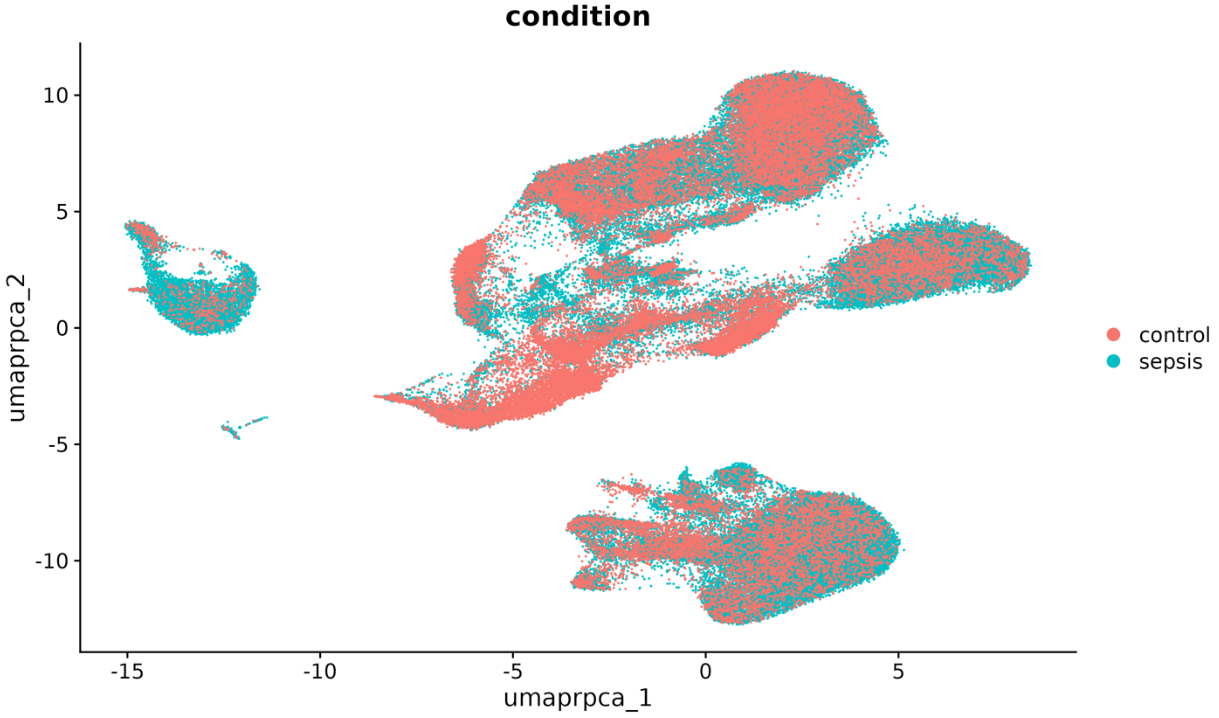

After integration, each RPCA cluster contains cells from MODS patients and HC participants.

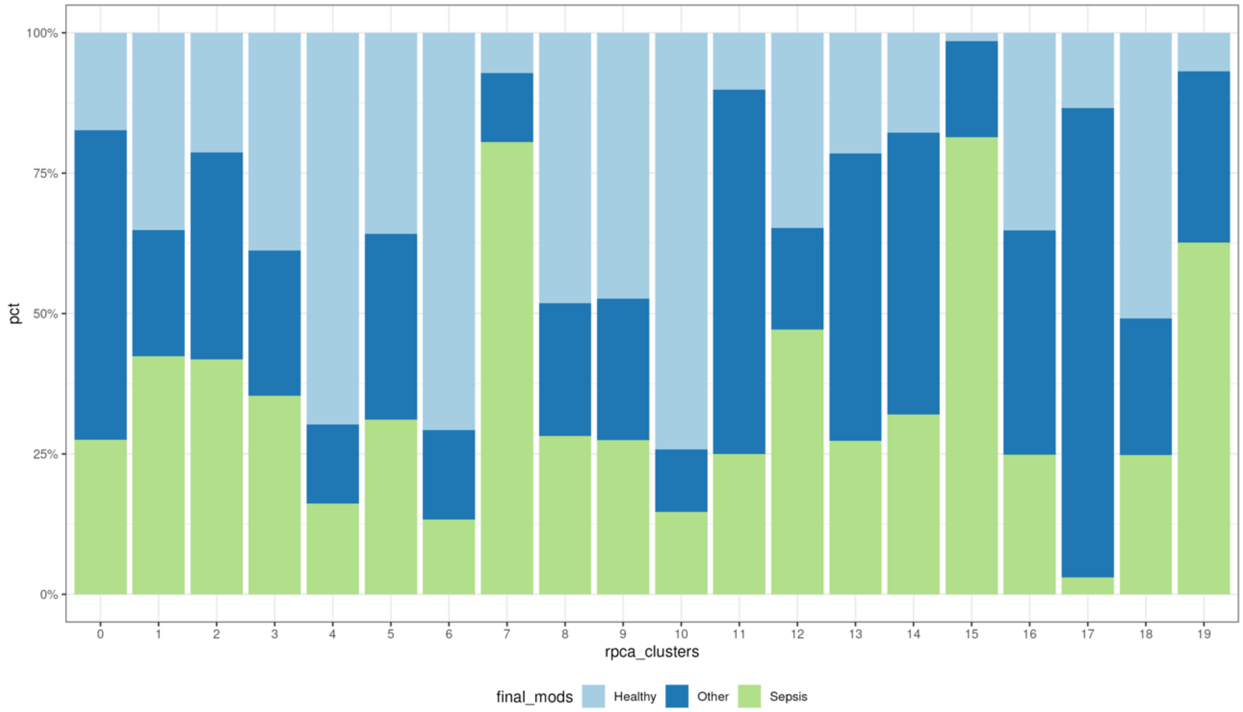

**Table S1. 1472 proteins measured in proteomics panel used in MODS and HC cohorts.**

*Due to its size, Table S1 is available in the supplemental spreadsheet.*

**Table S2. Antibodies used in 35-marker spectral flow immune phenotyping panel.**

| MARKER | FLUORORPHORE | VENDOR | CLONE | CATALOG NO. |
|---|---|---|---|---|
| CD45RA | BUV395 | BD Biosciences | H100 | 740298 |
| Live/Dead | LD Blue | Thermo Fisher | N/A | L34961 |
| CD16 | BUV496 | BD Biosciences | 3G8 | 612944 |
| CD26 | BUV563 | BD Biosciences | L272 | 749181 |
| CD39 | BUV661 | BD Biosciences | TU66 | 749967 |
| CD56 | BUV737 | BD Biosciences | NCAM16.2 | 612767 |
| CD8 | BUV805 | BD Biosciences | SK1 | 612889 |
| CCR7 | BV421 | BioLegend | G043H7 | 353208 |
| CD123 | Super Bright 436 | Thermo Fisher | 6H6 | 62-1239-42 |
| CD161 | eFluor 450 | Thermo Fisher | HP-3G10 | 48-1619-41 |
| ICOS | BV480 | BD Biosciences | DX29 | 746248 |
| Ki67 | BV510 | BD Biosciences | B56 | 563462 |
| CD3 | BV570 | BioLegend | UCHT1 | 300436 |
| CCR4 | BV605 | BioLegend | L291H4 | 359418 |
| CD95 | BV650 | BioLegend | DX2 | 305642 |
| CCR6 | BV711 | BioLegend | G034E3 | 353436 |
| CXCR5 | BV750 | BD Biosciences | RF8B2 | 747111 |
| PD-1 | BV785 | BioLegend | EH12.2H7 | 329930 |
| Foxp3 | Alexa Fluor 488 | BioLegend | 259D | 320212 |
| CD14 | Spark Blue 550 | BioLegend | 63D3 | 367148 |
| CD20 | PerCP | BioLegend | 2H7 | 302324 |
| CD11b | PerCP-Cy5.5 | BioLegend | ICRF44 | 301328 |
| TCRγδ | PerCP-eFluor 710 | Thermo Fisher | B1.1 | 46-9959-42 |
| IgD | PE | BioLegend | IA6-2 | 348204 |
| CD4 | cFluor YG584 | CYTEK | SK3 | R7-20041-100T |
| Tbet | PE-Cy5 | Thermo Fisher | 4B10 | 15-5825-80 |
| CD25 | PE-Alexa Fluor 700 | Thermo Fisher | CD25-3G10 | MHCD2524 |
| CXCR3 | PE-Cy7 | BioLegend | G025H7 | 353720 |
| HLA-DR | PE-Fire 810 | BioLegend | L243 | 307683 |
| CRTH2 | APC | BioLegend | BM16 | 350110 |
| CD11c | Alexa Fluor 647 | BioLegend | 3.9 | 301620 |
| CD19 | Spark NIR 685 | BioLegend | HIB19 | 302270 |
| CD127 | APC-R700 | BD Biosciences | HIL-7R-M21 | 565185 |
| CD27 | APC-eFluor780 | Thermo Fisher | O323 | 47-0279-42 |
| CD38 | APC/Fire 810 | BioLegend | HIT2 | 303550 |

**Table S3. PCA loadings in Figure 1D, using the full proteomics dataset.**

| DIMENSION 1<br>(TOP 25 PROTEINS) | |
|---|---|
| **PROTEIN NAME** | **CONTRIBUTION** |
| POLR2F | 0.92969158 |
| MAD1L1 | 0.91356503 |
| TXLNA | 0.89474736 |
| ELOA | 0.86811405 |
| DPY30 | 0.86400025 |
| EGLN1 | 0.85809847 |
| ZBTB17 | 0.85543741 |
| APBB1IP | 0.85335406 |
| PXN | 0.85301337 |
| GPKOW | 0.85281899 |
| DFFA | 0.85090827 |
| OGFR | 0.84328415 |
| HDGF | 0.841057 |
| PLAUR | 0.84001585 |
| NUDC | 0.83925242 |
| INPP1 | 0.83863173 |
| GFER | 0.83484888 |
| CKAP4 | 0.8330877 |
| RAD23B | 0.82862877 |
| SRP14 | 0.82675401 |
| NMNAT1 | 0.82477831 |
| NBN | 0.82470521 |
| EZR | 0.82439488 |
| DCTPP1 | 0.82416125 |
| HEXIM1 | 0.82383476 |

| DIMENSION 2<br>(TOP 25 PROTEINS) | |
|---|---|
| **PROTEIN NAME** | **CONTRIBUTION** |
| ARHGEF12 | 0.92927293 |
| DBNL | 0.91717031 |
| CASP3 | 0.91624786 |
| PPP1R12A | 0.90434238 |
| SRC | 0.90335717 |
| CRKL | 0.90060822 |
| NT5C3A | 0.89589151 |
| PLXNA4 | 0.89309739 |
| MAP2K6 | 0.88974514 |
| CA13 | 0.88624933 |
| TBC1D23 | 0.88578306 |
| CLIP2 | 0.88413467 |
| TJAP1 | 0.88285037 |
| SKAP2 | 0.87773049 |
| AXIN1 | 0.87715071 |
| PDLIM7 | 0.87685547 |
| MANF | 0.87614294 |
| CIAPIN1 | 0.87457034 |
| MYO9B | 0.87420508 |
| SNAP29 | 0.87219403 |
| MAP3K5 | 0.87165308 |
| DNAJA2 | 0.86881494 |
| CORO1A | 0.86234331 |
| CRACR2A | 0.86216539 |
| SNAP23 | 0.85969907 |

**Table S4. Severity-associated proteins identified through linear mixed-effects model after adjustment for age, sex, and day from MODS onset.**

*Due to its size, Table S4 is available in the supplemental spreadsheet.*

## Table S5. Etiology of MODS and computed subgroup for each MODS patient.

| HASHED PATIENT ID | MODS ETIOLOGY | DETAILED ETIOLOGY | SUBGROUP |
|---|---|---|---|
| 1018 | Cardiopulmonary Bypass | Cardiopulmonary Bypass | B |
| 1168 | Sepsis | Culture Negative Sepsis | B |
| 1234 | Sepsis | Culture Negative Sepsis | C |
| 1344 | Cardiopulmonary Bypass | Cardiopulmonary Bypass | B |
| 1426 | Sepsis | Culture Negative Sepsis | B |
| 1501 | Non-infected | Exacerbation of Chronic Disease | B |
| 1521 | Sepsis | Bacterial Sepsis | B |
| 1523 | Non-infected | Cardiac Arrest | B |
| 1568 | Non-infected | No Cause Identified | B |
| 1620 | Non-infected | No Cause Identified | B |
| 1670 | Sepsis | Culture Negative Sepsis | B |
| 1677 | Non-infected | Exacerbation of Chronic Disease | C |
| 1845 | Sepsis | Viral Sepsis | A |
| 1947 | Non-infected | No Cause Identified | B |
| 1973 | Non-infected | Cardiac Arrest | A |
| 2040 | Non-infected | Postoperative Status | C |
| 2056 | Cardiopulmonary Bypass | Cardiopulmonary Bypass | B |
| 2166 | Non-infected | No Cause Identified | A |
| 2243 | Cardiopulmonary Bypass | Cardiopulmonary Bypass | B |
| 2274 | Non-infected | No Cause Identified | C |
| 2309 | Cardiopulmonary Bypass | Cardiopulmonary Bypass | B |
| 2314 | Non-infected | No Cause Identified | C |
| 2393 | Sepsis | Culture Negative Sepsis | B |
| 2439 | Cardiopulmonary Bypass | Cardiopulmonary Bypass | B |
| 2726 | Non-infected | No Cause Identified | C |
| 2997 | Non-infected | No Cause Identified | A |
| 3005 | Sepsis | Bacterial Sepsis | C |
| 3134 | Sepsis | Bacterial Sepsis | C |
| 3227 | Sepsis | Viral Sepsis | C |
| 3365 | Sepsis | Culture Negative Sepsis | C |
| 3502 | Cardiopulmonary Bypass | Cardiopulmonary Bypass | B |
| 3596 | Non-infected | No Cause Identified | B |
| 3638 | Sepsis | Bacterial Sepsis | B |
| 3645 | Non-infected | No Cause Identified | B |
| 3783 | Non-infected | No Cause Identified | C |
| 3986 | Sepsis | Culture Negative Sepsis | B |
| 4130 | Sepsis | Viral Sepsis | B |
| 4241 | Sepsis | Culture Negative Sepsis | C |
| 4464 | Non-infected | No Cause Identified | B |
| 4638 | Non-infected | No Cause Identified | B |
| 4798 | Sepsis | Viral Sepsis | B |
| 4807 | Sepsis | Culture Negative Sepsis | B |
| 4868 | Sepsis | Viral Sepsis | B |
| 4901 | Sepsis | Culture Negative Sepsis | C |
| 5017 | Non-infected | No Cause Identified | A |
| 5368 | Sepsis | Culture Negative Sepsis | A |
| 5401 | Non-infected | No Cause Identified | C |
| 5522 | Sepsis | Culture Negative Sepsis | B |
| 5565 | Sepsis | Bacterial Sepsis | B |
| 5700 | Sepsis | Viral Sepsis | B |
| 5734 | Sepsis | Viral Sepsis | A |
| 5791 | Non-infected | No Cause Identified | C |
| 5842 | Sepsis | Culture Negative Sepsis | A |
| 5859 | Cardiopulmonary Bypass | Cardiopulmonary Bypass | C |
| 5909 | Non-infected | Asphyxial Injury | C |
| 5931 | Sepsis | Viral Sepsis | C |
| 6126 | Sepsis | Bacterial Sepsis | C |
| 6134 | Non-infected | Neurologic Disease | A |
| 6468 | Sepsis | Culture Negative Sepsis | C |
| 6499 | Sepsis | Bacterial Sepsis | C |
| 6564 | Non-infected | Oncologic Disease | A |

| HASHED PATIENT ID | MODS ETIOLOGY | DETAILED ETIOLOGY | SUBGROUP |
|---|---|---|---|
| 6606 | Sepsis | Viral Sepsis | B |
| 6698 | Non-infected | Toxic Exposure | B |
| 7184 | Non-infected | Neurologic Disease | A |
| 7331 | Sepsis | Culture Negative Sepsis | C |
| 7343 | Non-infected | Cardiac Arrest | C |
| 7411 | Non-infected | Toxic Exposure | C |
| 7433 | Non-infected | Heart Failure | B |
| 7632 | Non-infected | Exacerbation of Chronic Disease | B |
| 7639 | Non-infected | Cardiac Arrest | B |
| 7791 | Trauma | Traumatic Injury | A |
| 7810 | Non-infected | Exacerbation of Chronic Disease | A |
| 7919 | Non-infected | Cardiac Arrest | A |
| 7977 | Trauma | Traumatic Injury | A |
| 8047 | Non-infected | Cardiac Arrest | B |
| 8823 | Non-infected | Cardiac Arrest | C |
| 8825 | Sepsis | Bacterial Sepsis | B |
| 8877 | Non-infected | Pulmonary Embolus | C |
| 8893 | Trauma | Traumatic Injury | A |
| 9129 | Trauma | Traumatic Injury | A |
| 9222 | Non-infected | Neurologic Disease | A |
| 9328 | Non-infected | Postoperative Status | B |
| 9345 | Non-infected | Neurologic Disease | A |
| 9480 | Sepsis | Culture Negative Sepsis | C |
| 9484 | Non-infected | Oncologic Disease | B |
| 9630 | Sepsis | Culture Negative Sepsis | C |
| 9712 | Sepsis | Viral Sepsis | C |
| 9948 | Non-infected | No Cause Identified | C |

## Table S6. Immunocompromised diagnoses by subgroup.

| HASHED PATIENT ID | IMMUNOCOMPROMISED DIAGNOSIS | COMPUTED SUBGROUP | SURVIVAL TO PICU DISCHARGE |
|---|---|---|---|
| 5368 | Cancer, solid tumor | A | Yes |
| 6564 | Cancer, leukemia | A | Yes |
| 1521 | Very early onset inflammatory bowel disease | B | Yes |
| 3596 | Hematopoietic cell transplant | B | Yes |
| 5522 | Cancer, solid tumor | B | No |
| 5565 | Cancer, solid tumor | B | Yes |
| 7433 | Cancer, solid tumor | B | No |
| 9484 | Cancer, leukemia | B | Yes |
| 3227 | Hematopoietic cell transplant | C | Yes |
| 6499 | Common variable immunodeficiency | C | No |
| 9630 | Cancer, solid tumor | C | Yes |

**Table S7. 368 proteins measured in proteomics panel used in IEI cohort.**

*Due to its size, Table S7 is available in the supplemental spreadsheet.*

**Table S8. Leading edge analysis of protein expression in Group C patients compared to HC participants.**

| HALLMARK PATHWAY | FDR ADJUSTED P-VALUE | LEADING EDGE ENRICHMENT |
|---|---|---|
| HALLMARK_IL6_JAK_ STAT3_SIGNALING | 0.004779285 | IL6, CCL7, PLA2G2A, REG1A, JUN, HMOX1, IL4R, TNFRSF12A, TNFRSF1A, TNFRSF1B, CXCL10, CXCL9, IL2RA, IL15RA, IL18R1, LTBR, CSF1, CD38, CXCL11, ACVRL1, IL1B, CD14, TNF, CXCL13, IL12RB1, TGFB1, CSF2RA, FAS, IFNGR1 |
| HALLMARK_XENOBIOTIC_ METABOLISM | 0.005617107 | PTS, FABP1, IGFBP1, FBP1, REG1A, HMOX1, TNFRSF1A, NINJ1, AHCY, CES1, ARG1, DDC, IGFBP4, EPHA2, GSTA3, DCXR |
| HALLMARK_ INFLAMMATORY_ RESPONSE | 0.008012074 | IL6, CCL7, CXCL8, CSF3, CCL20, LIF, TIMP1, IL10, BST2, IL4R, OSM, TNFRSF1B, CXCL10, NAMPT, CXCL9, CCL2, IL15, PLAUR, CLEC5A, LDLR, IL15RA, PVR, IL18R1, ADM, LAMP3, CSF1, MSR1, LYN, CXCL11, IL1B, CX3CL1, SLAMF1, CD14 |
| HALLMARK_INTERFERON_ ALPHA_RESPONSE | 0.016318021 | BST2, IL4R, GBP2, CXCL10, IL15, CASP1, LAMP3, CSF1, CD74, OGFR, PSME2, LAP3, CXCL11 |
| HALLMARK_TNFA_ SIGNALING_VIA_NFKB | 0.030735353 | IL6, AREG, CCL20, LIF, SERPINB8, JUN, CXCL10, NAMPT, FOSB, NINJ1, CCL2, PLAUR, CEBPB, LDLR, IL15RA, TNC, SOD2, CSF1, CCN1, B4GALT1, PTX3, CXCL11, KYNU, IL1B, CCL4, TNF, NFKBIE, VEGFA, TANK, F3, IL18, ICAM1 |
| HALLMARK_INTERFERON_ GAMMA_RESPONSE | 0.031250000 | IL6, CCL7, BST2, IL4R, CXCL10, NAMPT, CXCL9, CCL2, IL15, CASP1, IL15RA, CD274, SOD2, IL18BP, CD38, CD74, OGFR, PSME2, VAMP5, LAP3, CXCL11 |
| HALLMARK_OXIDATIVE_ PHOSPHORYLATION | 0.035126235 | NDUFS6, GRPEL1, ACAA1, POLR2F, BAX, COX5B |
| HALLMARK_UNFOLDED_ PROTEIN_RESPONSE | 0.035144814 | IGFBP1, ZBTB17, EIF4EBP1, CCL2, CEBPB, NPM1, FUS, PREB, BAG3, VEGFA |
| HALLMARK_EPITHELIAL_ MESENCHYMAL_ TRANSITION | 0.038038038 | IL6, AREG, CXCL8, SDC1, PRSS2, TIMP1, JUN, FSTL3, TNFRSF12A, TFPI2, SFRP1, TNFRSF11B, THBS2, MMP3, IL15, ACTA2, TGM2, PLAUR, COL4A1, TNC, BGN, PVR, CAPG, IGFBP4, VCAN, IGFBP2 |
| HALLMARK_ESTROGEN_ RESPONSE_LATE | 0.044102564 | AREG, KRT19, AGR2, TFPI2, SORD, FKBP4, MDK, TFF1, IGFBP4, CA12, DCXR, SLC16A1, TFF3, BLVRB, MAPT, FABP5, FKBP5 |

**Table S9. Sensitivity analysis of corticosteroid effects on IL-6/JAK/STAT3 signaling by cell type.**

| CELL SUBSET | MEDIAN MODULE ENRICHMENT SCORE | | CLIFF'S DELTA (δ) | EFFECT MAGNITUDE |
|---|---|---|---|---|
| | NO STEROIDS (N=5) | YES STEROIDS (N=4) | | |
| Naive $CD8^+$ T cells | -0.00326 | 0.005823 | -0.11232 | negligible |
| $CD8^+$ CM cells | -0.00572 | 0.008703 | -0.23267 | small |
| $CD8^+$ EM cells | 0.020369 | 0.027366 | -0.09088 | negligible |
| $CD8^+$ EMRA cells | 0.009158 | 0.003892 | 0.06261 | negligible |
| Naive $CD4^+$ T cells | 0.018118 | 0.028621 | -0.10488 | negligible |
| Effector $CD4^+$ T cells | 0.03926 | 0.044894 | -0.05697 | negligible |
| $TCR\gamma\delta^+$ T cells | 0.040752 | 0.035835 | 0.056015 | negligible |
| NK-T cells | 0.006283 | 0.022906 | -0.17376 | small |
| NK cells | 0.019151 | 0.028349 | -0.10808 | negligible |
| Naive B cells | 0.021713 | 0.00994 | 0.13029 | negligible |
| Immature B cells | 0.046097 | 0.041441 | 0.05601 | negligible |
| Memory B cells | 0.020756 | -0.00845 | 0.293559 | small |
| Plasma cells | 0.022026 | 0.01702 | 0.04128 | negligible |
| Classical Monocytes | 0.122681 | 0.038143 | 0.423969 | medium |
| Non-classical Monocytes | 0.091807 | 0.077438 | 0.096315 | negligible |

Cliff's Delta (δ) is a non-parametric effect size measure quantifying the difference between two distributions. We interpreted effect magnitudes using standard thresholds (26): negligible ($|\delta| < 0.15$), small ($0.15 \leq |\delta| < 0.33$), medium ($0.33 \leq |\delta| < 0.47$), and large ($|\delta| \geq 0.47$). A positive δ indicates higher scores in the "No Steroids" group (consistent with steroid-mediated suppression), while a negative δ indicates higher scores in the "Yes Steroids" group (consistent with a lack of response to corticosteroids).

## Table S10. Antibodies used in 13-marker T cell phosphoflow cytometry panel.

| MARKER | FLUORORPHORE | VENDOR | CLONE | CATALOG NO. |
|---|---|---|---|---|
| CD4 | BUV395 | BD | RPA-T4 | 564724 |
| Live/Dead Aqua | V500 | Thermo Scientific | N/A | L34966 |
| CD14 | V500 | BD | M5E2 | 561391 |
| CD16 | V500 | BD | 3G8 | 561394 |
| CD19 | V500 | BD | HIB19 | 561121 |
| CD8a | BV605 | BioLegend | RPA-T8 | 301040 |
| CD45RA | BV650 | BD | HI100 | 563963 |
| CD27 | BV785 | BioLegend | O323 | 302832 |
| pSTAT1 | AF488 | BD | 4A | 612596 |
| pSTAT3 | PE | BD | 4/P STAT3 | 612569 |
| pSTAT5 | PE-Cy7 | BD | 47 | 560117 |
| Total STAT3 | APC | BD | M59-50 | 560392 |
| CD3 | APC-R700 | BD | UCHT1 | 565119 |

**Table S11. Modified Proulx criteria used for screening and enrollment in the MODS cohort.**

| ORGAN SYSTEM | CLINICAL AND LABORATORY CRITERIA FOR ORGAN DYSFUNCTION[1] |
|---|---|
| Respiratory Dysfunction | i) Respiratory rate > 90 breaths per min (age < 1yr) or > 70 breaths per min (age ≥ 1yr) **OR**<br>ii) PaO2 < 40 mmHg (5.3kPa) in absence of cyanotic congenital heart disease **OR**<br>iii) PaCO2 ≥ 65 mmHg (8.7kPa) **OR**<br>iv) PaO2/FiO2 < 200 in absence of cyanotic congenital heart disease (where P/F ratio is based on simultaneous measurements) **OR**<br>v) Mechanical ventilation<br>(1) Must be for > 24hrs post-operative if intubated just for surgery/procedure<br>(2) Includes new HFNC ≥ 6L/min, non-invasive ventilation (e.g. CPAP, BiPAP), and VV ECMO<br>(3) Includes patients with tracheostomy who do NOT require mechanical ventilation at baseline<br>(4) For subjects with non-invasive ventilation at baseline, intubation would qualify as Respiratory Dysfunction |
| Cardiovascular Dysfunction | i) Systolic blood pressure < 40 mmHg (age < 1yr) or < 50 mmHg (age ≥ 1yr) **OR**<br>ii) Heart rate < 50 or > 220 (age < 1yr), < 40 or > 200 (age ≥1yr) **OR**<br>iii) Cardiac arrest **OR**<br>iv) pH < 7.2 with normal PaCO2 **OR**<br>v) Continuous vasoactive drug infusion for hemodynamic support (excluding dopamine infusion ≤5ug/kg/min) **OR**<br>vi) VA ECMO **OR**<br>vii) Ventricular assist device |
| Hematologic Dysfunction | i) Hemoglobin < 5g/dL (50g/L) **OR**<br>ii) White blood cell count < 3,000/mm3 (3x10*9/L) **OR**<br>iii) Platelet count < 20,000/mm3 (20x10*9) **OR**<br>iv) PT > 20 seconds OR aPTT > 60 seconds (in absence of anticoagulation therapy) |
| Gastrointestinal Dysfunction | i) Gastrointestinal bleeding **AND** one of the following believed to be the result of gastroduodenal bleeding:<br>(1) Drop in hemoglobin ≥ 2g/dL (20g/L) over 24 hours **OR**<br>(2) Blood transfusion **OR**<br>(3) Hypotension with blood pressure < 5th percentile of age **OR**<br>(4) Gastric or duodenal surgery |
| Renal Dysfunction | i) Serum BUN ≥ 100mg/dL (36 mmol/L) **OR**<br>ii) Serum creatinine ≥ 2 mg/dL (177 umol/L) without pre-existing renal disease **OR**<br>iii) Dialysis and/or hemofiltration |
| Neurological Dysfunction | i) Fixed, dilated pupils **OR**<br>ii) Glasgow coma score (GCS) < 5 in the absence of neuromuscular blocking drugs |
| Hepatic Dysfunction | i) Total bilirubin > 3mg/dL (60 umol/L) |

- [1] MODS criteria derived from Proulx *et al*, (27).

**Table S12. Age range and sex for each healthy control participant.**

| HASHED PARTICIPANT ID | AGE RANGE (YEARS) | SEX |
|---|---|---|
| HC-105 | 6-10 | M |
| HC-175 | 11-15 | M |
| HC-182 | 0-5 | F |
| HC-304 | 6-10 | M |
| HC-335 | 11-15 | M |
| HC-411 | 11-15 | M |
| HC-417 | 16-20 | M |
| HC-439 | 11-15 | M |
| HC-500 | 6-10 | M |
| HC-534 | 11-15 | F |
| HC-549 | 0-5 | F |
| HC-581 | 11-15 | F |
| HC-598 | 11-15 | F |
| HC-665 | 16-20 | M |
| HC-679 | 6-10 | F |
| HC-698 | 6-10 | F |
| HC-730 | 6-10 | M |
| HC-746 | 6-10 | F |
| HC-778 | 6-10 | M |
| HC-782 | 0-5 | F |
| HC-841 | 0-5 | F |
| HC-919 | 16-20 | M |
| HC-937 | 0-5 | M |
| HC-963 | 11-15 | F |
| HC-979 | 6-10 | M |

Median age of HC cohort: 10 years.

**Table S13. Final set of 1448 proteins which met quality control thresholds in all three experiments and were included in downstream analyses.**

*Due to its size, Table S13 is available in the supplemental spreadsheet.*

## Table S14. Gene sets used in GSEA and GSVA analysis.

| GENE SET | GENE SYMBOLS |
|---|---|
| HALLMARK_ TNFA_ SIGNALING_ VIA_NFKB | ABCA1, ACKR3, AREG, ATF3, ATP2B1, B4GALT1, B4GALT5, BCL2A1, BCL3, BCL6, BHLHE40, BIRC2, BIRC3, BMP2, BTG1, BTG2, BTG3, CCL2, CCL20, CCL4, CCL5, CCN1, CCND1, CCNL1, CCRL2, CD44, CD69, CD80, CD83, CDKN1A, CEBPB, CEBPD, CFLAR, CLCF1, CSF1, CSF2, CXCL1, CXCL10, CXCL11, CXCL2, CXCL3, CXCL6, DENND5A, DNAJB4, DRAM1, DUSP1, DUSP2, DUSP4, DUSP5, EDN1, EFNA1, EGR1, EGR2, EGR3, EHD1, EIF1, ETS2, F2RL1, F3, FJX1, FOS, FOSB, FOSL1, FOSL2, FUT4, G0S2, GADD45A, GADD45B, GCH1, GEM, GFPT2, GPR183, HBEGF, HES1, ICAM1, ICOSLG, ID2, IER2, IER3, IER5, IFIH1, IFIT2, IFNGR2, IL12B, IL15RA, IL18, IL1A, IL1B, IL23A, IL6, IL6ST, IL7R, INHBA, IRF1, IRS2, JAG1, JUN, JUNB, KDM6B, KLF10, KLF2, KLF4, KLF6, KLF9, KYNU, LAMB3, LDLR, LIF, LITAF, MAFF, MAP2K3, MAP3K8, MARCKS, MCL1, MSC, MXD1, MYC, NAMPT, NFAT5, NFE2L2, NFIL3, NFKB1, NFKB2, NFKBIA, NFKBIE, NINJ1, NR4A1, NR4A2, NR4A3, OLR1, PANX1, PDE4B, PDLIM5, PER1, PFKFB3, PHLDA1, PHLDA2, PLAU, PLAUR, PLEK, PLK2, PLPP3, PMEPA1, PNRC1, PPP1R15A, PTGER4, PTGS2, PTPRE, PTX3, RCAN1, REL, RELA, RELB, RHOB, RIGI, RIPK2, RNF19B, SAT1, SDC4, SERPINB2, SERPINB8, SERPINE1, SGK1, SIK1, SLC16A6, SLC2A3, SLC2A6, SMAD3, SNN, SOCS3, SOD2, SPHK1, SPSB1, SQSTM1, STAT5A, TANK, TAP1, TGIF1, TIPARP, TLR2, TNC, TNF, TNFAIP2, TNFAIP3, TNFAIP6, TNFAIP8, TNFRSF9, TNFSF9, TNIP1, TNIP2, TRAF1, TRIB1, TRIP10, TSC22D1, TUBB2A, VEGFA, YRDC, ZBTB10, ZC3H12A, ZFP36 |
| HALLMARK_ IL6_ JAK_STAT3_ SIGNALING | A2M, ACVR1B, ACVRL1, BAK1, CBL, CCL7, CCR1, CD14, CD36, CD38, CD44, CD9, CNTFR, CRLF2, CSF1, CSF2, CSF2RA, CSF2RB, CSF3R, CXCL1, CXCL10, CXCL11, CXCL13, CXCL3, CXCL9, DNTT, EBI3, FAS, GRB2, HAX1, HMOX1, IFNAR1, IFNGR1, IFNGR2, IL10RB, IL12RB1, IL13RA1, IL15RA, IL17RA, IL17RB, IL18R1, IL1B, IL1R1, IL1R2, IL2RA, IL2RG, IL3RA, IL4R, IL6, IL6ST, IL7, IL9R, INHBE, IRF1, IRF9, ITGA4, ITGB3, JUN, LEPR, LTB, LTBR, MAP3K8, MYD88, OSMR, PDGFC, PF4, PIK3R5, PIM1, PLA2G2A, PTPN1, PTPN11, PTPN2, REG1A, SOCS1, SOCS3, STAM2, STAT1, STAT2, STAT3, TGFB1, TLR2, TNF, TNFRSF12A, TNFRSF1A, TNFRSF1B, TNFRSF21, TYK2 |
| HALLMARK_ IL2_STAT5_ SIGNALING | ABCB1, ADAM19, AGER, AHCY, AHNAK, AHR, ALCAM, AMACR, ANXA4, APLP1, ARL4A, BATF, BATF3, BCL2, BCL2L1, BHLHE40, BMP2, BMPR2, CA2, CAPG, CAPN3, CASP3, CCND2, CCND3, CCNE1, CCR4, CD44, CD48, CD79B, CD81, CD83, CD86, CDC42SE2, CDC6, CDCP1, CDKN1C, CISH, CKAP4, COCH, COL6A1, CSF1, CSF2, CST7, CTLA4, CTSZ, CXCL10, CYFIP1, DCPS, DENND5A, DHRS3, DRC1, ECM1, EEF1AKMT1, EMP1, ENO3, ENPP1, EOMES, ETFBKMT, ETV4, F2RL2, FAH, FGL2, FLT3LG, FURIN, GABARAPL1, GADD45B, GALM, GATA1, GBP4, GLIPR2, GPR65, GPR83, GPX4, GSTO1, GUCY1B1, HIPK2, HK2, HOPX, HUWE1, HYCC2, ICOS, IFITM3, IFNGR1, IGF1R, IGF2R, IKZF2, IKZF4, IL10, IL10RA, IL13, IL18R1, IL1R2, IL1RL1, IL2RA, IL2RB, IL3RA, IL4R, IRF4, IRF6, IRF8, ITGA6, ITGAE, ITGAV, ITIH5, KLF6, LCLAT1, LIF, LRIG1, LRRC8C, LTB, MAFF, MAP3K8, MAP6, MAPKAPK2, MUC1, MXD1, MYC, MYO1C, MYO1E, NCOA3, NCS1, NDRG1, NFIL3, NFKBIZ, NOP2, NRP1, NT5E, ODC1, P2RX4, P4HA1, PDCD2L, PENK, PHLDA1, PHTF2, PIM1, PLAGL1, PLEC, PLIN2, PLPP1, PLSCR1, PNP, POU2F1, PRAF2, PRKCH, PRNP, PTCH1, PTGER2, PTH1R, PTRH2, PUS1, RABGAP1L, RGS16, RHOB, RHOH, RNH1, RORA, RRAGD, S100A1, SCN9A, SELL, SELP, SERPINB6, SERPINC1, SH3BGRL2, SHE, SLC1A5, SLC29A2, SLC2A3, SLC39A8, SMPDL3A, SNX14, SNX9, SOCS1, SOCS2, SPP1, SPRED2, SPRY4, ST3GAL4, SWAP70, SYNGR2, SYT11, TGM2, TIAM1, TLR7, TNFRSF18, TNFRSF1B, TNFRSF21, TNFRSF4, TNFRSF8, TNFRSF9, TNFSF10, TNFSF11, TRAF1, TTC39B, TWSG1, UCK2, UMPS, WLS, XBP1 |
| HALLMARK_ INTERFERON_ GAMMA_ RESPONSE | ADAR, APOL6, ARID5B, ARL4A, AUTS2, B2M, BANK1, BATF2, BPGM, BST2, BTG1, C1R, C1S, CASP1, CASP3, CASP4, CASP7, CASP8, CCL2, CCL5, CCL7, CD274, CD38, CD40, CD69, CD74, CD86, CDKN1A, CFB, CFH, CIITA, CMKLR1, CMPK2, CMTR1, CSF2RB, CXCL10, CXCL11, CXCL9, DDX60, DHX58, EIF2AK2, EIF4E3, EPSTI1, FAS, FCGR1A, FGL2, FPR1, GBP4, GBP6, GCH1, GPR18, GZMA, HELZ2, HERC6, HIF1A, HLA-A, HLA-B, HLA-DMA, HLA-DQA1, HLA-DRB1, HLA-G, ICAM1, IDO1, IFI27, IFI30, IFI35, IFI44, IFI44L, IFIH1, IFIT1, IFIT2, IFIT3, IFITM2, IFITM3, IFNAR2, IL10RA, IL15, IL15RA, IL18BP, IL2RB, IL4R, IL6, IL7, IRF1, IRF2, IRF4, IRF5, IRF7, IRF8, IRF9, ISG15, ISG20, ISOC1, ITGB7, JAK2, KLRK1, LAP3, LATS2, LCP2, LGALS3BP, LY6E, LYSMD2, MARCHF1, METTL7B, MT2A, MTHFD2, MVP, MX1, MX2, MYD88, NAMPT, NCOA3, NFKB1, NFKBIA, NLRC5, NMI, NOD1, NUP93, OAS2, OAS3, OASL, OGFR, P2RY14, PARP12, PARP14, PDE4B, PELI1, PFKP, PIM1, PLA2G4A, PLSCR1, PML, PNP, PNPT1, PSMA2, PSMA3, PSMB10, PSMB2, PSMB8, PSMB9, PSME1, PSME2, PTGS2, PTPN1, PTPN2, PTPN6, RAPGEF6, RBCK1, RIGI, RIPK1, RIPK2, RNF213, RNF31, RSAD2, RTP4, SAMD9L, SAMHD1, SECTM1, SELP, SERPING1, SLAMF7, SLC25A28, SOCS1, SOCS3, SOD2, SP110, SPPL2A, SRI, SSPN, ST3GAL5, ST8SIA4, STAT1, STAT2, STAT3, STAT4, TAP1, TAPBP, TDRD7, TNFAIP2, TNFAIP3, TNFAIP6, TNFSF10, TOR1B, TRAFD1, TRIM14, TRIM21, TRIM25, TRIM26, TXNIP, UBE2L6, UPP1, USP18, VAMP5, VAMP8, VCAM1, WARS1, XAF1, XCL1, ZBP1, ZNFX1 |
| HALLMARK_ PI3K_AKT_ MTOR_ SIGNALING | ACACA, ACTR2, ACTR3, ADCY2, AKT1, AKT1S1, AP2M1, ARF1, ARHGDIA, ARPC3, ATF1, CAB39, CAB39L, CALR, CAMK4, CDK1, CDK2, CDK4, CDKN1A, CDKN1B, CFL1, CLTC, CSNK2B, CXCR4, DAPP1, DDIT3, DUSP3, E2F1, ECSIT, EGFR, EIF4E, FASLG, FGF17, FGF22, FGF6, GNA14, GNGT1, GRB2, GRK2, GSK3B, HRAS, HSP90B1, IL2RG, IL4, IRAK4, ITPR2, LCK, MAP2K3, MAP2K6, MAP3K7, MAPK1, MAPK10, MAPK8, MAPK9, MAPKAP1, MKNK1, MKNK2, MYD88, NCK1, NFKBIB, NGF, NOD1, PAK4, PDK1, PFN1, PIK3R3, PIKFYVE, PIN1, PITX2, PLA2G12A, PLCB1, PLCG1, PPP1CA, PPP2R1B, PRKAA2, PRKAG1, PRKAR2A, PRKCB, PTEN, PTPN11, RAC1, RAF1, RALB, RIPK1, RIT1, RPS6KA1, RPS6KA3, RPTOR, SFN, SLA, SLC2A1, |

| GENE SET | GENE SYMBOLS |
|---|---|
| | SMAD2, SQSTM1, STAT2, TBK1, THEM4, TIAM1, TNFRSF1A, TRAF2, TRIB3, TSC2, UBE2D3, UBE2N, VAV3, YWHAB |
| HALLMARK_ OXIDATIVE_ PHOSPHOR YLATION | ABCB7, ACAA1, ACAA2, ACADM, ACADSB, ACADVL, ACAT1, ACO2, AFG3L2, AIFM1, ALAS1, ALDH6A1, ATP1B1, ATP5F1A, ATP5F1B, ATP5F1C, ATP5F1D, ATP5F1E, ATP5MC1, ATP5MC2, ATP5MC3, ATP5ME, ATP5MF, ATP5MG, ATP5PB, ATP5PD, ATP5PF, ATP5PO, ATP6AP1, ATP6V0B, ATP6V0C, ATP6V0E1, ATP6V1C1, ATP6V1D, ATP6V1E1, ATP6V1F, ATP6V1G1, ATP6V1H, BAX, BCKDHA, BDH2, CASP7, COX10, COX11, COX15, COX17, COX4I1, COX5A, COX5B, COX6A1, COX6B1, COX6C, COX7A2, COX7A2L, COX7B, COX7C, COX8A, CPT1A, CS, CYB5A, CYB5R3, CYC1, CYCS, DECR1, DLAT, DLD, DLST, ECH1, ECHS1, ECI1, ETFA, ETFB, ETFDH, FDX1, FH, FXN, GLUD1, GOT2, GPI, GPX4, GRPEL1, HADHA, HADHB, HCCS, HSD17B10, HSPA9, HTRA2, IDH1, IDH2, IDH3A, IDH3B, IDH3G, IMMT, ISCA1, ISCU, LDHA, LDHB, LRPPRC, MAOB, MDH1, MDH2, MFN2, MGST3, MPC1, MRPL11, MRPL15, MRPL34, MRPL35, MRPS11, MRPS12, MRPS15, MRPS22, MRPS30, MTRF1, MTRR, MTX2, NDUFA1, NDUFA2, NDUFA3, NDUFA4, NDUFA5, NDUFA6, NDUFA7, NDUFA8, NDUFA9, NDUFAB1, NDUFB1, NDUFB2, NDUFB3, NDUFB4, NDUFB5, NDUFB6, NDUFB7, NDUFB8, NDUFC1, NDUFC2, NDUFS1, NDUFS2, NDUFS3, NDUFS4, NDUFS6, NDUFS7, NDUFS8, NDUFV1, NDUFV2, NNT, NQO2, OAT, OGDH, OPA1, OXA1L, PDHA1, PDHB, PDHX, PDK4, PDP1, PHB2, PHYH, PMPCA, POLR2F, POR, PRDX3, RETSAT, RHOT1, RHOT2, SDHA, SDHB, SDHC, SDHD, SLC25A11, SLC25A12, SLC25A20, SLC25A3, SLC25A4, SLC25A5, SLC25A6, SUCLA2, SUCLG1, SUPV3L1, SURF1, TCIRG1, TIMM10, TIMM13, TIMM17A, TIMM50, TIMM8B, TIMM9, TOMM22, TOMM70, UQCR10, UQCR11, UQCRB, UQCRC1, UQCRC2, UQCRFS1, UQCRH, UQCRQ, VDAC1, VDAC2, VDAC3 |
| HALLMARK_ GLYCOLYSIS | ABCB6, ADORA2B, AGL, AGRN, AK3, AK4, AKR1A1, ALDH7A1, ALDH9A1, ALDOA, ALDOB, ALG1, ANG, ANGPTL4, ANKZF1, ARPP19, ARTN, AURKA, B3GALT6, B3GAT1, B3GAT3, B3GNT3, B4GALT1, B4GALT2, B4GALT4, B4GALT7, BIK, BPNT1, CACNA1H, CAPN5, CASP6, CD44, CDK1, CENPA, CHPF, CHPF2, CHST1, CHST12, CHST2, CHST4, CHST6, CITED2, CLDN3, CLDN9, CLN6, COG2, COL5A1, COPB2, CTH, CXCR4, CYB5A, DCN, DDIT4, DEPDC1, DLD, DPYSL4, DSC2, ECD, EFNA3, EGFR, EGLN3, ELF3, ENO1, ENO2, ERO1A, EXT1, EXT2, FAM162A, FBP2, FKBP4, FUT8, G6PD, GAL3ST1, GALE, GALK1, GALK2, GAPDHS, GCLC, GFPT1, GFUS, GLCE, GLRX, GMPPA, GMPPB, GNE, GNPDA1, GOT1, GOT2, GPC1, GPC3, GPC4, GPR87, GUSB, GYS1, GYS2, HAX1, HDLBP, HK2, HMMR, HOMER1, HS2ST1, HS6ST2, HSPA5, IDH1, IDUA, IER3, IGFBP3, IL13RA1, IRS2, ISG20, KDELR3, KIF20A, KIF2A, LCT, LDHA, LDHC, LHPP, LHX9, MDH1, MDH2, ME1, ME2, MED24, MERTK, MET, MIF, MIOX, MPI, MXI1, NANP, NASP, NDST3, NDUFV3, NOL3, NSDHL, NT5E, P4HA1, P4HA2, PAM, PAXIP1, PC, PDK3, PFKFB1, PFKP, PGAM1, PGAM2, PGK1, PGLS, PGM2, PHKA2, PKM, PKP2, PLOD1, PLOD2, PMM2, POLR3K, PPFIA4, PPIA, PPP2CB, PRPS1, PSMC4, PYGB, PYGL, QSOX1, RARS1, RBCK1, RPE, RRAGD, SAP30, SDC1, SDC2, SDC3, SDHC, SLC16A3, SLC25A10, SLC25A13, SLC35A3, SLC37A4, SOD1, SOX9, SPAG4, SRD5A3, STC1, STC2, STMN1, TALDO1, TFF3, TGFA, TGFBI, TKTL1, TPBG, TPI1, TPST1, TXN, UGP2, VCAN, VEGFA, VLDLR, XYLT2, ZNF292 |
| KEGG_ GLYCOLYSIS_ GLUCONEO GENESIS | ACSS1, ACSS2, ADH1A, ADH1B, ADH1C, ADH4, ADH5, ADH6, ADH7, AKR1A1, ALDH1A3, ALDH1B1, ALDH2, ALDH3A1, ALDH3A2, ALDH3B1, ALDH3B2, ALDH7A1, ALDH9A1, ALDOA, ALDOB, ALDOC, BPGM, DLAT, DLD, ENO1, ENO2, ENO3, FBP1, FBP2, G6PC1, G6PC2, GALM, GAPDH, GCK, GPI, HK1, HK2, HK3, LDHA, LDHAL6A, LDHAL6B, LDHB, LDHC, PCK1, PCK2, PDHA1, PDHA2, PDHB, PFKL, PFKM, PFKP, PGAM1, PGAM2, PGAM4, PGK1, PGK2, PGM1, PGM2, PKLR, PKM, TPI1 |
| KEGG_ OXIDATIVE_ PHOSPHOR YLATION | ATP12A, ATP4A, ATP4B, ATP5F1A, ATP5F1B, ATP5F1C, ATP5F1D, ATP5F1E, ATP5MC1, ATP5MC1P5, ATP5MC2, ATP5MC3, ATP5ME, ATP5MF, ATP5MG, ATP5PB, ATP5PD, ATP5PF, ATP5PO, ATP6AP1, ATP6V0A1, ATP6V0A2, ATP6V0A4, ATP6V0B, ATP6V0C, ATP6V0D1, ATP6V0D2, ATP6V0E1, ATP6V0E2, ATP6V1A, ATP6V1B1, ATP6V1B2, ATP6V1C1, ATP6V1C2, ATP6V1D, ATP6V1E1, ATP6V1E2, ATP6V1F, ATP6V1G1, ATP6V1G2, ATP6V1G3, ATP6V1H, COX10, COX11, COX15, COX17, COX4I1, COX4I2, COX5A, COX5B, COX6A1, COX6A2, COX6B1, COX6B2, COX6C, COX6CP3, COX7A1, COX7A2, COX7A2L, COX7B, COX7B2, COX7C, COX8A, COX8C, CYC1, LHPP, MT-ATP6, MT-ATP8, MT-CO1, MT-CO2, MT-CO3, MT-CYB, MT-ND1, MT-ND2, MT-ND3, MT-ND4, MT-ND4L, MT-ND5, MT-ND6, NDUFA1, NDUFA10, NDUFA11, NDUFA2, NDUFA3, NDUFA4, NDUFA4L2, NDUFA5, NDUFA6, NDUFA7, NDUFA8, NDUFA9, NDUFAB1, NDUFB1, NDUFB10, NDUFB2, NDUFB3, NDUFB4, NDUFB5, NDUFB6, NDUFB7, NDUFB8, NDUFB9, NDUFC1, NDUFC2, NDUFS1, NDUFS2, NDUFS3, NDUFS4, NDUFS5, NDUFS6, NDUFS7, NDUFS8, NDUFV1, NDUFV2, NDUFV3, PPA1, PPA2, SDHA, SDHB, SDHC, SDHD, TCIRG1, UQCR10, UQCR10P1, UQCR11, UQCRB, UQCRC1, UQCRC2, UQCRFS1, UQCRH, UQCRHL, UQCRQ |
| KEGG_ MTOR_ SIGNALING_ PATHWAY | AKT1, AKT2, AKT3, BRAF, CAB39, CAB39L, DDIT4, EIF4B, EIF4E, EIF4E1B, EIF4E2, EIF4EBP1, HIF1A, IGF1, INS, MAPK1, MAPK3, MLST8, MTOR, PDPK1, PGF, PIK3CA, PIK3CB, PIK3CD, PIK3CG, PIK3R1, PIK3R2, PIK3R3, PIK3R5, PRKAA1, PRKAA2, RHEB, RICTOR, RPS6, RPS6KA1, RPS6KA2, RPS6KA3, RPS6KA6, RPS6KB1, RPS6KB2, RPTOR, STK11, STRADA, TSC1, TSC2, ULK1, ULK2, ULK3, VEGFA, VEGFB, VEGFC, VEGFD |
| SUBSPACE_ LYMPHOID_ PROTECTIVE | ARL14EP, BPGM, BTN3A2, BUB3, CAMK4, CASP8, CCNB1IP1, CD247, CD3E, CD3G, DBT, DDX6, DYRK2, JAK1, KLRB1, MAP4K1, NCR3, PIK3R1, PLCG1, PPP2R5C, SEMA4F, SIDT1, SMAD4, SMYD2, TP53BP1, TRIB2, ZAP70, ZCCHC4, ZNF831 |

## Table S15. Gene sets used in Immune Dictionary cytokine signature analysis.

| GENE SET | GENE SYMBOLS |
|---|---|
| IMMUNE_ DICTIONARY_ CD8_IL1B | SOCS3, BCL3, GADD45G, IGFBP4, GZMB, CRIP1, SOCS1, ARID5B, EMP3, SERPINB1, STAT3, SSH2, IFNGR1, GZMA, SBNO2, RGS10, ARID5A, FRMD4B, CYSLTR2, FAM241A, VARS, RGCC, NDRG3, HSD11B1, MXD1, DAPL1, CABLES1, ELOVL6, CARS, PLAC8, SKAP2, GBP2, C15orf48, FAM46A, ZBP1, AARS, MTHFD2, GPR171, CDKN2D, GPR18, NEB, RASGRP2, PIM1, FAM78A, FLOT1, THEMIS, ETV6, FAM102A, PPA1, PRELID2, ADGRE5, SMPDL3A, NSG2, ZNF281, BATF, BIN1, PPM1H, PJA1, ARL5C, ID3, CLIC4, RAMP1, CEBPB, KLHL6, TDRP, NARS, CD226, STAP1, SEMA4D, CRLF2, MAP3K8, SERPINB9, CCR9, MAPKAPK2, PPP1R16B, ICAM1, TREML2, CHST11, TSPAN32, RASGRP1, RRAS2, FKBP5, SLC41A1, CD96, SETBP1, USP6NL, GNGT2, RPAIN, S100A9, TRIB3, ITGAE, S100A8, LAMP2, YARS, IARS, LPP, RNF19A, MFSD6, DTX3L, C5orf30, SLC30A4, KIF1B, SEMA7A, ATP2B4, JAK3, SLC7A1, ACTN2, LARS, RCN3, RAB37, AHR, IDH2, SLC7A5, CLTB, MYD88, BCL2L11, PIM2, SPEF2, ABI3, SLC1A4, KIF23, CREM, TIMP2, ZFP1, LILRB4, SHMT2, GGT1, AKT2, SLPI, IRF8, ABCA2, CD55, CNDP2, TTC39B, RAB27A, TEX2, PDCD1, CKS2, MAP3K5, XPOT, GBP5, CPNE3, TRNP1, TESC, CXXC5, EPOP, MLXIP, EMC9, ANKRD28, RAPGEF3, RRP1B, GPR68, BTBD11, KCNA2, RRAGD, ALDH18A1, GPR146, PLEKHO1, HSPBAP1, LYPD6B, PLSCR1, MEX3A, CCR5, EIF4EBP1, GPRIN3, ACSS2, HSPA1A, HEXB, SLC15A2, TNIP2, AUTS2, BIRC3, MUC1, GADD45B, KCTD12, DBP, ABCB1, ODC1, GALNT10, ZC3H12D, MYO1F, TRPS1, RNF157, CEP164, KSR1, NTRK3, FGFR1, PSAT1, IL12RB2, CSRP1, SERPINI1, CCL17, FAM184A, IER3, FAS, EPHB2, AGFG2, SNX10, DGAT1, DLG3, JCHAIN, MUS81, BAHD1, RUNX2, IFITM10, ART4, FCGRT, SMPD5, HIST1H1D, METRNL, FRY, SLC15A1, CIART |
| IMMUNE_ DICTIONARY_ CD8_IL33 | CD79A, HSPA1A, BCL3, DHX58, FCMR, MS4A1, EBF1, SERPINB1 |
| IMMUNE_ DICTIONARY_ CD8_IL36A | PPA1, BCL3, ZBP1, ISG15, BST2, GBP2, EIF5A, GZMB, PSME2, ISG20, GADD45G, RNF213, EIF2S2, NARS, SHMT2, MTHFD2, NCL, RAN, GBP6, PHGDH, PA2G4, RTP4, SOCS3, ERH, RANBP1, CLIC4, CRIP1, STAT1, IGFBP4, CARS, XAF1, PSAT1, S100A10, BATF, CYCS, GBP6, EPRS, SOCS1, AARS, CCT3, UBA52, SRM, IFIT3, PARP14, IFI35, ST6GALNAC4, OAS3, HSP90AA1, VARS, IRF7, STAT3, NOP56, CEBPB, GADD45B, IFIT1B, GBP7, NOP58, LARS, HSPD1, PHB, EIF3C, LGALS3BP, CD82, SNRPD1, MAPKAPK2, TIMM8A, POLR2L, DDX39A, PCBP1, GBP6, RGS10, EMP3, KSR1, ODC1, C1QBP, GZMA, UCHL3, PARP9, TARS, MRTO4, LARP1, HDGF, MDN1, YARS, IMPDH2, STAT2, MYBBP1A, CHMP4B, IARS, SRSF7, ETV6, GARS, TAPBPL, DKC1, MARS, C15orf48, IRF8, EBNA1BP2, PRMT1, RRP1B, HSPA9, PSMA6, ZNF593, NMI, SARS, LAP3, NOLC1, TOMM40, IL7R, IFRD2, EIF4EBP1, RCC2, OAS1, LSP1, EIF1AX, SLC29A1, SLC7A1, TRAFD1, NAMPT, DHX58, RUVBL1, FAM162A, SNRPA1, WDR12, KLRD1, PRPF31, GBP5, NSUN2, IFITM3, ATAD3A, CALHM6, SLC7A5, NAA20, NOP16, KARS, GGT1, GBP6, MRPL17, ACP5, APEX1, OAS2, LMNA, FRMD4B, WARS, YPEL3, DDIT3, RUVBL2, ALDH18A1, MPP6, DCTPP1, PDCD4, SHMT1, CLTB, DTX3L, TRIB3, NOP2, TNFRSF9, EPOP, RRS1, AIMP2, IPO5, PPP1R16B, YBX3, XPOT, RASGRP2, GSTP1, NOP14, ARID5B, TIMM50, CD274, CKS2, AEN, NME2, ICAM1, USP18, PIM1, EIF2AK2, BIRC3, SLC19A1, IPO4, GALK1, C7orf50, HELZ2, HERC6, FAM136A, PUSL1, MTHFD1, TECPR1, KLHDC4, MUC1, FABP5, RRP9, LIPG, PIM2, KIAA0040, LAMP2, SKAP2, CXCL10, NFKBIA, POLR1A, DDX1, DAXX, NFKB2, AK6, SBNO2, CDK6, PLA2G12A, BYSL, PML, ZNF281, UMPS, TMA16, ITGB7, HIRIP3, GCSH, OR2V1, CD86, CLUH, RCC1, PITRM1, ANKRD44, PHF11, PABPC4, PLSCR3, IFIT3, STAT5A, MOV10, PYCARD, CASTOR1, LAD1, TNFRSF25, TMEM238, MECR, MTHFD1L, PISD, SLFN5, WDR4, POLE3, CAD, ADCY7, ADGRE5, TMEM97, RELB, DENND5A, IL18BP, SMPDL3A, PUS7, ZFP36L2, EEF1AKMT4, IFIH1, NOL10, SMYD2, THOP1, PARP12, PCGF6, HIF1A, MXD4, ADD3, IRGM, OASL, MACF1, CISH, NEFH, BCAT1, TFDP1, ARL5C, BIN1, ASNS, ACTN1, APP, RANGRF, GNG12, HSD11B1, IL2RA, PITPNC1, FEN1, TSC22D3, KIF21B, CDCA7, DOCK9, PNPT1, SLC30A4, HMGA1, GBP4, WDR36, RMI2, BNIP3L, CLPB, RMDN3, GBP6, TXNRD3, CTPS1, POP1, SESN2, FAM46A, POLR1B, TEX2, IFNG, PCK2, SLC25A33, ARHGEF18, SLC6A9, SLC1A4, DAP, JAKMIP1, ITGA4, TOP1MT, PHF10, HSH2D, SMIM13, MAP3K8, TSEN54, SLC7A6, CD96, DCTD, KLF3, PDF, SELENOP, SERPINB1, MS4A6A, TNIK, MID1IP1, POLR3H, FAM78A, CCR9, NSG2, PLEC, RAMP1, PLCG2, TDRP, USP6NL, TADA2A, HK2, HDAC7, SEMA7A, PREX1, PLAGL1, UBE2L6, CIART, FAM105A, TRIM37, ATP8A2, SIPA1, AHNAK, FURIN, SLC12A7, MARCKSL1, CD55, OSM, MXD1, LY6K, HMGN3, C19orf38, TLR7, KPNA2, TWF2, TMEM108, POLD4, ACTN2, UTF1, CHAC1, ATF6, CYSLTR2, RASGRP1, RAPGEF4, ACAA2, AHR, AEBP1, TNNT2, RNF157, RAVER1, BCL6, GM2A, STIM1, TPST1, TSPAN32, HVCN1, MGST2, KIT, EVA1B, CMTR1, IL12RB2, SLC25A15, CXCR4, SETX, RCN3, IL12RB1, PSMC3IP, CREM, SORCS2, USP31, EPB41L4B, TCP11L2, RETREG1, AHCY, TM6SF1, SYNE1, COQ4, HADH, ITGA6, CD40LG, ADD1, TNF, TTC28, MDK, SPATS2, C11orf98, FAAH, TNKS1BP1, BCL9L, MRPL53, HOOK2, LCN2, TIMM23, MEX3A, IFI44, FRMD8, STX11, TIMP2, PDCD1LG2, C17orf80, GOLM1, GSDME, ATP2A3, DHX37, HEXB, CSPG5, DGAT1, SGSM3, CCRL2, RAB37, ARRB2, S100A9, MMACHC, RASA3, SYCE2, MTA3, GPT2, CABLES1, CHST11, ACSS2, FAM185A, EPHX1, GRIN2C, MLKL, RACGAP1, MYO5A, PDZK1IP1, PCMTD2, PIPOX, ITGAE, PER2, NR6A1, FRY, MAP4K2, ZMIZ1, MICAL1, SUV39H2, LETM2, ACSS1, NLRC3, PINK1, IDH2, MTBP, MYO10, TANC1, AAED1, PHF11, KIF23, SLC28A2, TLCD1, OXCT1, NTRK3, AKAP1, CDC14B, LIF, BBC3, EMC9, BAG2, IGKC, ZNF428, FCGRT, GSTT2, MFSD2A, TNFAIP8L1, BSPRY, IFITM2, ST3GAL6, TMEM69, MYO1F, KLHL11, CENPA, NUTF2, LYPD6B, CXXC5, COX4I2, RFX3, KCNA2, IZUMO4, AK7, FKBP11, SGSH, RNF144A, STK35, IKZF4, AGRN, RHOB, COL9A3, PRNP, ABCA2, METTL27, MTURN, RFX1, CDC42EP3, TBXA2R, GAB3 |

| GENE SET | GENE SYMBOLS |
|---|---|
| IMMUNE_ DICTIONARY_ CD8_IL2 | GZMB, PPA1, EIF5A, NME1, NCL, SRM, PPP1R14B, PHGDH, RAN, RANBP1, CDK6, HSP90AA1, SHMT2, CYCS, EIF4A1, REXO2, YBX3, MIF, ANP32B, SOCS1, NPM3, PA2G4, SLC29A1, SLC25A5, TUBA1B, LDHA, MTHFD2, CDCA7, SET, CCND2, HSPD1, ATP5G1, MYBBP1A, SHMT1, ODC1, YBX1, DDX21, NOLC1, TUBA4A, TUBB4B, LARP1, IRF8, KLF2, C1QBP, HSPA8, DDX39A, GALK1, CCT3, SRSF2, ENO1, NOP56, MTHFD1, NHP2, MRTO4, NSUN2, PSAT1, PABPC4, TIMM8A, HSPA5, ST6GALNAC4, MAT2A, CISH, CAD, IARS, RPS27L, FABP5, EPRS, IPO5, EIF3A, EBNA1BP2, NOP58, MDN1, HSPA9, EIF2S2, SNRPD1, FBL, HNRNPAB, DCTPP1, IL7R, BCL3, NOC2L, SNU13, SRSF7, TOMM5, NOP16, G3BP1, MCM2, KARS, NARS, CACYBP, APEX1, ERH, GNL3, CANX, PCBP1, MPP6, DKC1, LTA, KPNB1, EIF3C, EIF3B, FKBP2, APRT, TMEM238, HSPA4, MRPS28, BANF1, IL2RB, YARS, EIF4G1, BZW1, ZNF593, SLC19A1, RRP15, AARS, CLUH, PRMT1, TOMM40, RRP1B, UQCC2, TSPAN4, RCC2, ACOT7, VARS, TPI1, LARS, RSL1D1, BYSL, UNG, TXN, LAP3, NAP1L1, SLC7A1, HDGF, WARS, CORO2A, RMI2, RGS10, NOP2, TIMM10, PUS7, LYAR, IPO4, TARS, RRS1, FASN, RPN1, SYNCRIP, PSME3, MRPL20, FAM162A, TIMM9, STAT3, BOP1, PHB, PPRC1, UCHL3, ATAD3A, RRP9, NDUFAB1, UTP20, PPAN, DTX1, ABCE1, EEF1E1, GCSH, PGK1, CLIC4, TSC22D3, PGAM1, UCK2, GAR1, HSP90B1, YPEL3, THOP1, ETF1, GART, IFRD2, MAGOHB, SLAMF7, PPID, METTL1, NEFH, EIF1AY, SAR1A, SMYD5, PUM3, JUN, RUVBL2, AIMP2, BZW2, BTG2, NIP7, SNRPA1, EIF2S1, FTSJ3, RUVBL1, CHEK1, MARS, MRPL12, CDK4, GBP6, CARS, RARS, BSPRY, NUDC, GADD45G, DNAJC2, MRPL17, XPOT, SFXN1, PDIA6, SMYD2, WDR12, SDF2L1, STIP1, CDV3, PTGES3, MYDGF, MTHFD1L, POLR1B, IL2RA, LILRB4, TMEM97, MTAP, STAT1, EPOP, SETBP1, LARP4, EIF4E, MANF, RPF2, HEATR1, RANGRF, IFNG, MCM5, CCDC86, ATIC, TCERG1, CDH1, AGPAT5, GNL1, NIFK, CNDP2, RRP12, POLR2H, MCM3, WDR77, PRPF31, CTPS1, PFKP, SSSCA1, LIF, PNO1, TUBA1C, PPAT, JAML, NOMO1, TMA16, GPATCH4, PIK3IP1, SEPT11, EIF1AX, HSPBP1, YWHAG, GARS, PUSL1, CD3EAP, TFDP1, DCTD, TBX21, RCL1, CINP, RCC1, UBL4A, USP36, AKT1, SLC35A4, ERAP1, NOL10, GBP2, FIGNL1, EIF2B3, GEMIN5, WDR46, PREP, PRPF19, EFHD2, ATP2A2, NOP14, PFAS, SMC4, CDC34, NAT10, SMARCC1, PCNA, TOMM70, TEX2, AGPAT3, NUP62, MFSD2A, AEN, KLF6, WDR18, S1PR1, DIS3, PITRM1, GBP7, TNK2, CHSY1, DAPL1, XBP1, AGFG1, CHCHD4, CAMKK2, SNRPA, NASP, POLR1A, ASNS, LRPPRC, POLR3D, GRWD1, UMPS, CAPG, YRDC, TSR1, UTP18, HYOU1, ACSL5, HELLS, COMTD1, LGALS3BP, PELP1, IRGM, KMT5A, LTV1, ID3, NOC4L, LRRC59, POLR2L, ZBP1, TIPIN, SMCO4, SYPL1, F2R, NEK6, TRMT61A, SYCE2, WDR3, P4HB, HK2, ZNHIT6, TFRC, MCM6, FEN1, FAM46A, SLC16A1, PPIF, DNAJC11, PLA2G12A, FAM136A, CRELD2, HIRIP3, CD160, DYNLL2, WEE1, DDX1, SZRD1, NLE1, SMPDL3A, IRF1, PDXK, NAMPT, ALDH18A1, KLRC1, ZMYND19, PRAG1, HBEGF, ARMC6, ACTN1, CDC25A, C7orf50, SERPINB9, FLT3LG, WDR4, WDR5, YDJC, NUP205, SLC35B1, WDR36, NUP93, BCAT1, TBL3, CCR5, CA12, NRARP, EIF4EBP1, UBQLN4, FOS, UHRF1, SLC7A5, DHX33, SCO2, SAMD1, ARL5C, EEF1AKMT4, ALG8, PWP1, SEMA7A, CD7, DTL, MEMO1, SPOUT1, TAF5L, PCGF6, CDCA7L, PWP2, CKS1B, PUS7L, CCDC58, NAF1, ERGIC1, RCN1, BHLHE40, SLAMF6, NME2, SOCS3, NAA25, BNIP3L, ISG20L2, GINS2, SLC25A33, ANKRD13B, PIM3, GSTO1, BAK1, ELAC2, RUNDC3B, PACSIN2, CCDC85C, UXS1, MAPKAPK2, PIM2, ACACA, RNF157, SLC25A22, CRLS1, SLC25A15, MTRR, NOL6, KAT2A, AMZ1, TRIM37, NCOA5, PCSK4, MOV10, IL12RB1, NOC3L, CHAF1A, S100A8, CD320, PTPRS, KIF21B, B4GALT5, FKBP5, FASLG, NOL9, MXD4, SPATA5, TMEM71, MAPK6, RCC1L, S100A9, SLC1A4, FCER1G, OAS3, KLHL24, LEO1, RIPK3, FAM185A, FXN, PRELID2, LRRC41, RBM19, MEX3A, CDKN1B, E2F6, FOXK2, TDRP, PPP1R15A, TMEM163, NSD2, AKAP1, MECR, CD274, CIART, PDCD1LG2, SLC35C2, RB1CC1, CARM1, ABI3, AEBP1, POLD4, LPCAT4, JAKMIP1, JPT2, NUDCD1, CXCR4, LMNB2, TDG, MID1, TYROBP, URB2, POP1, AMPD2, BSN, POLR3H, KLRB1, SENP3, IL15RA, GBP6, HIST1H1C, MGST2, GFM1, TTLL4, HMGA1, TADA2A, FPGS, NDC1, STYK1, TXNRD3, KPNA2, PHF10, PER2, PPP2R1B, XCL1, RMDN3, MAFK, HGH1, SUPV3L1, CCL2, CCDC102A, ABCC1, DAP, TP53RK, NUP188, NT5DC2, MAFF, TOP1MT, RAPGEF4, TNFSF10, ALPL, CENPS, TGM2, WDR74, CCR9, SHQ1, FAM129B, CASTOR1, AQP9, TSEN54, TNFRSF21, ATP23, ENOPH1, USP31, ABCB1, PRDX4, MCRIP2, BAG2, SLC29A2, TSPAN32, NUTF2, CYP51A1, SELENOP, CD55, LCN2, FKBP11, PRIM1, RCN3, GZMA, TMEM158, BBC3, TGIF2, F2RL2, SMPD4, TTLL12, EVA1B, JUNB, PCMTD1, EDARADD, LETM2, FNIP2, EXO1, NBN, TRDC, BEND3, HSPA1A, SUV39H2, CRYBG2, SOCS2, OSM, CD244, U2AF1L4, SCARB1, RHOQ, IZUMO4, ANKS1A, TXNRD2, SPIRE1, TPST1, IFT80, PLCXD2, TLE6, MTFP1, ACY1, SPRED1, NDST1, CCDC137, E2F1, DPAGT1, DNAJC27, PLK3, ADRM1, EMC9, MIER2, SMG8, NCR1, TNNT1, SLC25A13, TLCD1, IL12RB2, IL1R2, MS4A6A, STK39, DHRS7, TIMM23, NETO2, NR6A1, TNFSF11, GPRIN3, TNFRSF9, CENPA, ZNF367, DNA2, KCNQ5, CCL22, EPCAM, DSCC1, HIC1, FRAT2, KLHDC1, SGO1, PINK1, TBKBP1, AAED1, KLRB1, PTRH1, SLC5A6, PLP2, IFITM3, PTGER3, HBP1, FRMD4B, GBP4, ACSS2, ZNF566, LYPD6B, FAM46C, ART4, KCNA2, DHX37, RSAD1, GINS1, PLCG2, SRRD, TMEM150A, MCM10, TYW3, EEA1, LITAF, RACGAP1, PALB2, L2HGDH, LRP8, HEXB, MYO19, NDOR1, SPATA6, GCAT, LPL |
| IMMUNE_ DICTIONARY_ CD8_IL4 | HLA-DMA, DUSP10, IL4R, BCL2, SOCS1, PPA1, PHGDH, SHMT2, EIF5A, CDK6, CD8A, ENO1, MTHFD2, NOP56, EIF2S2, NCL, SRM, PLAC8, S100A10, NME1, GLIPR2, C1QBP, NCOA7, PPP3CC, AK2, NARS, PA2G4, CDCA7, CYCS, MBD2, FABP5, PPP1R14B, TUBA4A, METTL22, MARS, RANBP1, METTL1, CNP, CRIP1, TSPAN9, LYST, CARS, TCOF1, GNL3, EMP3, ERH, KLRD1, APEX1, NOP58, OTULIN, GALK1, CXCR6, LARS, TIMM8A, MRTO4, EPRS, DKC1, DDX21, PRPF31, PHB, AARS, KLF2, MYBBP1A, PRELID2, RMI2, EIF4EBP1, MRPS28, SLC7A1, GZMA, NCOA3, TMIE, GARS, PSAT1, NOLC1, SLC29A1, YARS, RRP15, SLC39A6, UCHL3, WDR43, RGS1, TIMM10, ST6GALNAC4, FOXN3, GAR1, GMFG, NOC2L, DDIT3, LAP3, PLA2G12A, EBNA1BP2, TOMM40, HS6ST1, RUVBL1, VIM, CD3EAP, UBL3, MTHFD1, WDR46, EEF1E1, IFRD2, DCTPP1, ATAD3A, ALDH18A1, NSUN2, RRS1, NIFK, NOP2, ZNF593, C12orf73, BYSL, MYC, VARS, TIMM9, NOP14, XBP1, UNG, NEFH, SMYD2, IARS, EOMES, UTP18, GRWD1, RRP9, PPAT, TPI1, RUVBL2, ALS2CL, IL7R, CDCA7L, SHMT1, ADK, RPF2, TMA16, GCSH, RNF19B, SCO2, RANGRF, GGACT, TARS, TRIB3, ZBP1, CLIC4, LSP1, WDR12, WDR74, PRPS1, CINP, S1PR1, HMBS, NOP16, SFT2D2, ZNHIT6, CTPS1, PUS7, DCTD, CISH, MCM2, CA12, NUP43, SUCLG2, SLC19A1, POLR3D, TRMT61A, ACTN1, ITGB7, EPOP, COMTD1, KIAA0040, PINX1, YDJC, |

| GENE SET | GENE SYMBOLS |
|---|---|
| | SPOUT1, SLC1A4, TRMT11, SLC16A2, UCK2, KLF6, RASGRP2, AHNAK, PUSL1, HIST1H4A, BTG2, RCN1, XPOT, GEMIN6, PROS1, XAF1, ZFP36L2, TECPR1, ACP5, TNP2, PCSK4, TMEM97, CAD, POLR3H, NME2, SMYD5, NFIL3, DYRK4, WARS, IPO4, YBX3, FAM46A, HLADMB, BIN1, F2R, TFDP1, ARHGAP26, DGKA, SLC7A5, PDXK, ISG20, SPECC1, PITRM1, TRAT1, YPEL3, TMTC4, PLXNA1, POLR1B, GEMIN5, THOP1, ETFBKMT, JUN, CDH1, CD7, CLUH, S100A11, TSC22D3, SCRN2, PECR, SLC25A33, AR, FOS, HGH1, MCRIP2, NEK1, TSPAN4, ABHD14A, CHAC1, SYCE2, OSBPL6, ANGPTL4, GADD45G, HSPA1A, IPCEF1, IL18R1, NEURL3, AQP9, ASNS, P2RX7, WEE1, KPNA2, TOP1MT, CIART, ARMC6, HK2, EFNA5, MTRR, FANCF, TADA2A, PDE7A, C7orf50, FRMD4B, SPN, TXNRD3, ARL5C, VCPKMT, ADGRE5, WDR59, C16orf74, PIK3IP1, SH2D1A, SLAMF6, GRAMD1A, BAG2, ARHGEF18, BSN, DDC, TRAF1, FRMD6, MXD4, MALT1, TP53INP1, SMPDL3A, KIF21B, SH3BP2, TMEM71, MADD, SLC12A7, CHEK1, LIPT2, TIMM23, ZNF566, RRAGD, FIGNL1, STIM1, NUTF2, CD55, RSAD1, TDRP, FOXO1, CDH13, MTFP1, AARSD1, THRB, SESN2, DUSP1, CXCR4, SLC6A9, POU2F2, SUV39H2, PYCR1, ESYT2, NETO2, S100A1, SELENOP, ZBTB20, GPT2, RGS2, SLC25A15, MYO19, PLEC, U2AF1L4, TLCD1, TUBA1A, CCL5, ACSS2, IKZF2, MYO1F, ST3GAL1, UBE2L6, DTX1, NEB, C19orf57, LRRC32, RNF144A, GATA1, CDKN2D, ALPL, BCL9L, JUNB, LRP8, SGK1, SNRNP35, MMACHC, GGT1, TPST1, FRMD8, MAP2, RAPGEF4, DCLRE1A, PGLYRP1, TGIF2, DOCK11, PRODH, ANXA2, RETREG1, GOLM1, JAKMIP1, DAP, F2RL2, DDIT4, ACY1, IGKC, SGIP1, RACGAP1, DNAJB1, ATP1B1, CD9, FAM69B, FRY, USP2, ZNF428, TRMT5, TECPR2, COL15A1, UPRT, HEXB, EHD3, RABGAP1L, OAS1, PRF1, ENG |
| IMMUNE_ DICTIONARY_ CD8_IL6 | IFIT3, ISG20, RSAD2, IFIT3, WDR90, PRIM1, MX1 |
| IMMUNE_ DICTIONARY_ CD8_IL7 | BCL2, EIF5A, REXO2, PPA1, PHGDH, RAN, BST2, PPP1R14B, ENO1, NME1, CDK6, SOCS1, NCL, SRM, ISG15, PA2G4, SHMT2, RANBP1, LTA, MTHFD2, EIF2S2, HSPD1, NOP56, C1QBP, CCT3, IL7R, JAML, GNL3, IFI35, NARS, TUBB4B, NOP58, MRTO4, SNRPD1, TUBA4A, CYCS, RTP4, EBNA1BP2, DKC1, TCF7, TIMM8A, METTL1, CARS, PIM1, DDX39A, CHCHD1, IGFBP4, BYSL, EIF2S1, KLRD1, PSAT1, RNF213, RRP9, SLC7A5, PLAC8, CISH, MYC, TIMM10, MARS, APEX1, GAR1, NOLC1, FKBP4, STIP1, SLC29A1, RGS10, IFRD2, MYBBP1A, HSPA9, SLC7A1, PHB, IARS, LAP3, PRPF31, RRP15, AIMP2, UCHL3, RRP1B, EPRS, ISG20, AARS, SMC4, NOC2L, ZBP1, EIF4EBP1, NSUN2, GALK1, RUVBL1, EEF1E1, RRS1, ATAD3A, DCTPP1, GARS, CORO2A, CNDP2, CDCA7, PPAN, TIMM9, CCDC86, NDUFAF4, XAF1, GADD45G, IRF7, UNG, ST6GALNAC4, GZMB, NOP16, NIFK, ZNF593, GCSH, WDR12, MAP3K8, BTG2, ODC1, LGALS3BP, MRPS18B, AGPAT3, ATP8A2, UTP18, CD3EAP, HERPUD1, YARS, TSC22D3, YPEL3, FTSJ3, OSM, GRWD1, NOP2, RPF2, SSSCA1, EIF1AX, IFIT1B, IPO5, NAF1, UCK2, ALDH18A1, MOV10, RMI2, PUSL1, LTV1, CLIC4, SOCS3, SIGMAR1, LARS, PNO1, TIMM50, SHMT1, TPI1, SLC19A1, RUVBL2, PUS7, RCL1, IFIT3, FLT3LG, IPO4, MTHFD1, COMTD1, TARS, AEN, UMPS, SMYD5, TMEM238, CD7, CTPS1, FASN, AATF, SETBP1, SCO2, ZNHIT6, DAPL1, NEFH, ARL5C, XPOT, PIK3IP1, USP18, PPAT, CHCHD4, POLR3D, TMEM158, NOC4L, ACTN1, YDJC, PRMT7, TMA16, TRMT61A, HSPA1A, FABP5, SMYD2, PIM2, CLUH, DCTD, TFAP4, TMEM97, MXD4, CD28, WARS, NEURL3, DDIT3, CXCR4, PRELID2, TRIB3, POLD2, RFLNB, TNF, FBXO17, ZDHHC8, MCM2, RRP12, PLA2G12A, USP50, ADAM19, IL2RA, CA12, HIRIP3, CIART, BCL3, ACOT7, WDR36, NOL10, SPATA5, RCC1, POLR1B, SLC30A4, TDRP, JUN, ARMC6, PIM3, CCR9, EPOP, DAP, DYRK4, PISD, C15orf48, TEX2, SLC1A4, MCM5, POLR3H, DHX58, HK2, TSPAN4, AMPD2, SOCS2, TMEM71, NSUN5, ZMYND19, FEN1, EEF1AKMT4, TP53INP1, FOS, IKZF4, SLC12A7, MRM3, GPR174, IFIT3, WEE1, THOP1, SCML4, IKBKE, CASTOR1, LIF, USP31, OAS2, SENP3, FAM46C, OAS1, SYNE1, PCSK4, CD55, ITGAE, ACSS2, TXNRD3, PCMTD2, LDLR, ASNS, LIPT2, SYCE2, CD79A, IFT80, LYPD6B, POP1, C7orf50, TADA2A, GOLM1, GSDME, PHF11, ACTN2, LMNB2, AMZ1, ZER1, GPT2, SLC25A15, RGS2, MTFP1, RAPGEF4, SNTB2, CHAC1, TTC28, SUV39H2, ST3GAL1, DZIP1, OASL, HERC3, RHOB, CHEK1, FBXL20, MYO1F, DTL, FIGNL1, CARD6, HBEGF, SLC26A11, TRIM5, SLC25A40, EMC9, RSAD2, CEP164, SGSH, MFSD2A, SQLE, HLA-DOA, NETO2, HSF4, TLE1, RCN3, CDC14B, SNX8, ABCA1, PCK2, CALCRL, SESN2, SLC28A2, PYCR1, TLR7, UBE3D, UBE2L6, EPHX1, PER2, TOB1, TFDP2, HID1, TLCD1, HSD17B11, BTBD11, RRAGD, MYO19, THRA, MYB, KCNA2, PHLDA3, NUTF2, ART4 |
| IMMUNE_ DICTIONARY_ CD8_IL12 | STAT1, SOCS1, IRF1, GBP6, GBP7, GBP2, PPA1, GADD45G, ZBP1, RTP4, PIM2, IRF8, ST6GALNAC4, IRGM, GBP4, SOCS3, BCL3, AHR, GZMB, CD274, GBP5, GBP6, PRNP, IFNG, FURIN, POP1, IL2RA, HSPA1A, C3orf33, SETBP1, PDP1, CXCR4, TNKS1BP1, PTGER3, FOS, HDAC6 |
| IMMUNE_ DICTIONARY_ CD8_IL27 | ZBP1, GBP2, PLAC8, IRF7, ISG15, IFIT3, XAF1, TSC22D3, FOS, BCL3, IL12RB1, GBP4, OAS1, CYSLTR2, IFIT3, GZMA, GADD45G, GBP6, IL18BP, GZMB, ITGAX, OAS2, RGS2 |
| IMMUNE_ DICTIONARY_ CD8_OSM | LARS2, IGFBP4, DTX1, SOCS3, TRNP1, BCL3, NEB, NME2, HSPA1A, ISG20, FOS, RSAD2, IFIT1B, SKAP2, USP18, JUN, KLF6, IFIT3, IFIT3, GADD45G, SLFN5, DHX58, CHST11, CMPK2, SERPINB1, RHOB, SPATS2, PPP1R15A, PRELID2, PHF11, IGSF23, DYNLT1, DUSP1 |
| IMMUNE_ DICTIONARY_ CD8_IL10 | BCL3, CEBPD |

| GENE SET | GENE SYMBOLS |
|---|---|
| IMMUNE_ DICTIONARY_ CD8_IL22 | FOS, HBB |
| IMMUNE_ DICTIONARY_ CD8_IFNB | ISG15, IFIT3, IFIT1B, ISG20, SLFN5, ZBP1, USP18, PHF11, RNF213, IRF7, BST2, RTP4, IFI16, RSAD2, XAF1, HERC6, GBP7, PPA1, IFI35, PARP14, GBP2, OAS3, DAXX, SAMHD1, JAML, EIF2AK2, IFI16, IFI16, DDX60, IFIT3, MX1, PLAC8, IFI16, TOR3A, TRAFD1, IFI16, PSMB10, OASL, DHX58, IFIT2, IFITM3, CMPK2, PSMB8, IFIH1, LY6E, HLA-E, STAT1, PARP9, GBP6, LGALS3BP, CCND2, SLFN13, GBP6, PHF11, TAPBP, IFIT1, SMCHD1, CXCL10, PML, SOCS1, DDX58, SAMD9L, PHF11, PSME1, OAS2, EPSTI1, CD86, CLIC4, IFI16, HELZ2, NMI, C19orf12, GADD45G, PSME2, OAS1, SELENOW, GZMA, CHMP4B, ATP8A2, TSPO, DTX3L, SP110, PARP12, THY1, STAT2, IFI16, NCOA7, CTSS, CASP8, PSMB9, ZUFSP, CRIP1, SLCO3A1, NAMPT, ASB13, GBP6, LGALS9, GBP5, UBA7, NAA20, USP25, GBP6, UBE2L6, CCRL2, HSPA5, EMP3, TREML2, TAP1, MOV10, TMBIM6, KLF2, TRIM25, S100A10, SDC3, ASCC3, IFI44, ZNFX1, ETNK1, CALHM6, KLRD1, PARP10, PARP11, MITD1, VARS, TOR1AIP1, TRIM5, CD274, CNP, TCF7, TLR7, GIMAP1-GIMAP5, MAX, RNF114, TBRG1, DDX24, DBNL, IRGM, ADAR, CYCS, EOMES, PLA2G16, ST6GALNAC4, MS4A6A, TMEM184B, GBP4, B4GALT5, TOR1AIP2, HSH2D, PCGF5, NLRC5, S1PR1, PHGDH, RASGRP2, PHIP, ZFP36L2, AIDA, TCOF1, HLA-A, ZCCHC2, ZNF365, CTSD, PHF11, TRIM6-TRIM34, SP140, PHC2, OGFRL1, DESI2, TDRD7, KEAP1, TNFSF10, RBL1, ARF4, TRIM21, IL18BP, GZMB, ITPR1, OGFR, GPR18, FAM111A, YPEL3, TAPBPL, LUZP1, TECPR1, TRIM56, TNP2, ACP5, PGD, IGFBP4, ADGRE5, MTHFD2, IFNGR1, IL12RB1, ETV6, LIPG, ACTN1, DUSP28, SLC25A22, CYSLTR2, PIM1, MORC3, AHNAK, SETDB2, FNBP4, RFC3, ATP10A, KIF21B, IRF9, PSME2, ARL5C, NEURL3, UBALD2, GBP6, FLNA, PNPT1, TMEM140, MVB12A, PLSCR3, CARS, RFLNB, ZYX, MXD4, RMI2, BCL3, HDAC7, MYD88, TTC39B, CD5, SLFN12L, SOCS3, PAPD7, MLKL, PDLIM2, CD7, TMEM192, GNG2, SLAMF7, C9orf3, AP1S3, STIM1, JAKMIP1, POU2F2, RBM43, MAP2K1, TLR3, SEPT9, FOS, IRF8, LGALS8, GMPPB, C6orf106, SNX2, C19orf66, TMEM106A, RABEPK, N4BP1, HK1, CISH, GCH1, PMEPA1, GRINA, SLFN13, RAMP1, GM2A, SLC7A1, GOLM1, SHMT2, CD200R1L, CXCR4, AARS, SLC7A5, ISOC1, CEBPB, XDH, HVCN1, ANKFY1, LAMP2, LASP1, IFITM10, EVI2A, MAFK, IARS, VPS54, ZMIZ1, DDIT3, P2RY14, BBC3, MYO1G, CXCR3, ATM, PPP1R15A, PGLYRP1, MAP7, TRIM14, NLRC3, PLCXD2, SGK1, IFI16, RETREG1, EHD4, CITED4, YARS, SIT1, APOBEC1, SSBP3, SORL1, SEMA7A, ANXA5, CPNE3, LIF, MAP3K8, TBXA2R, RCN3, PPM1K, PHLPP1, PEAK1, OSM, UBALD1, COX18, SNTB2, SH3BP2, ACTN2, WARS, PRKCE, STOML1, ARHGAP26, CCNYL1, STK26, OR2V1, VWA5A, ABCA2, CITED2, INSL6, CD72, SGSH, CD55, CCDC107, RACGAP1, UBASH3B, MYO1F, SLC17A9, RGS2, FURIN, SYNE1, TRIM5, CEP164, DHRS7, NBEAL2, CXXC5, HADH, IL10RA, TTC28, CDC42EP3, TPCN1, G0S2, SERPINI1, CARD19, GRN, ELK3, RHOB, SKI, N4BP2, TRIB2, SLC12A6, ILDR1, IZUMO4, BTBD11, MTM1, PLEKHG2, PPCDC, PINK1, SLC26A11, GPSM2, MIER3, GPRIN3, CENPA, MAST3, ITGA6, SYTL2, PIGA, CMTR1, ACSS2, TNS1, RASA4, SFXN3, ITGAE, NMRAL1, DUSP1, CD44, ZFC3H1, ANXA2, GALNT10, MAN2A2, CSTA, CGAS, PRELID2, TMIE, PGM2L1, FCGRT, SESN1, MAPK3, GNG12, SH3GLB2, MILR1, MYB, LITAF, LPIN1, ADAMTS6, CD9, TOB1, PCMTD2, CDC14B, FBXL20, AAED1, CDC25B, ZER1, NUDT14, PKD1, TUBA8, MTURN, CASP4, MAPRE2, CEP97, QRFP, FRY, HID1, PEA15, RNF157, IFNG, RANBP10, LMO2, CXorf21, RNF130, HS3ST3B1, SMAD3, IL15RA, ZFHX2, AGRN, PLEKHA5, ITM2A, GOLIM4, SAMD1, ARHGAP5, PAQR7, TAGAP, SLC25A40, ARMC3, NRM, RTN4RL1, SKAP2, MCEE, CTDSP2, LATS2, DTWD1, RASSF3, IL3RA, ETHE1, TRIB3, ABTB1, PLIN3, ARRB1, HDAC5, LAG3, GSN, STK39, TESK1, PACS2, P2RX4, METTL27, MOSPD3, MXRA8, SLC2A3, IZUMO1R, SPECC1, DHRS1, SLC43A2, VIPR1, SLC28A2, CHKA, FAM168A, TMEM141, IFNGR2, TRAT1, FRAT2, CHIC1, NR4A2, LRRC75B, TNFAIP8L1, KCNJ8, PXYLP1, STRADA, SPICE1, ARHGAP18, HIST4H4, MAP3K3, HIST1H1D, HAUS4, SFMBT2, ALS2, WBP1, FAP, SHISA2, GAS7, CALCRL, ELOVL7, AGFG2, COL23A1, NIPAL3, ACCS, MNS1, CHAC1, UNC5CL, DDC, CASTOR1, SOX4 |
| IMMUNE_ DICTIONARY_ CD8_IFNG | GBP2, GBP6, STAT1, GBP7, ZBP1, IRF1, SAMHD1, RTP4, ISG15, SOCS1, GBP6, BST2, CD274, IFIT3, GBP6, IRGM, TAPBPL, GBP6, RNF213, PARP14, GBP5, IRF7, PARP9, DTX3L, PPA1, IL18BP, PLA2G16, XAF1, GBP4, IRF8, ISG20, IFIT1B, GBP6, NAMPT, OAS3, WARS, USP18, IFIT3, INSL6, TRAFD1, ST6GALNAC4, PARP10, IFI44, STAT2, IFIH1, OAS1, FOS, UBE2L6, PLSCR3, BCL3, NME2, CXCL10, CMPK2, MTUS2 |
| IMMUNE_ DICTIONARY_ CD8_TNFA | IGFBP4, BCL3, CRIP1, NFKBIA, VARS, KLF2, RELB, PPA1, NFKB2, DDIT4, ZBP1, SOCS3, MAPKAPK2, GBP2, GADD45G, CARS, SDHAF1, JUN, ISG15, BIRC3, FKBP5, KLF6, TRNP1, BTG2, GZMB, BATF, AARS, SHMT2, XAF1, HSPA1A, ODC1, FOS, MTHFD2, NFKBIE, IARS, BBC3, MARS, ARID5B, CLIC4, ETV6, SKAP2, ST6GALNAC4, ICAM1, TNFRSF9, YARS, ISG20, ADGRE5, GBP5, FAM46A, MXD4, NEB, INSL6, PRELID2, PLSCR3, C15orf48, PLCG2, CYSLTR2, TRIB3, KSR1, EIF4EBP1, PPP1R15A, SLC7A5, GZMA, RHOB, SERPINB1, RGS2, CD55, CHST11, SLC1A4, SPATS2, SHMT1, CABLES1, MID1IP1, EPOP, ACTN2, SLC28A2, S100A6, CDC25B, CD83, CCL5, IFITM10, EMC9, C3orf33, IL12RB1, FOSB, TOB1, PDCD1LG2, NR4A2, VIPR1, OAS2, MYO10, CIART, ASNS, IL18BP, NOX1, EPHX1, ABCB6, SYT6 |
| IMMUNE_ DICTIONARY_ CD8_TL1A | NFKBIA, RELB, NFKB2, ICAM1, CD82, BCL3, IKBKE, CD83, NFKBIE, BIRC3, TNFRSF9, TNFRSF25, UTF1, MGLL, FOS, LAD1, HSPA1A, GADD45G, ULBP1, PDCD1LG2, LIPT1, EFCAB2, DENND5A, SORCS2, DUSP1, ADAMTSL2, ZBTB46, CMTR2 |

## SUPPLEMENTAL REFERENCES


1. Villeneuve A, et al. Multiple organ dysfunction syndrome in critically ill children: clinical value of two lists of diagnostic criteria. *Ann Intensive Care.* 2016;6(1):40.
2. Goldstein B, et al. International pediatric sepsis consensus conference: definitions for sepsis and organ dysfunction in pediatrics. *Pediatr Crit Care Med.* 2005;6(1):2-8.
3. Schlapbach LJ, et al. International Consensus Criteria for Pediatric Sepsis and Septic Shock. *JAMA.* 2024;331(8):665-74.
4. Zimmerman JJ, et al. Critical Illness Factors Associated With Long-Term Mortality and Health-Related Quality of Life Morbidity Following Community-Acquired Pediatric Septic Shock. *Crit Care Med.* 2020;48(3):319-28.
5. Meert KL, et al. Trajectories and Risk Factors for Altered Physical and Psychosocial Health-Related Quality of Life After Pediatric Community-Acquired Septic Shock. *Pediatr Crit Care Med.* 2020;21(10):869-78.
6. John CR, et al. M3C: Monte Carlo reference-based consensus clustering. *Sci Rep.* 2020;10(1):1816.
7. Fine JP, and Gray RJ. A Proportional Hazards Model for the Subdistribution of a Competing Risk. *J Am Stat Assoc.* 1999;94(446):496-509.
8. Wurm MJ, et al. Regularized Ordinal Regression and the ordinalNet R Package. *J Stat Softw.* 2021;99(6).
9. Van Calster B, et al. Extending the c-statistic to nominal polytomous outcomes: the Polytomous Discrimination Index. *Stat Med.* 2012;31(23):2610-26.
10. Chen H, et al. Cytofkit: A Bioconductor Package for an Integrated Mass Cytometry Data Analysis Pipeline. *PLoS Comput Biol.* 2016;12(9):e1005112.
11. Monaco G, et al. flowAI: automatic and interactive anomaly discerning tools for flow cytometry data. *Bioinformatics.* 2016;32(16):2473-80.
12. Van Gassen S, et al. FlowSOM: Using self-organizing maps for visualization and interpretation of cytometry data. *Cytometry A.* 2015;87(7):636-45.
13. D. M. Chan RR, F. Huang and J. F. Canny,. *2018 30th International Symposium on Computer Architecture and High Performance Computing*. Lyon, France: IEEE; 2018:330-8.
14. Kramer A, et al. Causal analysis approaches in Ingenuity Pathway Analysis. *Bioinformatics.* 2014;30(4):523-30.
15. Subramanian A, et al. Gene set enrichment analysis: a knowledge-based approach for interpreting genome-wide expression profiles. *Proc Natl Acad Sci U S A.* 2005;102(43):15545-50.
16. Hanzelmann S, et al. GSVA: gene set variation analysis for microarray and RNA-seq data. *BMC Bioinformatics.* 2013;14:7.
17. Liberzon A, et al. The Molecular Signatures Database (MSigDB) hallmark gene set collection. *Cell Syst.* 2015;1(6):417-25.
18. Andreatta M, and Carmona SJ. UCell: Robust and scalable single-cell gene signature scoring. *Comput Struct Biotechnol J.* 2021;19:3796-8.
19. Hao Y, et al. Dictionary learning for integrative, multimodal and scalable single-cell analysis. *Nat Biotechnol.* 2024;42(2):293-304.
20. Hao Y, et al. Integrated analysis of multimodal single-cell data. *Cell.* 2021;184(13):3573-87 e29.

21. Jin S, et al. Inference and analysis of cell-cell communication using CellChat. *Nat Commun.* 2021;12(1):1088.
22. Borcherding N, et al. scRepertoire: An R-based toolkit for single-cell immune receptor analysis. *F1000Res.* 2020;9:47.
23. Shannon C. A Mathematical Theory of Communication. *The Bell System Technical Journal.* 1948;27:379-423.
24. Borcherding N, et al. CD4(+) T cells exhibit distinct transcriptional phenotypes in the lymph nodes and blood following mRNA vaccination in humans. *Nat Immunol.* 2024;25(9):1731-41.
25. Cui A, et al. Dictionary of immune responses to cytokines at single-cell resolution. *Nature.* 2024;625(7994):377-84.
26. Kane M, and Esther SY. Using Cliff's Delta as a Non-Parametric Effect Size Measure: An Accessible Web App and R Tutorial. *Practical Assessment, Research, and Evaluation.* 2024;29(1).
27. Proulx F, et al. The pediatric multiple organ dysfunction syndrome. *Pediatr Crit Care Med.* 2009;10(1):12-22.
